# A Comprehensive Study Concerning the Synthesis, Structure, and Reactivity of Terminal Uranium Oxido, Sulfido, and Selenido Metallocenes

**DOI:** 10.1021/jacs.3c03753

**Published:** 2023-06-28

**Authors:** Tongyu Li, Dongwei Wang, Yi Heng, Guohua Hou, Guofu Zi, Wanjian Ding, Marc D. Walter

**Affiliations:** † Department of Chemistry, Beijing Normal University, Beijing 100875, China; ‡ Institut für Anorganische und Analytische Chemie, Technische Universitüt Braunschweig, Hagenring 30, 38106 Braunschweig, Germany

## Abstract

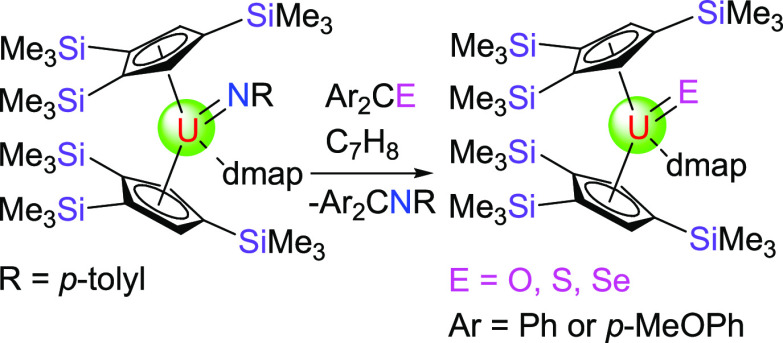

Terminal uranium oxido, sulfido, and selenido metallocenes were synthesized, and their reactivity was comprehensively studied. Heating of an equimolar mixture of [η^5^-1,2,4-(Me_3_Si)_3_C_5_H_2_]_2_UMe_2_ (**2**) and [η^5^-1,2,4-(Me_3_Si)_3_C_5_H_2_]_2_U(NH-*p*-tolyl)_2_ (**3**) in the presence of 4-dimethylaminopyridine (dmap) in refluxing toluene forms [η^5^-1,2,4-(Me_3_Si)_3_C_5_H_2_]_2_U=N(*p*-tolyl)(dmap) (**4**), which is a useful precursor for the preparation of the terminal uranium oxido, sulfido, and selenido metallocenes [η^5^-1,2,4-(Me_3_Si)_3_C_5_H_2_]_2_U=E(dmap) (E = O (**5**), S (**6**), Se (**7**)) employing a cycloaddition–elimination methodology with Ph_2_C=E (E = O, S) or (*p*-MeOPh)_2_CSe, respectively. Metallocenes **5**–**7** are inert toward alkynes, but they act as nucleophiles in the presence of alkylsilyl halides. The oxido and sulfido metallocenes **5** and **6** undergo [2 + 2] cycloadditions with isothiocyanate PhNCS or CS_2_, while the selenido derivative **7** does not. The experimental studies are complemented by density functional theory (DFT) computations.

## Introduction

**Figure 1:**
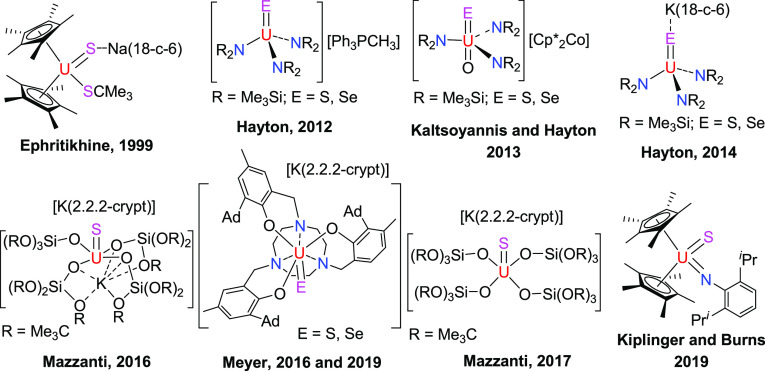
Selected crystallographically authenticated terminal uranium sulfido and selenido species.

Attributed to their unique structural properties and their potential applications in group transfer and catalysis, organoactinide complexes featuring terminal metal–ligand multiple bonds have been intensively studied over the past two decades.^1^ These research activities focused on oxido and chalcogenido organoactinide complexes in particular^1−5^ due to the ubiquity of these functionalities in actinide chemistry, as shown by the prevalence of binary oxides and sulfides in the solid state.^6^ In this context, well-defined molecular structures will not only advance our understanding of the bonding and reactivity of the An=E (O, S, Se, Te) functional groups but also enable us to uncover novel and potentially useful transformations. For example, chlorocarbons convert on solid U_3_O_8_ to yield CO_x_ and HCl.^7^ This unusual reaction occurs at the U–O moieties on the U_3_O_8_ surface. Over the years, many oxido organouranium complexes have been prepared, which certainly enhanced the structural library, but their intrinsic reactivity was rather limited.^2,3^ One notable exception, however, constitutes [η^5^-1,2,4-(Me_3_C)_3_C_5_H_2_]_2_U=O whose reactivity toward alkyl halides was studied in more detail.^3e^ Moving to the heavier chalcogenides S and Se, structurally authenticated terminal sulfido complexes have so far been limited to the derivatives, such as [Na(18-crown-6)][(η^5^-Me_5_C_5_)_2_U^IV^(=S)(SCMe_3_)],^5a^ [Ph_3_PMe][U^IV^(=S)[N(SiMe_3_)_2_]_3_],^5b^ [(η^5^-C_5_Me_5_)_2_Co][U^VI^(=S)(=O)[N(SiMe_3_)_2_]_3_],^5d^ [K(18-crown-6)][U^IV^(=S)[N(SiMe_3_)_2_]_3_],^5e^ [(S=)U^IV^(OSi(O^*t*^Bu)_3_)_4_K][K(2.2.2-crypt)],^5h^ [((^Ad,Me^ArO)_3_tacn)U^IV^(=S)][K(2.2.2-crypt)],^5i^ [K(2.2.2-cryptand)][U^V^(=S){OSi(O^*t*^Bu)_3_}_4_],^5k^ and (η^5^-C_5_Me_5_)_2_U^VI^(=S)( =N-2,6-^*i*^Pr_2_C_6_H_3_) (Figure 1),^5l^ and only a few examples of the uranium complexes containing a terminal selenido functionality, such as [Ph_3_PMe][U^IV^(=Se)[N(SiMe_3_)_2_]_3_],^5b^ [(η^5^-C_5_Me_5_)_2_Co][U^VI^(=Se)( =O)[N(SiMe_3_)_2_]_3_],^5d^ [K(18-crown-6)][U^IV^(=Se)(NR_2_)_3_],^5f^ and [K(2.2.2-crypt)][((^Ad,Me^ArO)_3_tacn)U^IV^(=Se)],^5m^ have been reported (Figure 1). Moreover, the reactivity of the terminal sulfido and selenido actinide complexes has so far remained elusive and this also extends to the underlying structure–reactivity relationship.^5^ This renders the synthesis of novel actinide oxido and chalcogenido metallocenes an interesting but still challenging synthetic target for which bulky ligands are indispensable.^3e^ Besides structural aspects and the reactivity of organoactinide complexes in small molecule activation,^8^ research at the bottom of the periodic table is also driven by the more fundamental question dealing with the impact of the 6d and 5f orbitals on structure, bonding, and reactivity.^9^ Following up on these research lines, we have investigated actinide complexes featuring terminal metal–ligand multiple bonds and explored their reactivity.^10^ For these studies, the sterically encumbered cyclopentadienyl ligand, 1,2,4-(Me_3_C)_3_C_5_H_2_, has been our ligand of choice stabilizing the terminal actinide oxido metallocenes [η^5^-1,2,4-(Me_3_C)_3_C_5_H_2_]_2_An^IV^=O(dmap) (An = Th, U), which readily react with small molecules such as Me_3_SiCl, Ph_2_CE (E = O, S) and CS_2_.^3e,10b^ Furthermore, only two examples of terminal actinide sulfido metallocenes and no examples of terminal actinide selenido metallocenes have been structurally authenticated,^5a,5l^ so we decided to extend these investigations to terminal uranium oxido and chalcogenido metallocenes featuring two 1,2,4-(Me_3_Si)_3_C_5_H_2_ ligands. Although 1,2,4-(Me_3_Si)_3_C_5_H_2_ is closely related to its counterpart 1,2,4-(Me_3_C)_3_C_5_H_2_, this ligand is less sterically demanding and more electron-deficient than 1,2,4-(Me_3_C)_3_C_5_H_2_. In this contribution, we detail the synthesis of the terminal uranium oxido, sulfido, and selenido complexes, [η^5^-1,2,4-(Me_3_Si)_3_C_5_H_2_]_2_U^IV^=E(dmap) (E = O (**5**), S (**6**), Se (**7**)), and their reactivity. We also compare the reactivity of these derivatives to those of thorium(IV) and group 4 metallocenes.

## Results and Discussion

### Synthesis of [η^5^-1,2,4-(Me_3_Si)_3_C_5_H_2_]_2_U^IV^=E(dmap) (E = O (5), S (6), Se (7))

**Figure 2:**
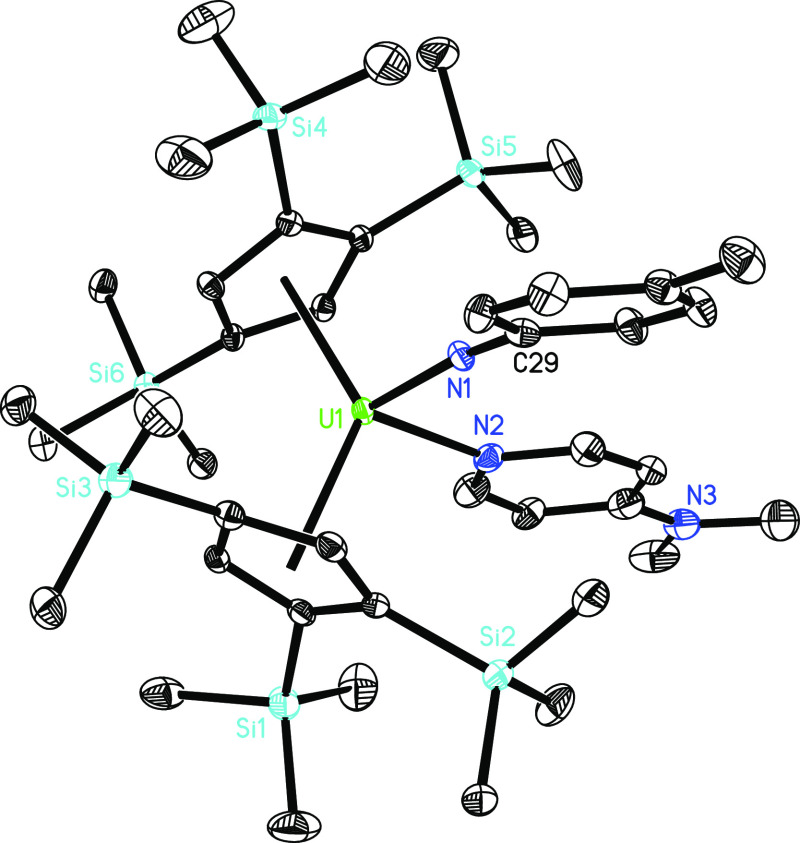
Molecular structure of **4** (thermal ellipsoids drawn at the 35% probability level).

**Figure 3:**
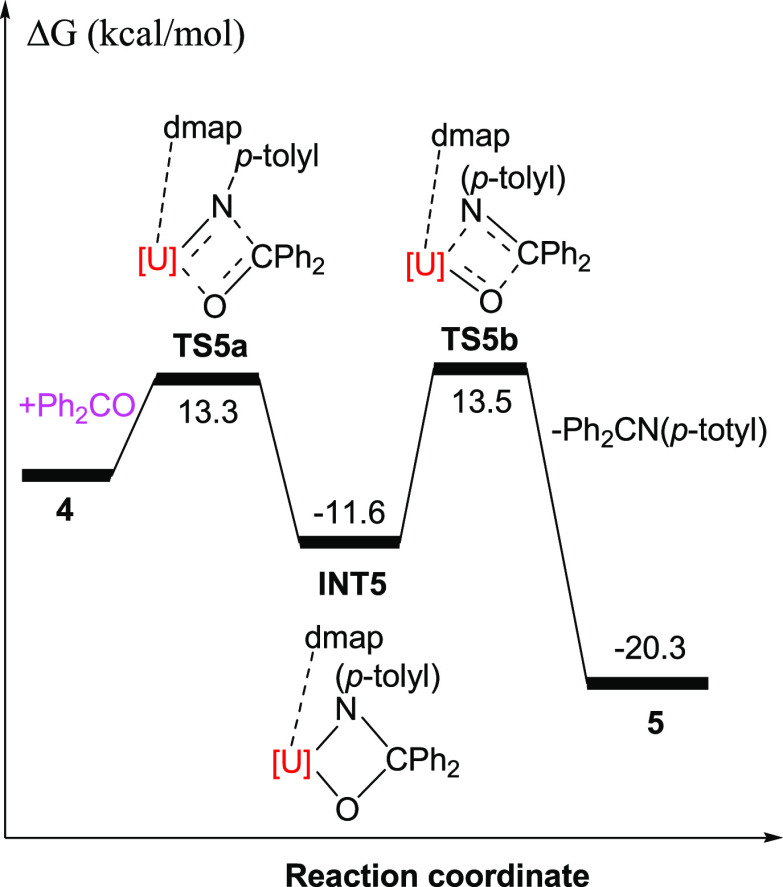
Energy profile (kcal/mol) for the reaction of **4** + Ph_2_CO (computed at *T* = 298 K). [U] = [η^5^-1,2,4-(Me_3_Si)_3_C_5_H_2_]_2_U.

**Figure 4:**
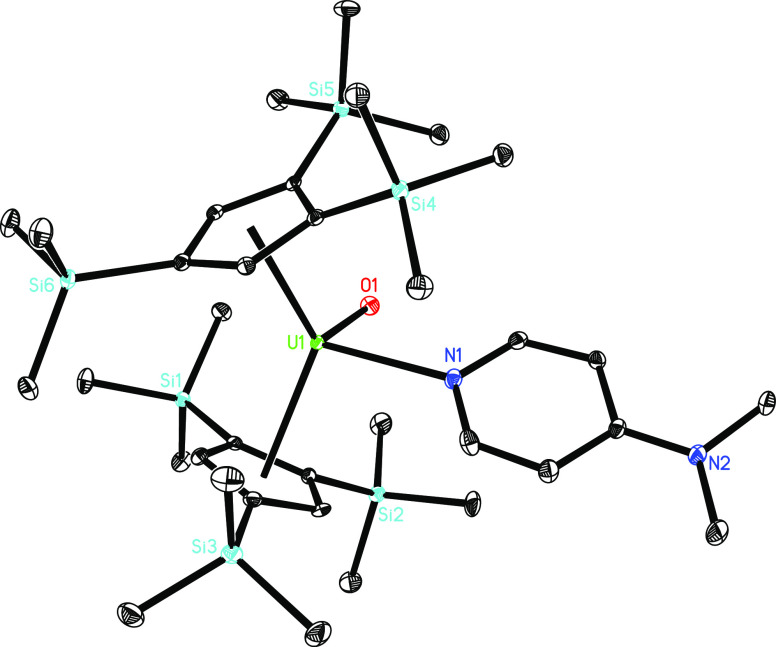
Molecular structure of **5** (thermal ellipsoids drawn at the 35% probability level).

**Figure 5:**
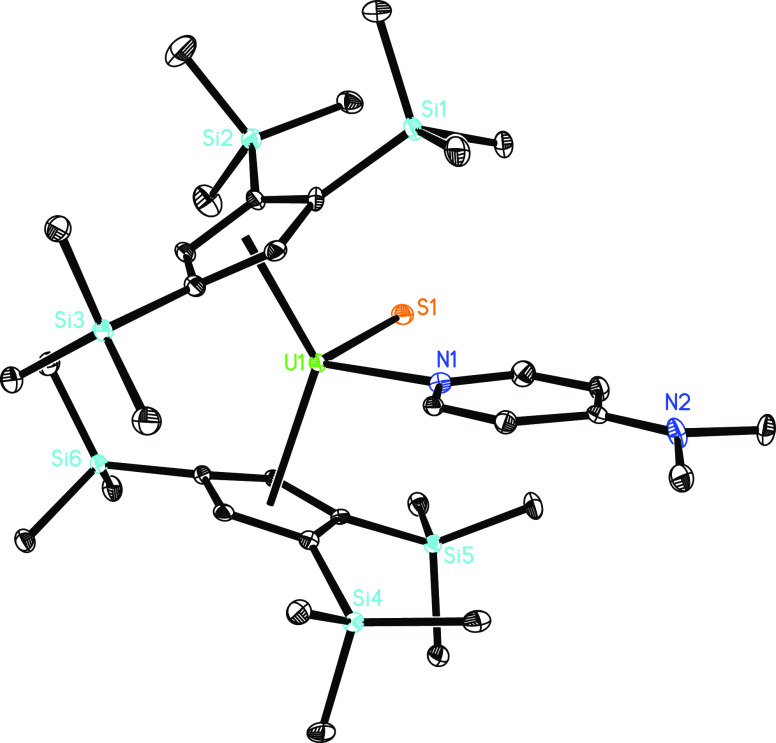
Molecular structure of **6** (thermal ellipsoids drawn at the 35% probability level).

**Figure 6:**
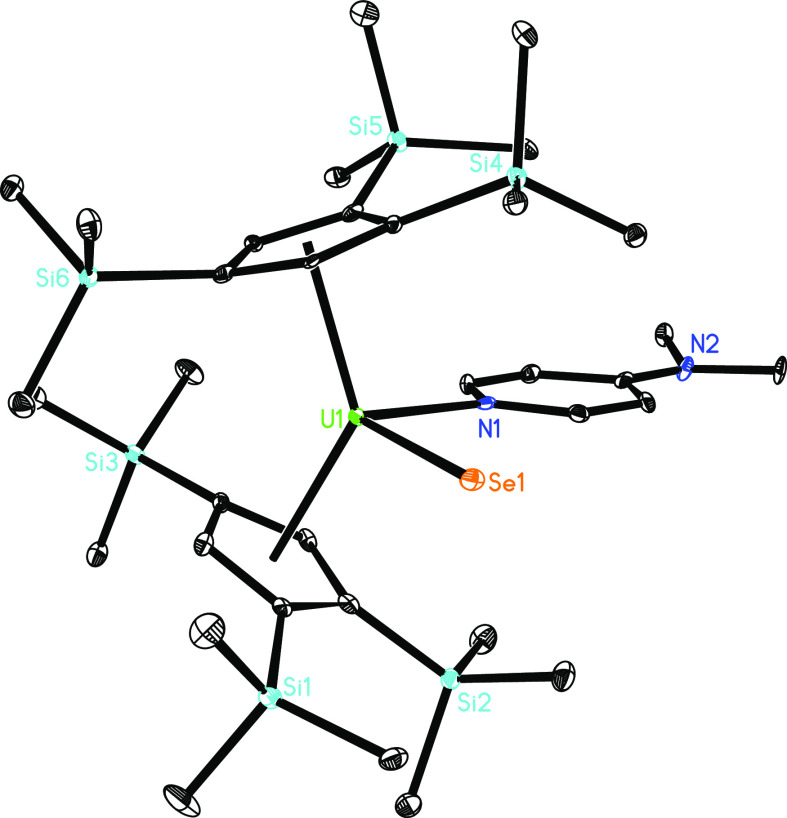
Molecular structure of **7** (thermal ellipsoids drawn at the 35% probability level).

**Scheme 1:**
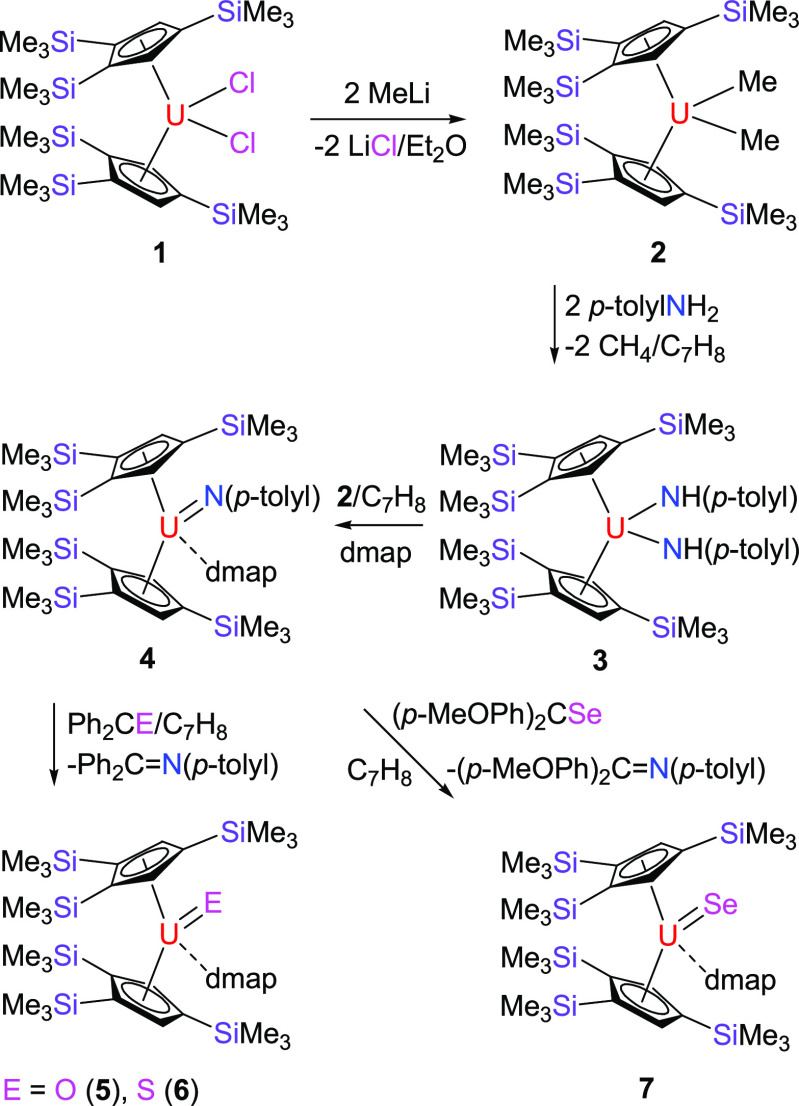
Synthesis of Complexes **2**–**7**

Salt metathesis between [η^5^-1,2,4-(Me_3_Si)_3_C_5_H_2_]_2_UCl_2_ (**1**) and 2 equiv of MeLi in diethyl ether affords [η^5^-1,2,4-(Me_3_Si)_3_C_5_H_2_]_2_UMe_2_ (**2**) in 82% yield. The subsequent reaction of **2** with 2 equiv of *p*-toluidine in toluene gives [η^5^-1,2,4-(Me_3_Si)_3_C_5_H_2_]_2_U(NH-*p*-tolyl)_2_ (**3**) in excellent yield (93%). Heating of an equimolar mixture of **2** and **3** in the presence of 4-dimethylaminopyridine (dmap) in refluxing toluene proceeds cleanly furnishing the uranium imido complex [η^5^-1,2,4-(Me_3_Si)_3_C_5_H_2_]_2_U=N(*p*-tolyl)(dmap) (**4**) in 87% yield (Scheme 1). The molecular structure of **4** is shown in Figure 2; for selected bond distances and angles, refer to Table 1. Complex **4** features two very different U–N bond distances of 2.426(6) and 2.021(5) Å for U-N(2) and U-N(1), respectively, which are consistent with a dative bond between the dmap ligand and the U(IV) atom and the formation of a uranium–imido complex, respectively. A similar uranium–imido bond distance of 1.988(5) Å was found in the closely related derivative [η^5^-1,2,4-(Me_3_C)_3_C_5_H_2_]_2_U=N(*p*-tolyl).^3e^ In line with the reaction of the actinide imidos [η^5^-1,2,4-(Me_3_C)_3_C_5_H_2_]_2_An=N(*p*-tolyl) (An = Th, U) with Ph_2_CO,^3e,10b^ treatment of **4** with 1 equiv of Ph_2_CO results in the isolation of the terminal oxido derivative, [η^5^-1,2,4-(Me_3_Si)_3_C_5_H_2_]_2_U=O(dmap) (**5**), in good yield (Scheme 1). DFT investigations imply that **4** initially engages in a [2 + 2] cycloaddition with Ph_2_C=O to give the heterocyclic intermediate **INT5**. The formation of **INT5** is energetically favorable (Δ*G*(298 K) = −11.6 kcal/mol) and proceeds via the transition state **TS5a** with a reaction barrier of Δ*G*^‡^(298 K) = 13.3 kcal/mol (Figure 3). However, the degradation of **INT5** to form the oxido compound **5** and Ph_2_C=N(*p*-tolyl) is more thermodynamically preferred (Δ*G*(298 K) = −20.3 kcal/mol) and proceeds via the transition state **TS5b** with a reaction barrier of Δ*G*^‡^(298 K) = 25.1 kcal/mol (Figure 3). This profile is consistent with the experimentally observed formation of **5** at ambient temperature. The molecular structure of **5** is provided in Figure 4; for selected bond distances and angles, see Table 1. The U–O and U–N distances are 1.873(2) and 2.526(3) Å, respectively, which are comparable to those established for [η^5^-1,2,4-(Me_3_C)_3_C_5_H_2_]_2_U=O(dmap) with a U–O distance of 1.860(3) Å and U–N distance of 2.535(4) Å^3e^ and [η^5^-1,2,4-(Me_3_C)_3_C_5_H_2_]_2_U=O(py) with a U–O distance of 1.874(4) Å and U–N distance of 2.589(5) Å.^11^ Nevertheless, the Cp(cent)–U–Cp(cent) angle is 128.2(1)°, which is smaller than those found in [η^5^-1,2,4-(Me_3_C)_3_C_5_H_2_]_2_U=O(dmap) (141.7(4)°)^3e^ and [η^5^-1,2,4-(Me_3_C)_3_C_5_H_2_]_2_U=O(py) (139.2(2)°).^11^ This suggests a much more open coordination sphere at the uranium atom in **5**, which allows stabilizing ligands to coordinate, and therefore this difference reflects the reactivity of these complexes.^3e^ In contrast to the reaction of the thorium derivative [η^5^-1,2,4-(Me_3_C)_3_C_5_H_2_]_2_Th=N(*p*-tolyl) with Ph_2_CS in the presence of dmap,^10b^ the terminal uranium sulfido metallocene, [η^5^-1,2,4-(Me_3_Si)_3_C_5_H_2_]_2_U=S(dmap) (**6**), can be isolated from the reaction of **4** with 1 equiv of Ph_2_CS in good yield (Scheme 1). Figure 5 shows the molecular structure of **6** and selected bond distances and angles are provided in Table 1. Complex **6** is the third representative of structurally authenticated actinide sulfido metallocenes and is therefore a significant addition to the other two actinide sulfido derivatives, [Na(18-crown-6)][(η^5^-Me_5_C_5_)_2_U^IV^(=S)(SCMe_3_)] and (η^5^-C_5_Me_5_)_2_U^VI^(=S)(=N-2,6-^*i*^Pr_2_C_6_H_3_).^5a,5l^ The U–N distance of 2.509(5) Å is comparable to those found for compounds **4** and **5** (Table 1). The U–S distance of 2.437(1) Å can be related to those found in [Na(18-crown-6)][(η^5^-Me_5_C_5_)_2_U^IV^(=S)(SCMe_3_)] (2.477(2) and 2.462(2) Å),^5a^ [Ph_3_PMe][U^IV^(=S)[N(SiMe_3_)_2_]_3_] (2.4805(5) Å),^5b^ [(C_5_Me_5_)_2_Co][U^VI^(=S)(=O)[N(SiMe_3_)_2_]_3_] (2.390(8) Å),^5d^ [K(18-crown-6)][U^IV^(=S)[N(SiMe_3_)_2_]_3_] (2.4463(6) Å),^5e^ [(S=)U^IV^(OSi(O^*t*^Bu)_3_)_4_K][K(2.2.2-crypt)] (2.5220(14) Å),^5h^ [((^Ad,Me^ArO)_3_tacn)U^IV^(=S)][K(2.2.2-crypt)] (2.536(2) Å),^5i^ [K(2.2.2-cryptand)][U^V^(=S){OSi(O^*t*^Bu)_3_}_4_] (2.376(5) Å),^5k^ and (η^5^-C_5_Me_5_)_2_U^VI^(=S)(=N-2,6-^*i*^Pr_2_C_6_H_3_) (2.363(1) Å),^5l^ further supporting the formation of a uranium metallocene featuring a terminal U=S bond. Moreover, treatment of **4** with 1 equiv of (*p*-MeOPh)_2_CSe yields the terminal selenido derivative, [η^5^-1,2,4-(Me_3_Si)_3_C_5_H_2_]_2_U=Se(dmap) (**7**) (Scheme 1). Figure 6 shows the molecular structure of **7**; for selected bond distances and angles, see Table 1. To the best of our knowledge, **7** constitutes the first structurally authenticated terminal selenido actinide metallocene, and it also expands the limited family of structurally characterized actinide selenido complexes, comprising [Ph_3_PMe][U^IV^(=Se)[N(SiMe_3_)_2_]_3_],^5b^ [(C_5_Me_5_)_2_Co][U^VI^(=Se)( =O)[N(SiMe_3_)_2_]_3_],^5d^ [K(18-crown-6)][U^IV^(=Se)(NR_2_)_3_],^5f^ and [K(2.2.2-crypt)][((^Ad,Me^ArO)_3_tacn)U^IV^(=Se)].^5m^ The U–N distance of 2.507(4) Å is unremarkable and close to those found for compounds **4**–**6** (Table 1). Furthermore, the U–Se distance of 2.583(1) Å is in the range established for [Ph_3_PMe][U^IV^(=Se)[N(SiMe_3_)_2_]_3_] (2.6463(7) Å),^5b^ [(C_5_Me_5_)_2_Co][U^VI^(=Se)( =O)[N(SiMe_3_)_2_]_3_] (2.533(1) Å),^5d^ [K(18-crown-6)][U^IV^(=Se)(NR_2_)_3_] (2.585(1) and 2.595(1) Å),^5f^ and [K(2.2.2-crypt)][((^Ad,Me^ArO)_3_tacn)U^IV^(=Se)] (2.695(2) Å).^5m^

**Table 1:** Selected Distances (Å) and Angles (°) for Compounds **4**–**10**, **12**–**15**, and **17**^a^

compound	C(Cp)–U^b^	C(Cp)–U^c^	Cp(cent)–U^b^	U–X	Cp(cent)–U–Cp(cent)	X–U–X/Y
**4**	2.809(6)	2.745(6) to 2.864(6)	2.533(6)	N(1) 2.021(5), N(2) 2.426(6)	130.8(2)	93.2(2)
**5**	2.836(3)	2.741(3) to 2.945(3)	2.564(3)	O(1) 1.873(2), N(1) 2.526(3)	128.2(1)	87.2(1)
**6**	2.788(7)	2.741(6) to 2.824(6)	2.511(6)	S(1) 2.437(1), N(1) 2.509(5)	134.0(2)	95.9(1)
**7**	2.784(4)	2.741(5) to 2.827(5)	2.505(5)	Se(1) 2.583(1), N(1) 2.507(4)	135.2(2)	96.5(1)
**8**	2.758(4)	2.714(4) to 2.803(4)	2.476(4)	O(1) 2.071(3), Cl(1) 2.820(1)	129.2(1)	91.8(1)
**9**	2.776(6)	2.727(6) to 2.823(5)	2.493(3)	O(1) 2.077(3), I(1) 2.963(1)	128.3(2)	93.3(1)
**10**	2.757(4)	2.698(3) to 2.807(4)	2.474(3)	O(1) 2.067(2), N(1) 2.525(4)	130.1(1)	92.3(1)
**12**	2.816(5)	2.745(5) to 2.905(5)	2.541(5)	O(1) 2.114(4), O(2) 2.109(3)	125.2(2)	91.7(1)
**13**	2.770(13)	2.737(13) to 2.803(12)	2.496(12)	O(1) 2.214(9), S(1) 2.748(3) N(2) 2.530(10)	127.9(3)	61.2(2)^d^
**14**	2.737(5)	2.683(4) to 2.783(5)	2.453(4)	S(1) 2.608(2), I(1) 2.944(1)	130.3(2)	100.2(1)
**15**	2.769(7)	2.722(7) to 2.833(7)	2.489(6)	S(1) 2.755(1), S(2) 2.724(1) N(2) 2.565(5)	131.0(2)	65.1(1)^e^
**17**	2.784(7)	2.684(7) to 2.892(7)	2.506(7)	U(1) Se(1) 2.763(1), Se(2) 2.758(1) U(2) Se(1) 2.761(1), Se(2) 2.758(1)	119.5(2)	79.7(1)

a Cp = cyclopentadienyl ring.

b Average value.

c Range.

d The angle of O(1)-U(1)-S (1).

e The angle of S(1)-U(1)-S(2).

### Bonding Studies

**Figure 7:**
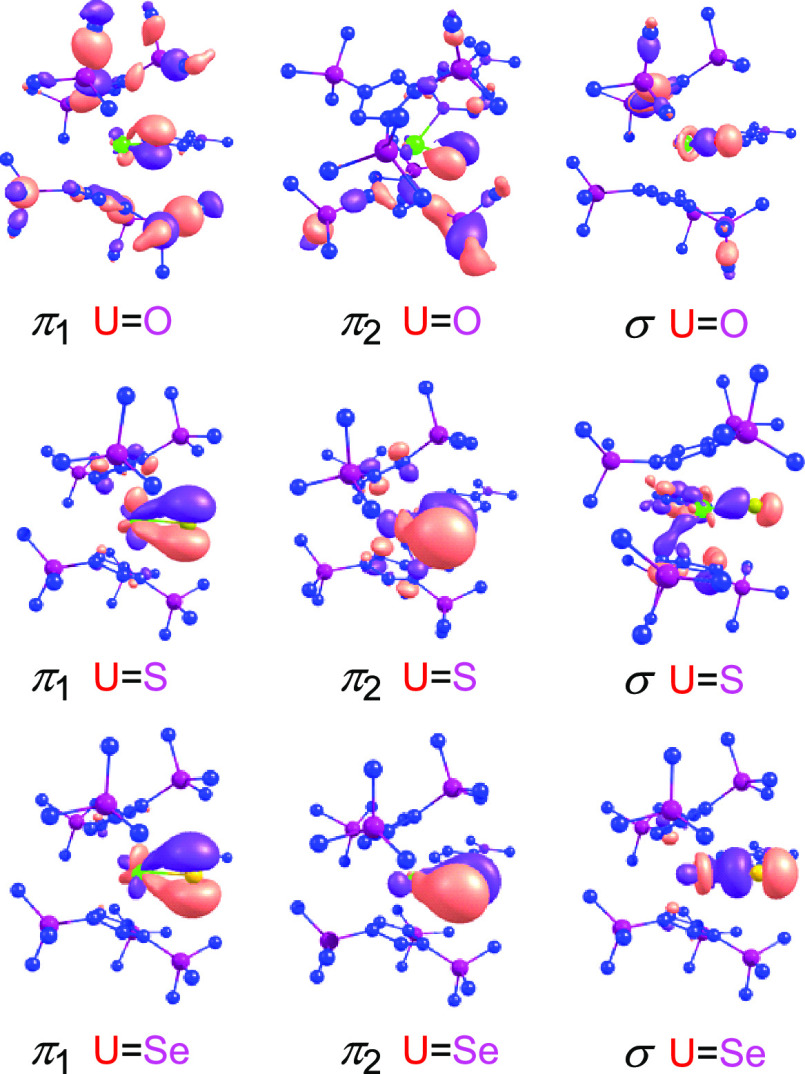
Plots of HOMOs for **5**–**7** (the hydrogen atoms have been omitted for clarity).

To further probe the interaction between the uranium atom and the chalcogenides, oxygen, sulfur, or selenium, density functional theory (DFT) computations at the B3PW91 level of theory were undertaken. While the computed structures of **5–7** are in excellent agreement with the experimental data, computations reveal that the [E]^2–^ fragment is coordinated to the {[η^5^-1,2,4-(Me_3_Si)_3_C_5_H_2_]_2_(dmap)U}^2+^ moiety by one U–E σ-bond and two U–E π-bonds, as illustrated in Figure 7. The bonding in [η^5^-1,2,4-(Me_3_Si)_3_C_5_H_2_]_2_U=E(dmap) was analyzed by a natural localized molecular orbital (NLMO) approach, and the results are summarized in Table 2. The U–E σ-bond comprises a chalcogenide hybrid orbital and a uranium hybrid orbital. Moving from E = O to E = Se, the uranium contribution to the σ-bond increases from 14.1% to 25.7%, while that of the chalcogenide decreases from 85.9% to 74.3%. More importantly, the 6d orbital contribution declines from 69.0% to 59.2%. Notably, the 5f orbital contribution reaches a maximum for E = S (25.8%) but decreases again when moving to E = Se (20.0%). The π bonds (π_1_ and π_2_) are composed of pure chalcogenide-based p orbitals and uranium hybrid orbitals. The U–E π bonds (π_1_ and π_2_) also experience an increased uranium contribution (16.5% to 27.2% for π_1_ and 18.5% to 32.2% for π_2_) when moving from O to Se, while the chalocogenide contribution (83.5% to 72.8% for π_1_ and 81.5% to 67.8% for π_2_) becomes smaller. This is consistent with the increased π interaction between {[η^5^-1,2,4-(Me_3_Si)_3_C_5_H_2_]_2_(dmap)U}^2+^ and [E]^2–^ fragments from O to Se. Differences, however, arise in the contributions of 6d and 5f orbitals to the π_1_ and π_2_ bonds. Whereas the 6d orbital contributions rises slightly (54.2% to 57.4%), the 5f orbital contribution (42.6% to 37.7%) declines for the π_1_ bond. A different trend is, however, observed for the π_2_ bond. Here, the 6d orbital contribution reaches a maximum (38.3%) at E = S, while the 5f orbital contribution is at its minimum (59.9%). Overall, the sum of the 6d and 5f contributions varies only slightly between 98.5% and 98.7%, regardless of the chalogenide. However, the electrostatic charge separation between the two fragments {[η^5^-1,2,4-(Me_3_Si)_3_C_5_H_2_]_2_(dmap)U}^2+^ and [E]^2–^ becomes progressively smaller along the series from O to Se, that is, 1.76 (for E = O (**5**)), 0.84 (for E = S (**6**)), and 0.72 (for E = Se (**7**)) (Table 2), implying that the electrostatic (ionic) interaction between the chalocogenide atom and the uranium atom is reduced from E = O to E = Se. This is correlated to an increase in the Wiberg U=E bond order from 1.80 (for **5**) to 2.24 (for **6**) to 2.25 (for **7**) (Table 2). This also points to an increased orbital overlap and therefore more covalent bonding between the chalocogenide-based valence orbitals and the uranium atom transitioning from E = O to E = Se. These computations indicate a significant involvement of the uranium 5f orbitals in the bonding between the metallocene {[η^5^-1,2,4-(Me_3_Si)_3_C_5_H_2_]_2_(dmap)U}^2+^ and [E]^2–^ fragments, consistent with previous conclusions that the 5f orbitals play a distinct role in the bonding of actinide complexes and that the U=E bonds are generally more polarized than those in d-transition metals.^9c^ This difference carries on in the reactivity of the uranium complexes **5**–**7** when compared to their group 4 relatives.^12,13^

**Table 2:** Natural Localized Molecular Orbital (NLMO) Analysis of U=E Bonds,^a^ Bond Order, and the Natural Charges for the [Cp_2_U(dmap)] and [E] Units

		**5** (O)	**6** (S)	**7** (Se)
σ U-E	%U	14.1	24.1	25.7
	%s	9.5	11.6	15.7
	%p	3.6	3.6	5.1
	%d	69.0	59.0	59.2
	%f	17.9	25.8	20.0
				
	%E	85.9	75.9	74.3
	%s	45.9	48.9	47.6
	%p	54.1	51.0	52.4
				
π_1_ U=E	%U	16.5	26.5	27.2
	%p	3.2	3.5	4.9
	%d	54.2	56.2	57.4
	%f	42.6	40.3	37.7
				
	%E	83.5	73.5	72.8
	%p	100	100	100
				
π_2_ U=E	%U	18.5	29.5	32.2
	%p	1.5	1.8	2.3
	%d	37.3	38.3	35.6
	%f	61.2	59.9	63.1
				
	%E	81.5	70.5	67.8
	%p	100	100	100
				
Wiberg bond order		1.80	2.24	2.25
(U=E)				
NBO charge		0.88	0.42	0.36
(Cp_2_(dmap)U)				
NBO charge (E)		–0.88	–0.42	–0.36

a The contributions by atom and orbital are averaged over all the ligands of the same character and over alpha and beta orbital contributions.

To further assess the U–E bonding, the quantum theory of atoms in molecules (QTAIM) that focuses on the topology of the electron density rather than the orbital structure was adopted to probe the extent of covalency of actinide-ligand bonds, utilizing diagnostic properties such as the electron density (ρ_b_) and energy density (*H*_b_) at the actinide-ligand bond critical points (BCPs) and the delocalization indices (DI) between two atoms.^5d,5j^ ρ_b_ and *H*_b_ data for the U–E at the BCPs and DI data for the U–E bonds in **5**–**7** are collected in Table 3: ρ_b_ values greater than 0.2 eÅ^–3^ are typically associated with covalent bonds and smaller density values are indicative of closed-shell (ionic) interactions;^5d,5j^ the more stabilizing covalent interaction, the more negative energy density *H*_b_,^5d,5j^ whereas the DIs integrate the electron density in the bonding region between two atoms and its value is closely related to the bond order. However, the computed DI values are always smaller than those expected from the Lewis structures and this difference is often attributed to bond polarity.^5j^ According to the QTAIM analysis, the U–E bonds in the complexes **5**–**7** are rather polar and exhibit an appreciable ionic character (Table 3). Moreover, the calculated QTAIM DIs indicate a significant electron density accumulation in the U–E bonding region of the complexes **5**–**7** (Table 3). In addition, the QTAIM analysis suggests that the overlap decreases down the group 16 from O to Se, whereas the energy matching increases (Table 3). Hence, contrary to the NLMO analyses (Table 2), the computed ρ_b_, *H*_b_, and DI values also indicate reduced covalency when moving down in group 16 from O to Se (Table 3), which is also observed for the uranium complexes [{(Me_3_Si)_2_N}_2_U(O)(E)]^−^ (E = O, S, Se).^5j^ This is chemically counterintuitive, but as stated by Kaltsoyannis and coworkers, the QTAIM parameters evaluated at the BCPs have to be treated with caution,^5c,14^ when a series with different bond distances are compared. The U–E distances (computed U–E distance of 1.847, 2.422, and 2.573 Å for **5**, **6**, and **7**, respectively) vary significantly and therefore caution should be exercised in concluding that the U–E covalence for complexes **5**–**7** decreases as group 16 is descended.

**Table 3:** Electron Densities (ρ_b_) and Energy Densities (*H*_b_) at Selected Bond Critical Points of 5–7 (*H* in Bold, Both in Atomic Units) and Delocalization Indices (DI) for U=E Bonds

	**5** (O)	**6** (S)	**7** (Se)
electron densities (ρ)	0.122	0.089	0.018
energy densities (*H*)	–0.118	–0.019	–0.010
delocalization indices (DI)	2.470	2.312	2.286

### Reactivity of Oxido Complex [η^5^-1,2,4-(Me_3_Si)_3_C_5_H_2_]_2_UO(dmap) (**5**)

**Figure 8:**
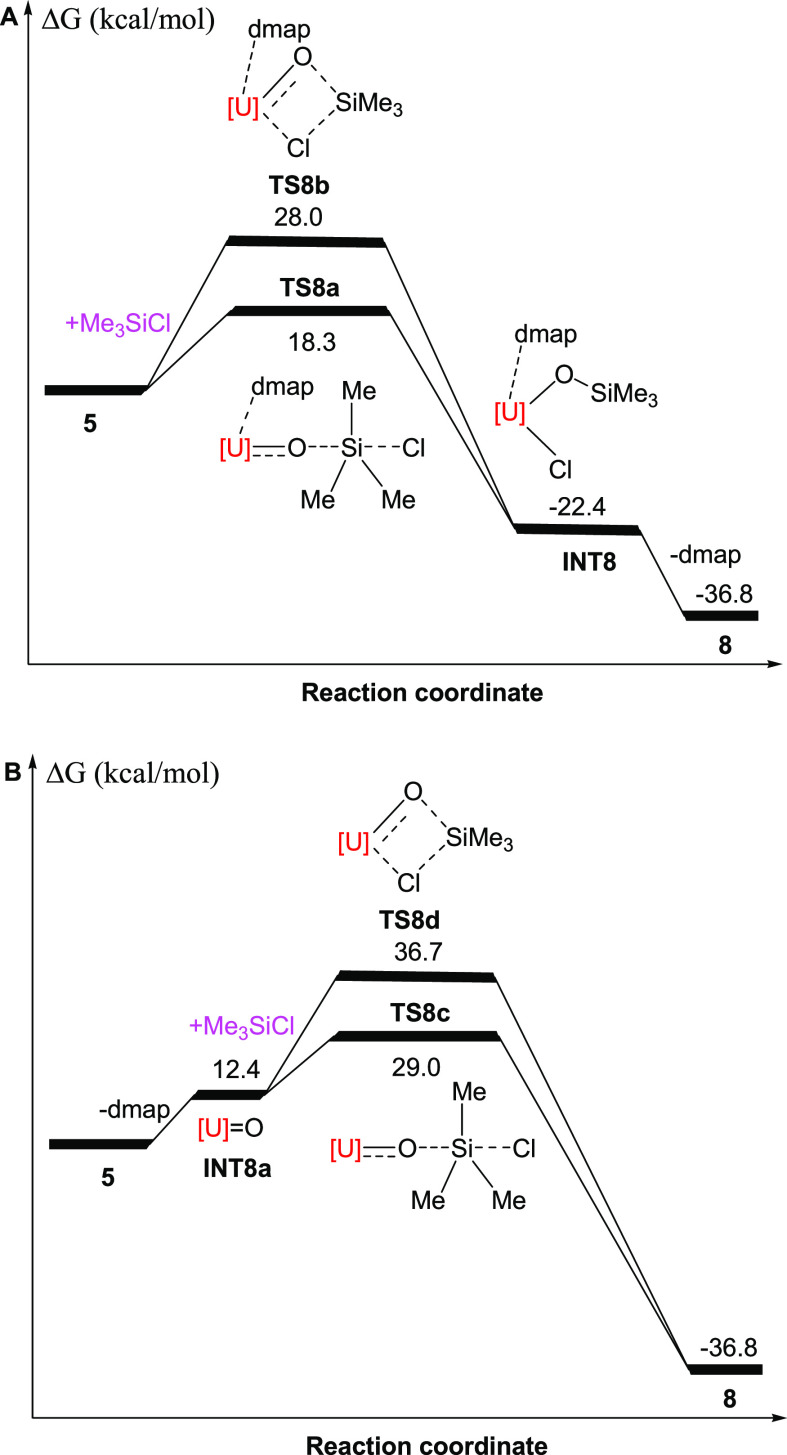
Energy profile (kcal/mol) for the reaction of **5** + Me_3_SiCl (computed at *T* = 298 K). [U]=[η^5^-1,2,4-(Me_3_Si)_3_C_5_H_2_]_2_U.

**Figure 9:**
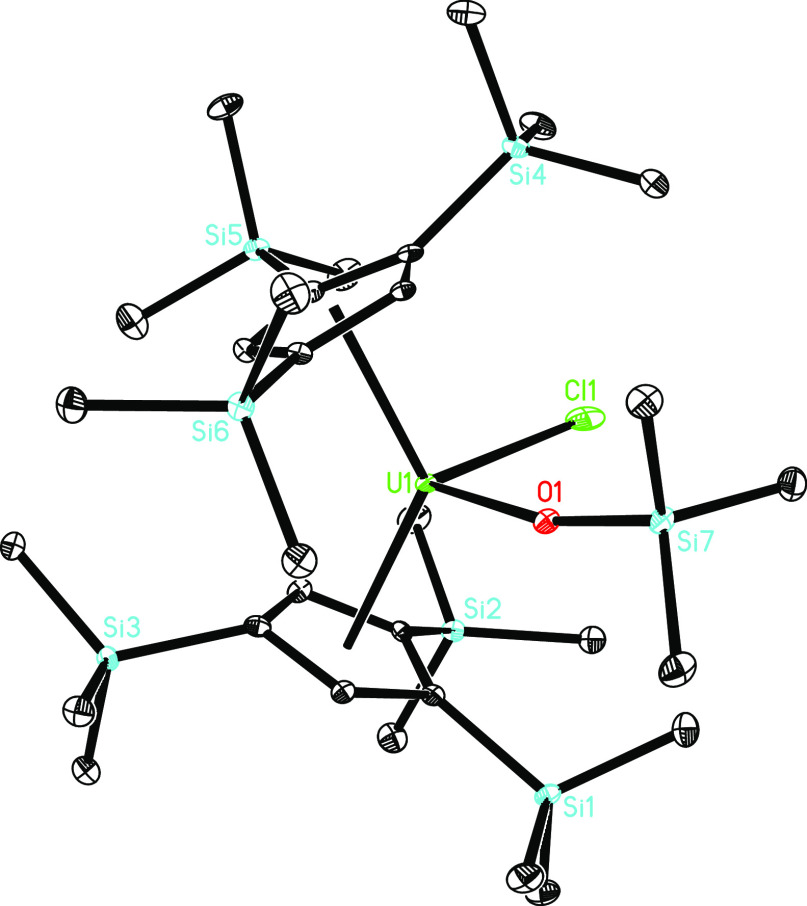
Molecular structure of **8** (thermal ellipsoids drawn at the 35% probability level).

**Figure 10:**
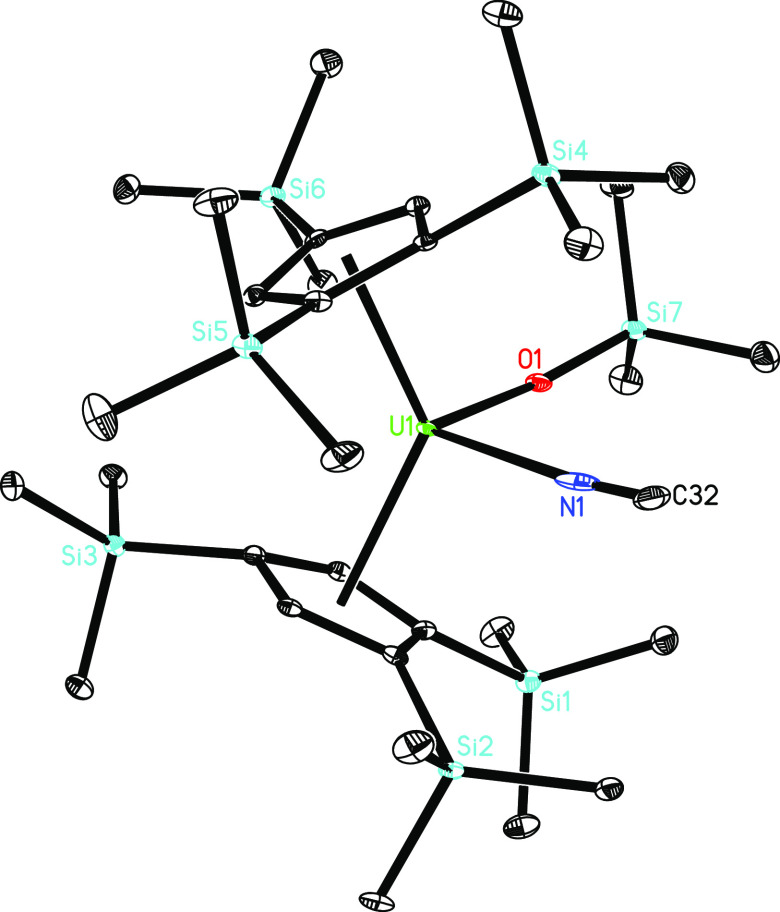
Molecular structure of **10** (thermal ellipsoids drawn at the 35% probability level).

**Figure 11:**
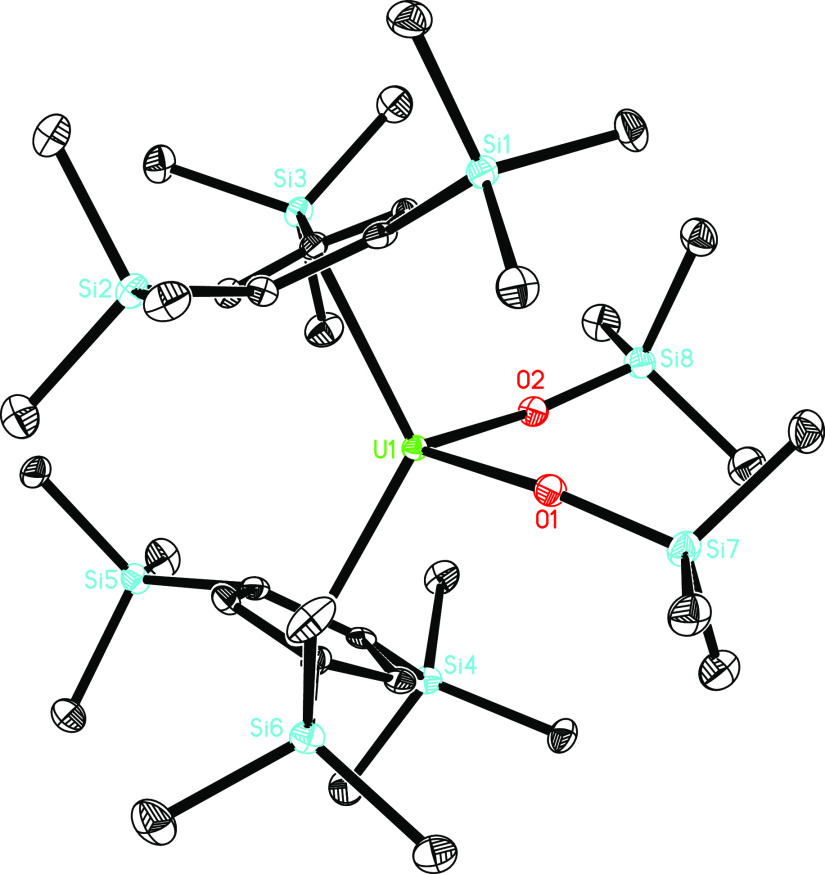
Molecular structure of **12** (thermal ellipsoids drawn at the 35% probability level).

**Figure 12:**
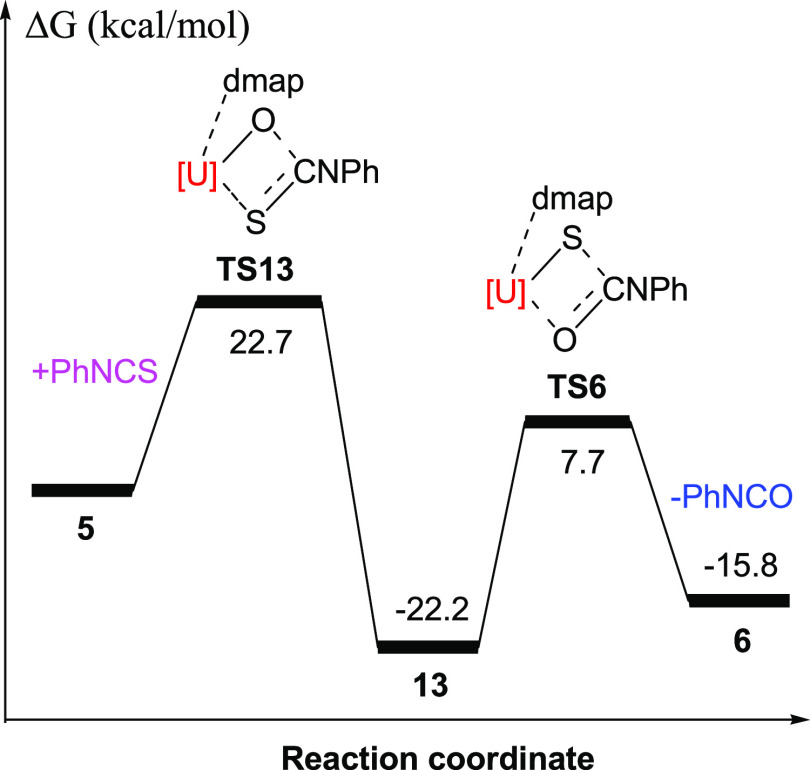
Energy profile (kcal/mol) for the reaction of **5** + PhNCS (computed at *T* = 298 K). [U]=[η^5^-1,2,4-(Me_3_Si)_3_C_5_H_2_]_2_U.

**Figure 13:**
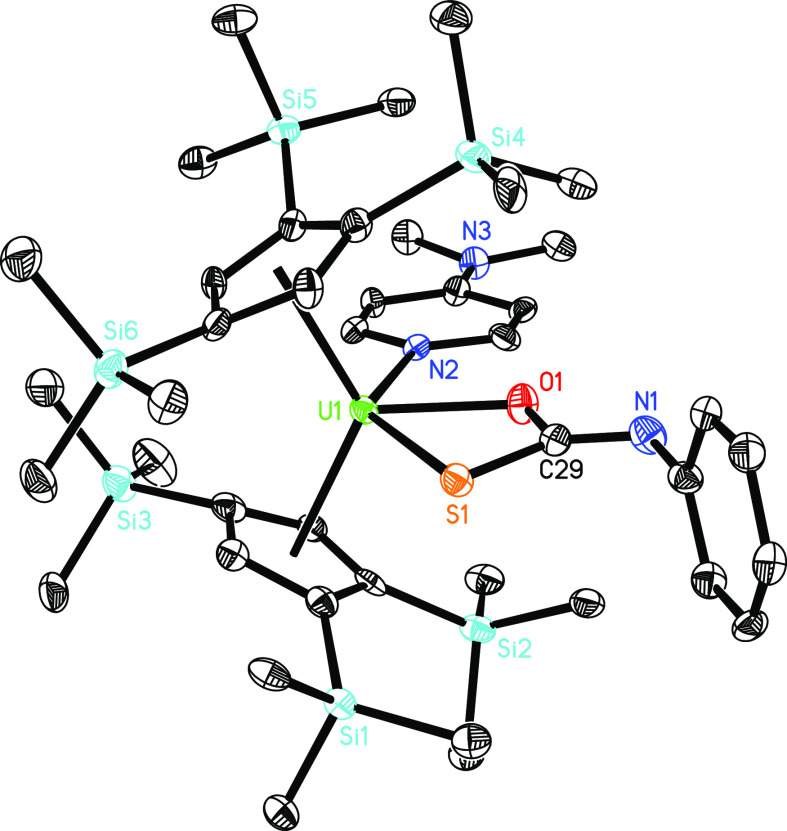
Molecular structure of **13** (thermal ellipsoids drawn at the 35% probability level).

**Scheme 2:**
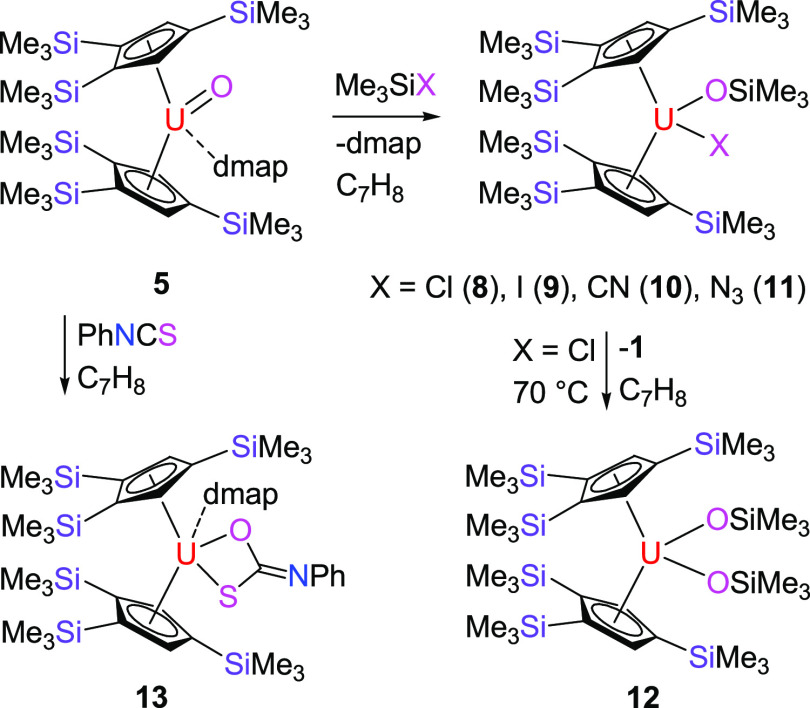
Synthesis of Complexes **8**–**13**

**Scheme 3:**
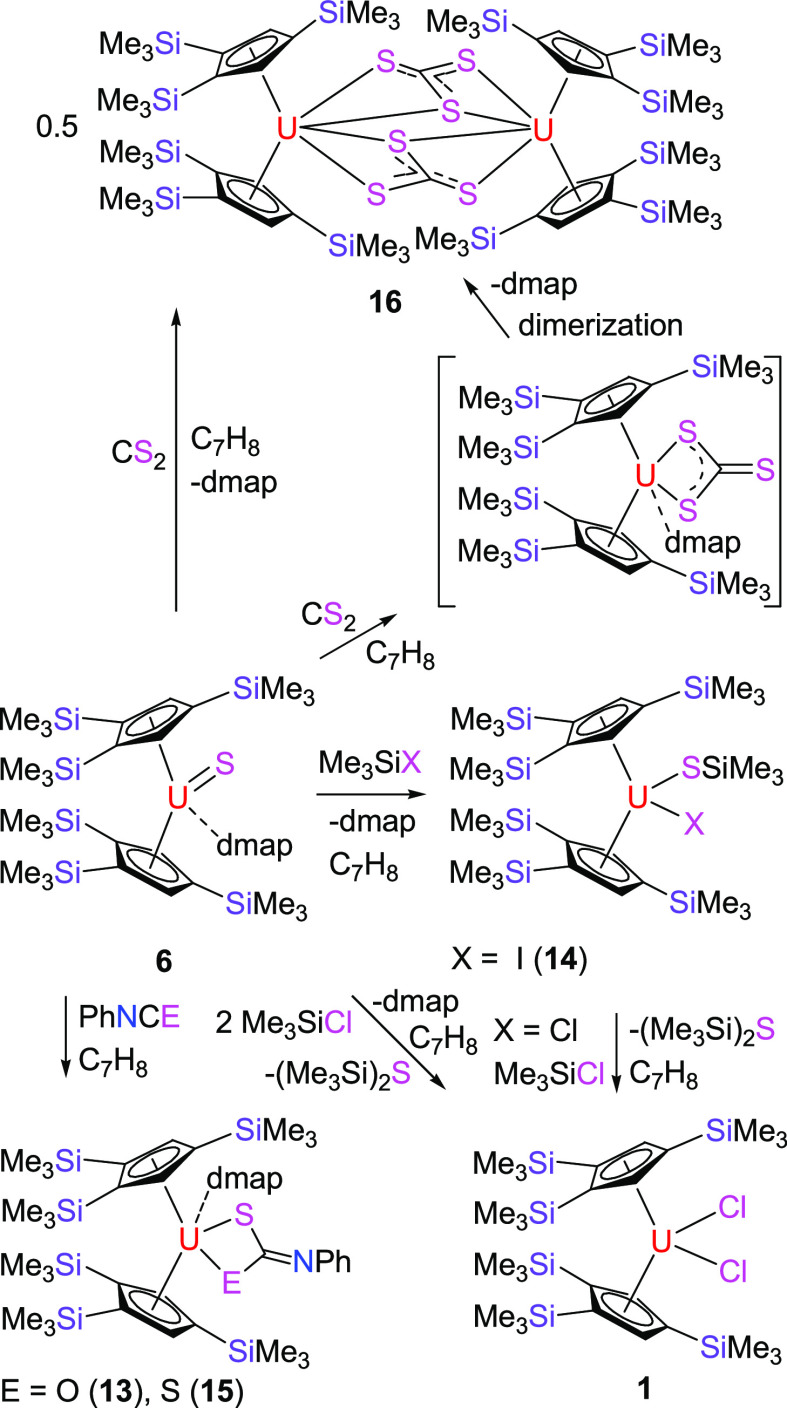
Synthesis of Complexes **13**–**16**

The more polarized nature of the moiety U=O may also impact its reactivity. Nevertheless, in contrast to [η^5^-1,2,4-(Me_3_C)_3_C_5_H_2_]_2_U=O(dmap),^3e^ the dmap ligand in [η^5^-1,2,4-(Me_3_Si)_3_C_5_H_2_]_2_U=O(dmap) (**5**) cannot be removed by Ph_3_B addition to yield a base free oxido complex [η^5^-1,2,4-(Me_3_Si)_3_C_5_H_2_]_2_UO, presumably due to the more electron-deficient and less sterically demanding ligand 1,2,4-(Me_3_Si)_3_C_5_H_2_, which renders the uranium atom more electrophilic and renders a more open coordination sphere at the uranium atom, therefore binding the dmap more strongly. Nevertheless, in analogy to the oxido compounds [η^5^-1,2,4-(Me_3_C)_3_C_5_H_2_]_2_An=O(dmap) (An = Th, U),^3e,10b^ [η^5^-1,2,4-(Me_3_Si)_3_C_5_H_2_]_2_U=O(dmap) (**5**) forms with Me_3_SiX the metallocenes, [η^5^-1,2,4-(Me_3_Si)_3_C_5_H_2_]_2_U(OSiMe_3_)(X) (X = Cl (**8**), I (**9**), NC (**10**), and N_3_ (**11**)), concomitant with dmap loss (Scheme 2). DFT studies suggest that the reaction of **5** with Me_3_SiCl may proceed *via* two different ways, *i.e.*, an S_N_2 (**TS8a**) or an addition (**TS8b**) mechanism (Figure 8A). The optimized bond distances of O–Si and Si–Cl in **TS8a** are 2.479 and 2.245 Å (see the Supporting Information for details), respectively, indicating that the O–Si bond is formed, while the Si–Cl bond is broken simultaneously, and the resulting Cl^–^ anion migrates to the U atom to establish a U–Cl bond when it leaves the Si atom. The Si atom in Me_3_SiCl can readily approach the O atom, and the energy barrier for the S_N_2 process is only Δ*G*^‡^(298 K) = 18.3 kcal/mol (Figure 8A), implying that the reaction readily proceeds at ambient temperature. In contrast, the addition reaction occurs *via* the concerted transition state **TS8b**, in which the two forming bond distances of U–Cl and Si–O are 4.254 and 2.263 Å, respectively (see the Supporting Information for details). In combination with the Si–Cl distance of 2.167 Å, these metric parameters imply that the O–Si and U–Cl bond formations and Si–Cl bond breakage occur simultaneously, in which the formation of the Si–O bond drives the Si–Cl bond breakage. Nevertheless, the activation barrier of Δ*G*^‡^(298 K) = 28.0 kcal/mol for the addition reaction of **5** with Me_3_SiCl (Figure 8A) is energetically less favorable than that of the S_N_2 reaction. However, the formation of **INT8** is thermodynamically energetically favorable (Δ*G*(298 K) = −22.4 kcal/mol), but driven by dmap loss, **INT8** immediately converts to the even more thermodynamically stable product **8** (Δ*G*(298 K) = −36.8 kcal/mol) (Figure 8A). Moreover, it is reasonable to assume that the U–Cl and Si–S bond formation energies in the reaction of [η^5^-1,2,4-(Me_3_Si)_3_C_5_H_2_]_2_US(dmap) (**6**) with Me_3_SiCl favor the formation of [η^5^-1,2,4-(Me_3_Si)_3_C_5_H_2_]_2_UCl_2_ and (Me_3_Si)_2_S (Scheme 3), whereas the reaction of **5** with Me_3_SiCl stops with the formation of [η^5^-1,2,4-(Me_3_Si)_3_C_5_H_2_]_2_U(OSiMe_3_)Cl (**8**) attributed to the strong U–O bond (Scheme 2). We also considered that dmap dissociation initiates the formation of **8***via* either an S_N_2 (**TS8c**) or an addition (**TS8d**) reaction with Me_3_SiCl. However, the activation barriers for these processes of Δ*G*^‡^(298 K) = 29.0 and = 36.7 kcal/mol (Figure 8B), respectively, are significantly larger than that for the S_N_2 reaction illustrated in Figure 8A, whose computed reaction profile is in agreement with **8** being formed at ambient temperature. The molecular structures of **8** and **10** are presented in Figures 9 and 10, respectively, and ORTEP of **9** is shown in the Supporting Information. In complex **8**, the U–O distance of 2.071(3) Å is close to that in **9** (2.077(3) Å). The U–Cl distance in **8** is 2.820(1) Å, whereas the U–I distance amounts to 2.963(1) Å in **9**. In contrast to uranium cyanide complex [η^5^-1,2,4-(Me_3_C)_3_C_5_H_2_]_2_U(OSiMe_3_)(CN)^3e^ but analogous to uranium isocyanide complex [(Me_3_Si)_2_N]_3_U(NC) and the thorium isocyanide complex [η^5^-1,2,4-(Me_3_C)_3_C_5_H_2_]_2_Th(OSiMe_3_)(NC),^10b,15^ the CN^–^ ligand in **10** coordinates to the U^4+^ ion by its nitrogen atom instead of the carbon atom, presumably due to the electron-deficient nature of 1,2,4-(Me_3_Si)_3_C_5_H_2_, which increases the Lewis acidity of the metal ion. DFT computations also predict the isocyanide isomer to be energetically more favorable than the cyanide one [η^5^-1,2,4-(Me_3_Si)_3_C_5_H_2_]_2_U(OSiMe_3_)(CN) (Δ*G*(298 K) = −0.8 kcal/mol) (see the Supporting Information for details), which is in line with the experiment. The U–O distance of 2.067(2) Å is shorter than those in **8** and **9** (Table 1), whereas the U–N distance is 2.525(4) Å. Nevertheless, complexes **8**–**10** are unstable toward ligand redistribution at high temperature in toluene solution. For example, when heated at 70 °C, **8** yields **1** and [η^5^-1,2,4-(Me_3_Si)_3_C_5_H_2_]_2_U(OSiMe_3_)_2_ (**12**) (Scheme 2). The molecular structure of **12** is provided in Figure 11; for selected bond distances and angles, consult Table 1. The U–O distances are 2.114(4) and 2.109(3) Å, and the angle of O–U–O is 91.7(1)°. Moreover, in analogy to the thorium oxido [η^5^-1,2,4-(Me_3_C)_3_C_5_H_2_]_2_Th=O(dmap),^10b^ [η^5^-1,2,4-(Me_3_Si)_3_C_5_H_2_]_2_U=O(dmap) (**5**) is reactive toward unsaturated organic substrates. For example, treatment of **5** with PhNCS in toluene rapidly forms the four-membered metallocene [η^5^-1,2,4-(Me_3_Si)_3_C_5_H_2_]_2_U[OC(=NPh)S)(dmap) (**13**) (Scheme 2). DFT investigations imply that the formation of **13** from **5** + PhNCS is exergonic with Δ*G*(298 K) = −22.2 kcal/mol and proceeds *via* a transition state **TS13** with a low reaction barrier of Δ*G*^‡^(298 K) = +22.7 kcal/mol (Figure 12). Moreover, DFT computations also predict that the degradation of **13** to **6** + PhNCO is energetically unfavorable (Δ*G*(298 K) = 6.4 kcal/mol) and proceeds *via* a transition state **TS6** with a high reaction barrier of Δ*G*^‡^(298 K) = +29.9 kcal/mol (Figure 12). These results are consistent with the experiment in which complex **13** is formed at ambient temperature. The molecular structure of **13** is shown in Figure 13; for selected bond distances and angles, consult Table 1. The U–N distance of 2.530(10) Å is in line with those found in **4**–**7** (Table 1). The U–O distance is 2.214(9) Å, whereas the U–S distance of 2.748(3) Å is longer than that found in **6** (2.437(1) Å). However, like for the actinide oxidos [η^5^-1,2,4-(Me_3_C)_3_C_5_H_2_]_2_An=O(dmap) (An = Th, U)^3e,10b^ but in contrast to the group 4 derivatives (η^5^-C_5_Me_5_)_2_Ti=O(py) and (η^5^-C_5_Me_5_)_2_Zr=O,^12b,12c,13^ no reaction occurs between complex **5** and internal alkynes RC≡CR (R = Ph, Me, *p*-tolyl) even when heated to 100 °C for 1 week, presumably attributed to the more polarized nature of the actinide oxido bond An^+^-O^–^.^16^

### Reactivity of Sulfido Complex [η^5^-1,2,4-(Me_3_Si)_3_C_5_H_2_]_2_U=S(dmap) (**6**)

**Figure 14:**
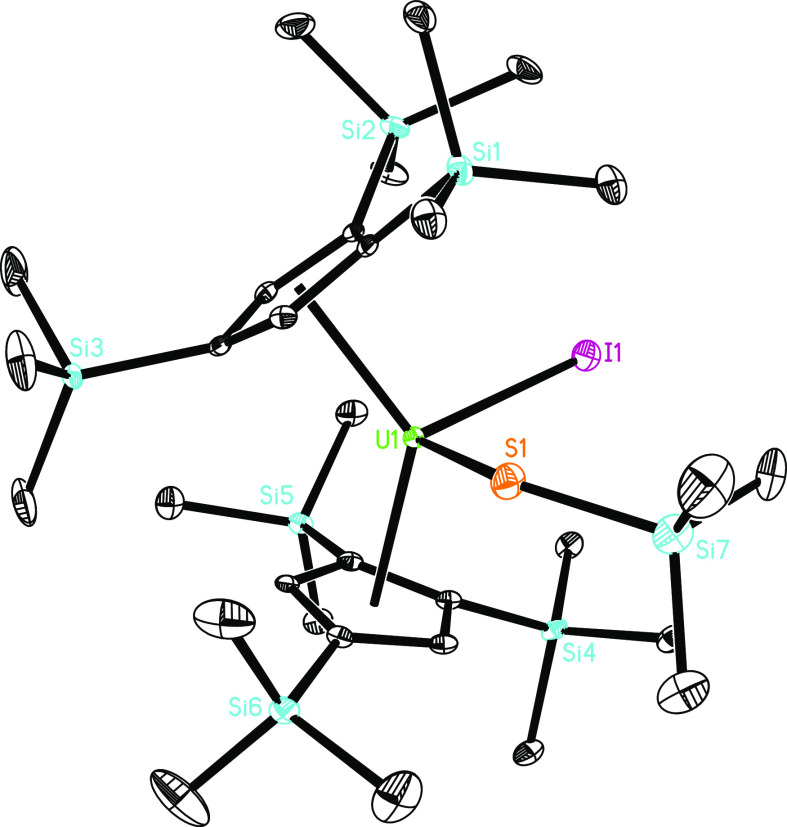
Molecular structure of **14** (thermal ellipsoids drawn at the 35% probability level).

**Figure 15:**
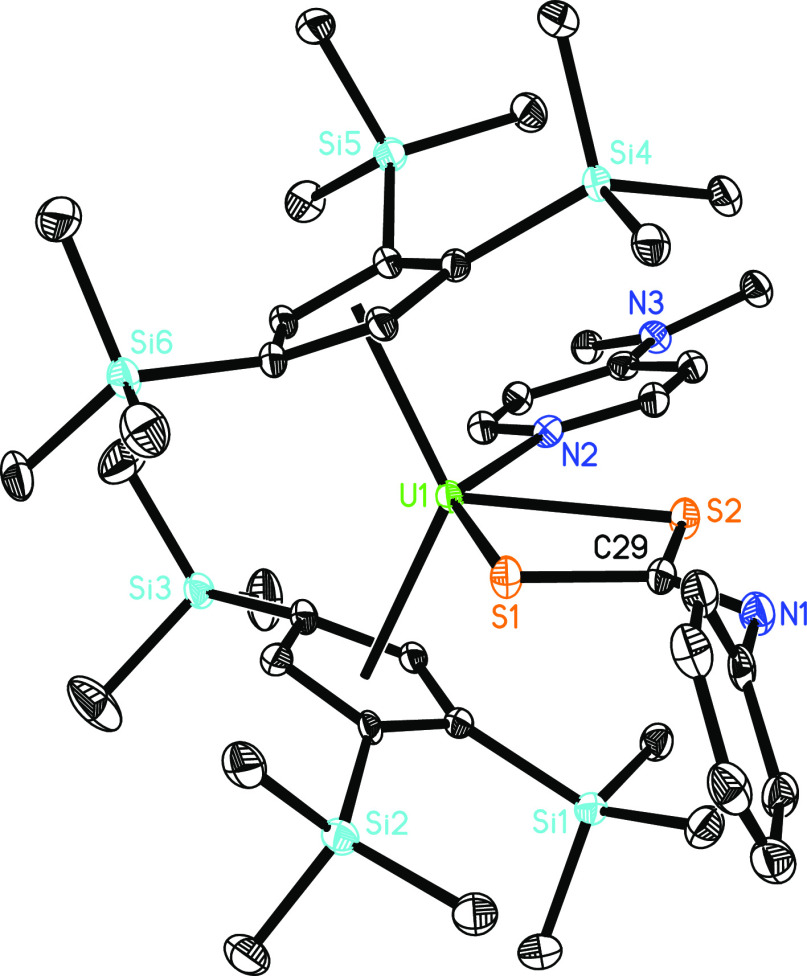
Molecular structure of **15** (thermal ellipsoids drawn at the 35% probability level).

In contrast to the uranium sulfido complex [η^5^-1,2,4-(Me_3_C)_3_C_5_H_2_]_2_U=S that readily reacts with one equivalent of Ph_2_CS to form [η^5^-1,2,4-(Me_3_C)_3_C_5_H_2_]_2_U(S_2_CPh_2_) even in the presence of a Lewis base such as dmap,^11^ the dmap-stabilized terminal uranium sulfido [η^5^-1,2,4-(Me_3_Si)_3_C_5_H_2_]_2_U=S(dmap) (**6**) is formed from the reaction of [η^5^-1,2,4-(Me_3_Si)_3_C_5_H_2_]_2_U=N(*p*-tolyl)(dmap) (**4**) and Ph_2_CS at room temperature (Scheme 1). We attribute this difference to a combination of steric and electronic effects. The 1,2,4-(Me_3_Si)_3_C_5_H_2_ is less bulky and therefore accommodates dmap coordination, but it is also more electron-deficient and hence increases the Lewis acidity of the uranium ion. In analogy to its oxido counterparts [η^5^-1,2,4-(Me_3_E)_3_C_5_H_2_]_2_U=O(dmap) (E = C,^3e^ Si (**5**)), at room temperature, complex **6** reacts immediately upon mixing with Me_3_SiI to release the dmap ligand and to give the addition product [η^5^-1,2,4-(Me_3_Si)_3_C_5_H_2_]_2_U(SSiMe_3_)(I) (**14**) (Scheme 3). The molecular structure of **14** is depicted in Figure 14; for selected bond distances and angles, see Table 1. The U–I distance of 2.944(1) Å is comparable to that found in **9** (2.963(1) Å), whereas the U–S distance of 2.608(2) Å is longer than that found in **6** (2.437(1) Å). Nevertheless, when Me_3_SiCl is used as a substrate, dichlorido complex [η^5^-1,2,4-(Me_3_Si)_3_C_5_H_2_]_2_UCl_2_ (**1**) is formed along with dmap and (Me_3_Si)_2_S loss (Scheme 3). Most likely, this reaction proceeds *via* a monochlorido intermediate [η^5^-1,2,4-(Me_3_Si)_3_C_5_H_2_]_2_U(SSiMe_3_)(Cl) but it readily converts in the presence of Me_3_SiCl *via* (Me_3_Si)_2_S loss to the product **1** (Scheme 3). This contrasts to the reactivity of **5** with Me_3_SiCl yielding as a stable product [η^5^-1,2,4-(Me_3_Si)_3_C_5_H_2_]_2_U(OSiMe_3_)(Cl) (**8**) (Scheme 2). Moreover, like its oxido counterpart [η^5^-1,2,4-(Me_3_Si)_3_C_5_H_2_]_2_U=O(dmap) (**5**), **6** reacts with unsaturated organic substrates. For example, treatment of **6** with PhNCO or PhNCS in toluene forms the four-membered metallocenes, [η^5^-1,2,4-(Me_3_Si)_3_C_5_H_2_]_2_U[EC(=NPh)S)(dmap) (E = O (**13**), S (**15**)) (Scheme 3). The formation of **13** from **6** + PhNCO is consistent with the reaction energy profile in Figure 12. The molecular structure of **15** is provided in Figure 15; for selected bond distances and angles, refer to Table 1. The U–N distance is 2.565(5) Å, whereas the U–S distances are 2.755(1) and 2.724(1) Å. Moreover, complex **6** transforms in the presence of CS_2_ to a uranium trithiocarbonato intermediate [η^5^-1,2,4-(Me_3_Si)_3_C_5_H_2_]_2_U(CS_3_)(dmap), which readily dimerizes to the trithiocarbonato-bridged complex {[η^5^-1,2,4-(Me_3_Si)_3_C_5_H_2_]_2_U}_2_(μ-CS_3_)_2_ (**16**)^17^ concomitant with dmap release (Scheme 3). However, in analogy to the thorium sulfido [η^5^-1,2,4-(Me_3_C)_3_C_5_H_2_]_2_Th=S^10b^ but in contrast to the group 4 derivatives (η^5^-C_5_Me_5_)_2_TiS(py)^12f^ and (η^5^-C_5_Me_5_)_2_ZrS,^12b,13^ no [2 + 2] cycloaddition products [η^5^-1,2,4-(Me_3_Si)_3_C_5_H_2_]_2_U[SC(R)=C(R)] are obtained between complex **6** and internal alkynes RC≡CR (R = Ph, Me, *p*-tolyl) even when the mixture is heated at 100 °C for 1 week, again, presumably due to the more polarized nature of the actinide sulfido bond An^+^–S^–^.^16^

### Reactivity of Selenido Complex [η^5^-1,2,4-(Me_3_Si)_3_C_5_H_2_]_2_U=Se(dmap) (7)

**Figure 16:**
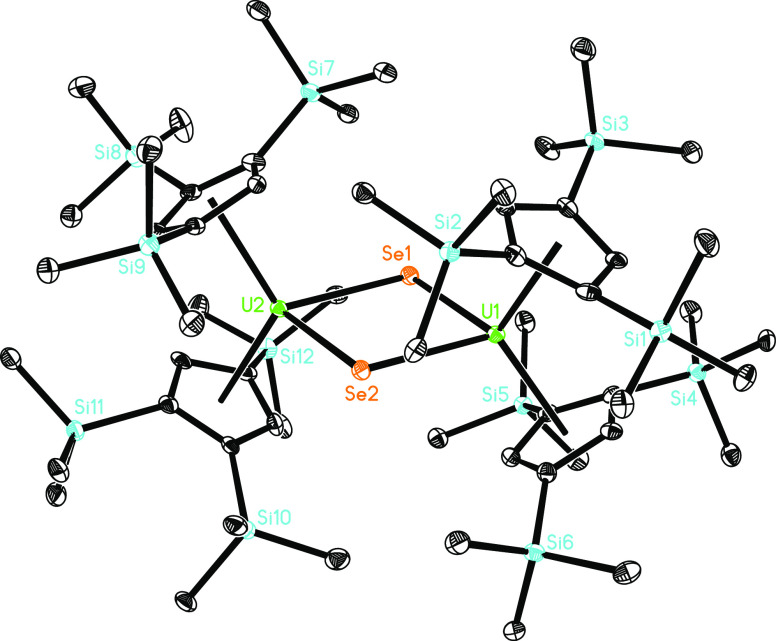
Molecular structure of **17** (thermal ellipsoids drawn at the 35% probability level).

**Scheme 4:**
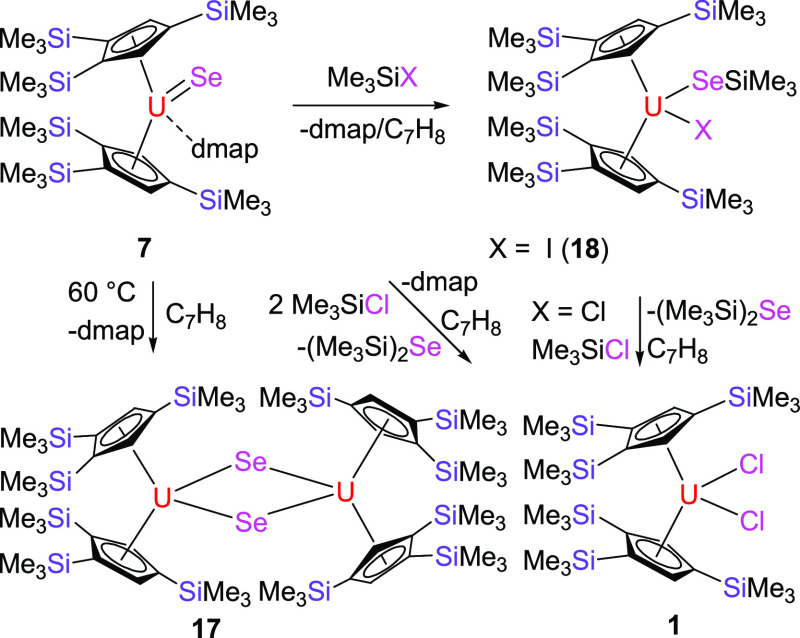
Synthesis of Complexes **17** and **18**

In contrast to the oxido and sulfido derivatives, [η^5^-1,2,4-(Me_3_Si)_3_C_5_H_2_]_2_U=O(dmap) (**5**) and [η^5^-1,2,4-(Me_3_Si)_3_C_5_H_2_]_2_U=S(dmap) (**6**), the isolated selenido adduct [η^5^-1,2,4-(Me_3_Si)_3_C_5_H_2_]_2_U=Se(dmap) (**7**) degrades in toluene solution at 60 °C to the dimer {[η^5^-1,2,4-(Me_3_Si)_3_C_5_H_2_]_2_U}_2_(μ-Se)_2_ (**17**) (Scheme 4), presumably due to the larger ionic radius of the Se^2–^ anion. The molecular structure of **17** is provided in Figure 16; for selected bond distances and angles, see Table 1. The U–Se distances within the U_2_Se_2_-core are essentially identical and vary between 2.758(1) and 2.763(1) Å. This also extends to the Se–U(1)–Se angles and the Se–U(2)–Se angles; both are 79.7(1)°. Like the oxido [η^5^-1,2,4-(Me_3_Si)_3_C_5_H_2_]_2_U=O(dmap) (**5**) and sulfido [η^5^-1,2,4-(Me_3_Si)_3_C_5_H_2_]_2_U=S(dmap) (**6**), complex **7** immediately reacts upon mixing with Me_3_SiI at room temperature to give the addition product [η^5^-1,2,4-(Me_3_Si)_3_C_5_H_2_]_2_U(SeSiMe_3_)(I) (**18**) concomitant with dmap loss (Scheme 4). Moreover, analogous to the sulfido derivative [η^5^-1,2,4-(Me_3_Si)_3_C_5_H_2_]_2_U=S(dmap) (**6**), when Me_3_SiCl is used as a substrate, the dichlorido complex [η^5^-1,2,4-(Me_3_Si)_3_C_5_H_2_]_2_UCl_2_ (**1**) is formed along with dmap and (Me_3_Si)_2_Se (Scheme 4). Again, this reaction proceeds *via* a monochlorido intermediate [η^5^-1,2,4-(Me_3_Si)_3_C_5_H_2_]_2_U(SeSiMe_3_)(Cl), which readily converts in the presence of Me_3_SiCl releasing (Me_3_Si)_2_Se to give complex **1** (Scheme 4). However, unlike the oxido [η^5^-1,2,4-(Me_3_Si)_3_C_5_H_2_]_2_U=O(dmap) (**5**) and sulfido [η^5^-1,2,4-(Me_3_Si)_3_C_5_H_2_]_2_U=S(dmap) (**6**), complex **7** shows no reaction with PhNCS, presumably attributed to the large size of the Se^2–^ anion, which hinders the approach to the uranium atom. Moreover, like the oxido [η^5^-1,2,4-(Me_3_Si)_3_C_5_H_2_]_2_U=O(dmap) (**5**) and sulfido [η^5^-1,2,4-(Me_3_Si)_3_C_5_H_2_]_2_U=S(dmap) (**6**), also in the case of **7**, no reaction is observed in the presence of internal alkynes RC≡CR (R = Ph, Me, *p*-tolyl).^.^

## Conclusions

The uranium oxido metallocenes [η^5^-1,2,4-(Me_3_E)_3_C_5_H_2_]_2_U=O(dmap) (E = C,^3e^ Si (**5**)) and the thorium derivative [η^5^-1,2,4-(Me_3_C)_3_C_5_H_2_]_2_Th=O(dmap)^10b^ react as nucleophiles toward alkylsilyl halides mimicking the reactivity of (η^5^-C_5_Me_5_)_2_Zr=O(py),^12e^ but they do not undergo cycloaddition reactions with alkynes in contrast to (η^5^-C_5_Me_5_)_2_Ti=O(py)^12c^ and (η^5^-C_5_Me_5_)_2_Zr=O.^12b^ Moreover, both the uranium sulfido [η^5^-1,2,4-(Me_3_Si)_3_C_5_H_2_]_2_U=S(dmap) (**6**) and its thorium relative [η^5^-1,2,4-(Me_3_C)_3_C_5_H_2_]_2_Th=S are inert toward internal alkynes in contrast to (η^5^-C_5_Me_5_)_2_Ti=S(py)^12f^ and (η^5^-C_5_Me_5_)_2_Zr=S, which participate in cycloaddition reactions.^12b^ We propose that these differences arise from the more polarized nature of the actinide oxido and sulfido bonds An^+^–E^–^ (E = O, S).^9c^

Moreover, uranium sulfido and selenido metallocenes exhibit distinctly different reactivity patterns, e.g., whereas the sulfido derivative [η^5^-1,2,4-(Me_3_Si)_3_C_5_H_2_]_2_U=S(dmap) (**6**) is stable in toluene solution, its selenido counterpart [η^5^-1,2,4-(Me_3_Si)_3_C_5_H_2_]_2_U=Se(dmap) (**7**) degrades to the dimer {[η^5^-1,2,4-(Me_3_Si)_3_C_5_H_2_]_2_U}_2_(μ-Se)_2_ (**17**). Furthermore, while **6** undergoes a cycloaddition with PhNCS, **7** remains inert toward PhNCS. This shows that the reactivity of actinide metallocenes carrying a terminal An=E (E = heteroatom) functional group is influenced by the size of the heteroatom as well as the polarization of the An=X bond.

Furthermore, this study also illustrates that small variations in the supporting cyclopentadienyl ligand modulate the reactivity of these uranium metallocenes. For example, the dmap ligand in complex [η^5^-1,2,4-(Me_3_C)_3_C_5_H_2_]_2_U=O(dmap) can be readily removed by Ph_3_B,^3e^ whereas this is not possible for [η^5^-1,2,4-(Me_3_Si)_3_C_5_H_2_]_2_U=O(dmap) (**5**). Moreover, in contrast to the uranium cyanide complex [η^5^-1,2,4-(Me_3_C)_3_C_5_H_2_]_2_U(OSiMe_3_)(CN),^3e^ the uranium isocyanide complex [η^5^-1,2,4-(Me_3_Si)_3_C_5_H_2_]_2_U(OSiMe_3_)(NC) (**10**) is energetically more favorable than its cyanide isomer [η^5^-1,2,4-(Me_3_Si)_3_C_5_H_2_]_2_U(OSiMe_3_)(CN). Furthermore, while [η^5^-1,2,4-(Me_3_Si)_3_C_5_H_2_]_2_U=S(dmap) (**6**) is stable, the complex [η^5^-1,2,4-(Me_3_C)_3_C_5_H_2_]_2_U=S(dmap) is not.^11^ Electronic effects of the cyclopentadienyl ligands might also influence the reactivity of these complexes, whereas steric effects seem to prevail in all of these systems, dictating the reaction products being formed. Since the ligand 1,2,4-(Me_3_Si)_3_C_5_H_2_ is less sterically demanding than 1,2,4-(Me_3_C)_3_C_5_H_2_, this renders the open wedge at the uranium atom more open, inducing smaller Cp(cent)-U-Cp(cent) angles, which allows additional stabilizing ligands to coordinate, which enables the isolation of reaction products and intermediates inaccessible to uranium fragments bearing the more sterically encumbered 1,2,4-(Me_3_C)_3_C_5_H_2_ derivative.

In conclusion, the imido uranium metallocene [η^5^-1,2,4-(Me_3_Si)_3_C_5_H_2_]_2_U=N(*p*-tolyl)(dmap) (**4**) is a useful precursor for the synthesis of the terminal oxido, sulfido, and selenido uranium metallocenes, which enabled us to systematically probe the intrinsic reactivity of U=E (E = O, S, Se) moieties. This allowed us to map the space of chemical transformations accessible to organoactinide oxido and chalcogenido complexes, which may also extend to solid-state actinide metal oxides and chalcogenides. Further investigations on the intrinsic reactivity of terminal chalcogenido actinide metallocenes and the uranium imido metallocene **4** are ongoing and will be communicated in due course.

## Experimental Section

### General Procedures

All reactions and product manipulations were carried out under an atmosphere of dry dinitrogen with rigid exclusion of air and moisture using standard Schlenk or cannula techniques, or in a glove box. All organic solvents were freshly distilled from sodium benzophenone ketyl immediately prior to use. [η^5^-1,2,4-(Me_3_Si)_3_C_5_H_2_]_2_UCl_2_ (**1**)^17^ and (*p*-MeOPh)_2_CSe^18^ were prepared according to previously reported procedures. All other chemicals were purchased from Aldrich Chemical Co. and Beijing Chemical Co. and used as received unless otherwise noted. Infrared spectra were recorded in KBr pellets on an Avatar 360 Fourier transform spectrometer. ^1^H and ^13^C{^1^H} NMR spectra were recorded on a Bruker AV 400 spectrometer at 400 and 100 MHz, respectively. ^29^Si{^1^H} NMR spectra were recorded on a JEOL 600 spectrometer at 119.2 MHz. All chemical shifts are reported in δ units with reference to the residual protons of the deuterated solvents, which served as internal standards, for proton and carbon chemical shifts, and to external Me_4_Si (0.00 ppm) for silicon chemical shifts. Melting points were obtained on X-6 melting point apparatus and were uncorrected. Elemental analyses were performed on a Vario EL elemental analyzer.

### Preparation of [η^5^**-**1,2,4-(Me_3_Si)_3_C_5_H_2_]_2_UMe_2_ (**2**)

A diethyl ether (30.7 mL) solution of MeLi (0.15 M in diethyl ether; 4.6 mmol) was slowly added to a diethyl ether (25 mL) solution of [η^5^-1,2,4-(Me_3_Si)_3_C_5_H_2_]_2_UCl_2_ (**1**; 2.00 g, 2.3 mmol) with stirring at room temperature. After the solution was stirred for 1 h at room temperature, the solvent was removed. The residue was extracted with *n*-hexane (15 mL × 3) and filtered. The volume of the filtrate was reduced to 10 mL, and orange microcrystals of **2** formed when this solution was kept at −20 °C for 2 days. Microcrystals of **2** were isolated by filtration, quickly washed with cooled *n*-hexane (5 mL), and dried at 50 °C under vacuum overnight. Yield: 1.57 g (82%). M.p.: 150–152 °C (dec.). ^1^H NMR (C_6_D_6_): δ −0.07 (36H, Si(C*H*_3_)_3_), −2.32 (18H, Si(C*H*_3_)_3_), −52.98 (6H, C*H*_3_) ppm; protons of the ring C*H* were not observed. ^13^C{^1^H} NMR (C_6_D_6_): δ 273.9 (U*C*H_3_), 246.3 (ring *C*), 193.0 (ring *C*), 3.1 (Si(*C*H_3_)_3_), −1.9 (Si(*C*H_3_)_3_) ppm. ^29^Si{^1^H} NMR (C_6_D_6_): δ −39.7, −52.8 ppm. IR (KBr, cm^–1^): ν 2958 (s), 1253 (s), 1095 (s), 835 (s). Anal. calcd for C_30_H_64_Si_6_U: C, 43.34; H, 7.76. Found: C, 43.36; H, 7.74.

### Preparation of [η^5^-1,2,4-(Me_3_Si)_3_C_5_H_2_]_2_U(NH-*p*-tolyl)_2_ (**3**)

A toluene (10 mL) solution of *p*-toluidine (0.26 g, 2.4 mmol) was added to a toluene (10 mL) solution of [η^5^-1,2,4-(Me_3_Si)_3_C_5_H_2_]_2_UMe_2_ (**2**; 1.00 g, 1.2 mmol) with stirring at room temperature. After the solution was stirred at 60 °C overnight, the solvent was removed. The residue was extracted with *n*-hexane (10 mL × 3) and filtered. The volume of the filtrate was reduced to 10 mL, and brown microcrystals of **3** formed when this solution was kept at −20 °C for 1 day. Microcrystals of **3** were isolated by filtration, quickly washed with cooled *n*-hexane (5 mL), and dried at 50 °C under vacuum overnight. Yield: 1.13 g (93%). M.p.: 170–172 °C (dec.). ^1^H NMR (C_6_D_6_): δ 5.58 (d, *J* = 5.6 Hz, 4H, phenyl), 5.20 (s, 6H, C*H*_3_), 3.30 (s, 18H, Si(C*H*_3_)_3_), −1.12 (s, 36H, Si(C*H*_3_)_3_), −12.48 (s, 4H, phenyl) ppm; protons of Cp-ring C*H* and N*H* were not observed. ^13^C{^1^H} NMR (C_6_D_6_): δ 269.8 (phenyl *C*), 226.3 (phenyl *C*), 185.8 (ring *C*), 184.4 (ring *C*), 148.0 (phenyl *C*), 105.3 (phenyl *C*), 9.2 (*C*H_3_), 5.3 (Si(*C*H_3_)_3_), −0.0 (Si(*C*H_3_)_3_) ppm. ^29^Si{^1^H} NMR (C_6_D_6_): δ −62.4, −64.0 ppm. IR (KBr, cm^–1^): ν 2960 (s), 1514 (s), 1383 (s), 1248 (s), 1095 (m), 831 (s). Anal. calcd for C_42_H_74_N_2_Si_6_U: C, 49.77; H, 7.36; N, 2.76. Found: C, 49.75; H, 7.38; N, 2.78.

### Preparation of [η^5^-1,2,4-(Me_3_Si)_3_C_5_H_2_]_2_U=N(*p*-tolyl)(dmap) (**4**)

A toluene (10 mL) solution of 4-dimethylaminopyridine (dmap; 0.25 g, 2.05 mmol) was added to a toluene (10 mL) solution of [η^5^-1,2,4-(Me_3_Si)_3_C_5_H_2_]_2_UMe_2_ (**2**; 0.82 g, 0.99 mmol) and [η^5^-1,2,4-(Me_3_Si)_3_C_5_H_2_]_2_U(NH-*p*-tolyl)_2_ (**3**; 1.0 g, 0.99 mmol) with stirring at room temperature. After the solution was stirred at 60 °C overnight, the solution was filtered. The volume of the filtrate was reduced to 5 mL, and brown crystals of **4** formed when this solution was kept at −20 °C for 2 days. Crystals of **4** were isolated by filtration, quickly washed with cooled *n*-hexane (5 mL), and dried at 50 °C under vacuum overnight. Yield: 1.77 g (87%). M.p.: 202–204 °C (dec.). ^1^H NMR (C_6_D_6_): δ 53.98 (s, 2H, phenyl), 41.91 (s, 2H, phenyl), 30.98 (t, *J* = 12 Hz, 3H, C*H*_3_), 25.68 (s, 2H, ring C*H*), 5.61 (s, 18H, Si(C*H*_3_)_3_), −3.26 (s, 18H, Si(C*H*_3_)_3_), −8.45 (s, 6H, N(C*H*_3_)_2_), −9.18 (s, 18H, Si(C*H*_3_)_3_), −9.26 (s, 4H, py), −32.81 (s, 2H, ring C*H*) ppm. ^13^C{^1^H} NMR (C_6_D_6_): δ 196.1 (py *C*), 151.3 (py *C*), 150.6 (py *C*), 149.8 (phenyl *C*), 142.6 (phenyl *C*), 141.2 (phenyl *C*), 117.0 (phenyl *C*), 116.4 (ring *C*), 112.4 (ring *C*), 111.0 (ring *C*), 102.6 (ring *C*), 91.1 (ring *C*), 31.4 (N(*C*H_3_)_2_), 31.2 (N(*C*H_3_)_2_), 5.3 (*C*H_3_), −5.8 (Si(*C*H_3_)_3_), −17.2 (Si(*C*H_3_)_3_), −60.4 (Si(*C*H_3_)_3_) ppm. ^29^Si{^1^H} NMR (C_6_D_6_): δ −93.9, −97.4, −115.4 ppm. IR (KBr, cm^–1^): ν 2958 (s), 1616 (s), 1384 (s), 1249 (s), 1095 (m), 1006 (s), 829 (s). Anal. calcd for C_42_H_75_N_3_Si_6_U: C, 49.04; H, 7.35; N, 4.09. Found: C, 49.03; H, 7.36; N, 4.06.

### Preparation of [η^5^-1,2,4-(Me_3_Si)_3_C_5_H_2_]_2_U=O(dmap)·C_6_H_6_ (**5**·C_6_H_6_)

#### Method A

A toluene (10 mL) solution of Ph_2_CO (91 mg, 0.50 mmol) was added to a toluene (10 mL) solution of [η^5^-1,2,4-(Me_3_Si)_3_C_5_H_2_]_2_U=N(*p*-tolyl)(dmap) (**4**; 514 mg, 0.50 mmol) with stirring at room temperature. After this solution was stirred at room temperature overnight, the solvent was removed. The residue was extracted with benzene (10 mL × 3) and filtered. The volume of the filtrate was reduced to 5 mL, and orange crystals of **5**·C_6_H_6_ formed when this solution was kept at 10 °C for 2 days. Crystals of **5**·C_6_H_6_ were isolated by filtration, quickly washed with cooled *n*-hexane (5 mL), and dried at 50 °C under vacuum overnight. Yield: 432 mg (85%). M.p.: 164–166 °C (dec.). ^1^H NMR (C_6_D_6_): δ 20.19 (s, 18H, Si(C*H*_3_)_3_), 7.15 (s, 6H, C_6_*H*_6_), −2.26 (s, 18H, Si(C*H*_3_)_3_), −7.49 (s, 6H, N(C*H*_3_)_2_), −9.38 (s, 18H, Si(C*H*_3_)_3_) ppm; other protons were not observed. ^13^C{^1^H} NMR (C_6_D_6_): δ 200.5 (py *C*), 130.7 (py *C*), 129.6 (py *C*), 129.3 (ring *C*), 128.5 (*C*_6_H_6_), 121.3 (ring *C*), 119.2 (ring *C*), 96.2 (ring *C*), 55.0 (ring *C*), 32.2 (N(*C*H_3_)_2_), 22.2 (Si(*C*H_3_)_3_), −5.3 (Si(*C*H_3_)_3_), −22. 1 (Si(*C*H_3_)_3_) ppm. ^29^Si{^1^H} NMR (C_6_D_6_): δ −35.8, −122.4, −144.9 ppm. IR (KBr, cm^–1^): ν 2953 (s), 1616 (s), 1533 (s), 1444 (s), 1384 (s), 1244 (s), 1095 (s), 1006 (s), 937 (s), 833 (s). Anal. calcd for C_41_H_74_N_2_OSi_6_U: C, 48.39; H, 7.33; N, 2.75. Found: C, 48.42; H, 7.36; N, 2.76.

#### Method B: NMR Scale

A C_6_D_6_ (0.3 mL) solution of Ph_2_CO (3.6 mg, 0.02 mmol) was slowly added to a J. Young NMR tube charged with [η^5^-1,2,4-(Me_3_Si)_3_C_5_H_2_]_2_U=N(*p*-tolyl)(dmap) (**4**; 20.6 mg, 0.02 mmol) and C_6_D_6_ (0.2 mL). Resonances of **5** and those of Ph_2_C=N(*p*-tolyl) (^1^H NMR (C_6_D_6_): δ 7.97 (m, 2H, aryl), 7.12 (m, 3H, aryl), 6.98 (m, 2H, aryl), 6.89 (m, 3H, aryl), 6.77 (m, 4H, aryl), 1.97 (s, 3H, C*H*_3_) ppm)^10b^ were observed by ^1^H NMR spectroscopy (100% conversion) when this solution was kept at room temperature overnight.

### Preparation of [η^5^-1,2,4-(Me_3_Si)_3_C_5_H_2_]_2_U=S(dmap)·0.5C_6_H_6_ (**6**·0.5C_6_H_6_)

#### Method A

This compound was prepared as orange crystals from the reaction of [η^5^-1,2,4-(Me_3_Si)_3_C_5_H_2_]_2_U=N(*p*-tolyl)(dmap) (**4**; 514 mg, 0.50 mmol) and Ph_2_CS (99 mg, 0.50 mmol) in toluene (15 mL) at room temperature and recrystallization from a benzene solution by a similar procedure to that in the synthesis of **5**. The product was isolated by filtration, quickly washed with cooled *n*-hexane (5 mL), and dried at 50 °C under vacuum overnight. Yield: 417 mg (84%). M.p.: 175–177 °C (dec.). ^1^H NMR (C_6_D_6_): δ 9.30 (s, 18H, Si(C*H*_3_)_3_), 7.15 (s, 3H, C_6_*H*_6_), −0.83 (s, 18H, Si(C*H*_3_)_3_), −8.45 (s, 6H, N(C*H*_3_)_2_), −9.13 (s, 18H, Si(C*H*_3_)_3_) ppm; other protons were not observed. ^13^C{^1^H} NMR (C_6_D_6_): δ 196.9 (py *C*), 185.2 (py *C*), 159.8 (py *C*), 130.7 (ring *C*), 129.7 (ring *C*), 129.6 (ring *C*), 129.4 (ring *C*), 128.5 (*C*_6_H_6_), 121.4 (ring *C*), 31.9 (N(*C*H_3_)_2_), 5.9 (Si(*C*H_3_)_3_), −0.7 (Si(*C*H_3_)_3_), −8.8 (Si(*C*H_3_)_3_) ppm. ^29^Si{^1^H} NMR (C_6_D_6_): δ −61.6, −81.6, −111.5 ppm. IR (KBr, cm^–1^): ν 2957 (s), 1614 (s), 1535 (s), 1444 (s), 1384 (s), 1244 (s), 1095 (s), 1006 (s), 833 (s). Anal. calcd for C_38_H_71_N_2_SSi_6_U: C, 45.89; H, 7.20; N, 2.82. Found: C, 45.92; H, 7.18; N, 2.84.

#### Method B: NMR Scale

A C_6_D_6_ (0.3 mL) solution of Ph_2_CS (4.0 mg, 0.02 mmol) was slowly added to a J. Young NMR tube charged with [η^5^-1,2,4-(Me_3_Si)_3_C_5_H_2_]_2_U=N(*p*-tolyl)(dmap) (**4**; 20.6 mg, 0.02 mmol) and C_6_D_6_ (0.2 mL). Resonances of **6** and those of Ph_2_C=N(*p*-tolyl) were observed by ^1^H NMR spectroscopy (100% conversion) when this solution was kept at room temperature overnight.

### Preparation of [η^5^-1,2,4-(Me_3_Si)_3_C_5_H_2_]_2_U=Se(dmap)·0.5C_6_H_6_ (**7**·0.5C_6_H_6_)

#### Method A

This compound was prepared as brown crystals from the reaction of [η^5^-1,2,4-(Me_3_Si)_3_C_5_H_2_]_2_U=N(*p*-tolyl)(dmap) (**4**; 514 mg, 0.50 mmol) and (*p*-MeOPh)_2_CSe (153 mg, 0.50 mmol) in toluene (15 mL) at room temperature and recrystallization from a benzene solution by a similar procedure to that in the synthesis of **5**. The product was isolated by filtration, quickly washed with cooled *n*-hexane (5 mL), and dried at room temperature under vacuum overnight. Yield: 453 mg (87%). M.p.: 153–155 °C (dec.). ^1^H NMR (C_6_D_6_): δ 7.15 (s, 3H, C_6_*H*_6_), 5.18 (s, 18H, Si(C*H*_3_)_3_), 0.92 (s, 18H, Si(C*H*_3_)_3_), −7.62 (s, 18H, Si(C*H*_3_)_3_), −8.71 (s, 6H, N(C*H*_3_)_2_) ppm; other protons were not observed. ^13^C{^1^H} NMR (C_6_D_6_): δ 158.6 (py *C*), 137.4 (py *C*), 133.5 (py *C*), 133.0 (ring *C*), 132.9 (ring *C*), 132.0 (ring *C*), 129.3 (ring *C*), 128.5 (*C*_6_H_6_), 121.7 (ring *C*), 31.9 (N(*C*H_3_)_2_), −0.5 (Si(*C*H_3_)_3_), −0.7 (Si(*C*H_3_)_3_), −2.2 (Si(*C*H_3_)_3_) ppm. ^29^Si{^1^H} NMR (C_6_D_6_): δ −74.2, −75.6, −103.9 ppm. IR (KBr, cm^–1^): ν 2955 (m), 2835 (m), 1604 (s), 1510 (s), 1249 (s), 1170 (s), 1031 (s), 837 (s). Anal. calcd for C_38_H_71_N_2_SeSi_6_U: C, 43.82; H, 6.87; N, 2.69. Found: C, 43.79; H, 6.88; N, 2.73.

#### Method B: NMR Scale

A C_6_D_6_ (0.3 mL) solution of (*p*-MeOPh)_2_CSe (6.1 mg, 0.02 mmol) was slowly added to a J. Young NMR tube charged with [η^5^-1,2,4-(Me_3_Si)_3_C_5_H_2_]_2_U=N(*p*-tolyl)(dmap) (**4**; 20.6 mg, 0.02 mmol) and C_6_D_6_ (0.2 mL). Resonances of **7** and those of (*p*-MeOPh)_2_C=N(*p*-tolyl) (^1^H NMR (C_6_D_6_): δ 7.99 (s, 2H, aryl), 7.20 (s, 2H, aryl), 7.02 (s, 2H, aryl), 6.84 (s, 2H, aryl), 6.78 (s, 2H, aryl), 6.63 (s, 2H, aryl), 3.21 (s, 3H, OC*H*_3_), 3.16 (s, 3H, OC*H*_3_), 2.01 (s, 3H, C*H*_3_) ppm)^19^ were observed by ^1^H NMR spectroscopy (100% conversion) when this solution was kept at room temperature overnight.

### Preparation of [η^5^-1,2,4-(Me_3_Si)_3_C_5_H_2_]_2_U(OSiMe_3_)(Cl) (**8**)

#### Method A

A toluene (10 mL) solution of Me_3_SiCl (28 mg, 0.25 mmol) was added to a toluene (10 mL) solution of [η^5^-1,2,4-(Me_3_Si)_3_C_5_H_2_]_2_U=O(dmap) (**5**; 235 mg, 0.25 mmol) with stirring at room temperature. After the solution was stirred at room temperature for 1 h, the solvent was removed. The residue was extracted with *n*-hexane (10 mL × 3) and filtered. The volume of the filtrate was reduced to 3 mL, and orange crystals of **8** formed when this solution was kept at −20 °C for 2 days. Crystals of **8** were isolated by decantation of the supernatant, rapidly washed with cooled *n*-hexane (2 mL), and dried at 50 °C under vacuum overnight. Yield: 199 mg (86%). M.p.: 175–177 °C (dec.). ^1^H NMR (C_6_D_6_): δ 31.85 (s, 9H, Si(C*H*_3_)_3_), 8.03 (s, 18H, Si(C*H*_3_)_3_), −7.63 (s, 18H, Si(C*H*_3_)_3_), −9.78 (s, 18H, Si(C*H*_3_)_3_) ppm; the protons of Cp-ring C*H* were not observed. ^13^C{^1^H} NMR (C_6_D_6_): δ 165.3 (ring *C*), 157.7 (ring *C*), 121.3 (ring *C*), 83.6 (ring *C*), 82.1 (ring *C*), 11.1 (Si(*C*H_3_)_3_), −6.2 (Si(*C*H_3_)_3_), −17.7 (Si(*C*H_3_)_3_) ppm. ^29^Si{^1^H} NMR (C_6_D_6_): δ −31.5, −86.4, −118.4 ppm. IR (KBr, cm^–1^): ν 2957 (s), 1248 (s), 1089 (s), 991 (s), 839 (s). Anal. calcd for C_31_H_67_ClOSi_7_U: C, 40.21; H, 7.29. Found: C, 40.18; H, 7.31.

#### Method B: NMR Scale

A C_6_D_6_ (0.3 mL) solution of Me_3_SiCl (2.2 mg, 0.02 mmol) was slowly added to a J. Young NMR tube charged with [η^5^-1,2,4-(Me_3_Si)_3_C_5_H_2_]_2_U=O(dmap) (**5**; 18.8 mg, 0.02 mmol) and C_6_D_6_ (0.2 mL). Resonances of **8** and those of dmap were observed by ^1^H NMR spectroscopy (100% conversion) when this solution was kept at room temperature overnight.

### Preparation of [η^5^-1,2,4-(Me_3_Si)_3_C_5_H_2_]_2_U(OSiMe_3_)(I) (**9**)

#### Method A

This compound was prepared as orange crystals from the reaction of [η^5^-1,2,4-(Me_3_Si)_3_C_5_H_2_]_2_U=O(dmap) (**5**; 235 mg, 0.25 mmol) and Me_3_SiI (50 mg, 0.25 mmol) in toluene (15 mL) at room temperature and recrystallization from an *n*-hexane solution by a similar procedure to that in the synthesis of **8**. The product was isolated by decantation of the supernatant, rapidly washed with cooled *n*-hexane (2 mL), and dried at 50 °C under vacuum overnight. Yield: 214 mg (84%). M.p.: 152–156 °C (dec.). ^1^H NMR (C_6_D_6_): δ 36.48 (s, 9H, Si(C*H*_3_)_3_), 10.00 (s, 18H, Si(C*H*_3_)_3_), −8.13 (s, 18H, Si(C*H*_3_)_3_), −10.42 (s, 18H, Si(C*H*_3_)_3_) ppm; the protons of Cp-ring C*H* were not observed. ^13^C{^1^H} NMR (C_6_D_6_): δ 157.9 (ring *C*), 155.6 (ring *C*), 112.6 (ring *C*), 82.4 (ring *C*), 81.4 (ring *C*), 13.8 (Si(*C*H_3_)_3_), −6.6 (Si(*C*H_3_)_3_), −18.1 (Si(*C*H_3_)_3_) ppm. ^29^Si{^1^H} NMR (C_6_D_6_): δ −35.3, −97.5, −132.9 ppm. IR (KBr, cm^–1^): ν 2958 (s), 1248 (s), 1087 (s), 989 (s), 837 (s). Anal. calcd for C_31_H_67_IOSi_7_U: C, 36.60; H, 6.64. Found: C, 36.58; H, 6.66.

#### Method B: NMR Scale

A C_6_D_6_ (0.3 mL) solution of Me_3_SiI (4.0 mg, 0.02 mmol) was slowly added to a J. Young NMR tube charged with [η^5^-1,2,4-(Me_3_Si)_3_C_5_H_2_]_2_U=O(dmap) (**5**; 18.8 mg, 0.02 mmol) and C_6_D_6_ (0.2 mL). Resonances of **9** and those of dmap were observed by ^1^H NMR spectroscopy (100% conversion) when this solution was kept at room temperature overnight.

### Preparation of [η^5^-1,2,4-(Me_3_Si)_3_C_5_H_2_]_2_U(OSiMe_3_)(NC) (**10**)

#### Method A

This compound was prepared as yellow crystals from the reaction of [η^5^-1,2,4-(Me_3_Si)_3_C_5_H_2_]_2_U=O(dmap) (**5**; 235 mg, 0.25 mmol) and Me_3_SiCN (25 mg, 0.25 mmol) in toluene (15 mL) at room temperature and recrystallization from an *n*-hexane solution by a similar procedure to that in the synthesis of **8**. The product was isolated by decantation of the supernatant, rapidly washed with cooled *n*-hexane (2 mL), and dried at 50 °C under vacuum overnight. Yield: 177 mg (78%). M.p.: 163–165 °C (dec.). ^1^H NMR (C_6_D_6_): δ 36.11 (s, 9H, Si(C*H*_3_)_3_), 6.76 (s, 18H, Si(C*H*_3_)_3_), −8.16 (s, 18H, Si(C*H*_3_)_3_), −10.77 (s, 18H, Si(C*H*_3_)_3_) ppm; the protons of Cp-ring C*H* were not observed. ^13^C{^1^H} NMR (C_6_D_6_): δ 180.7 (*C*N), 175.8 (ring *C*), 136.6 (ring *C*), 121.4 (ring *C*), 80.5 (ring *C*), 79.3 (ring *C*), 9.5 (Si(*C*H_3_)_3_), −7.4 (Si(*C*H_3_)_3_), −20.1 (Si(*C*H_3_)_3_) ppm. ^29^Si{^1^H} NMR (C_6_D_6_): δ −48.5, −100.1, −125.1 ppm. IR (KBr, cm^–1^): ν 2954 (s), 2036 (w), 1612 (s), 1249 (s), 1087 (s), 840 (s). Anal. calcd for C_32_H_67_NOSi_7_U: C, 41.94; H, 7.37; N, 1.53. Found: C, 41.92; H, 7.38; N, 1.56.

#### Method B: NMR Scale

A C_6_D_6_ (0.3 mL) solution of Me_3_SiCN (2.0 mg, 0.02 mmol) was slowly added to a J. Young NMR tube charged with [η^5^-1,2,4-(Me_3_Si)_3_C_5_H_2_]_2_U=O(dmap) (**5**; 18.8 mg, 0.02 mmol) and C_6_D_6_ (0.2 mL). Resonances of **10** and those of dmap were observed by ^1^H NMR spectroscopy (100% conversion) when this solution was kept at room temperature overnight.

### Preparation of [η^5^-1,2,4-(Me_3_Si)_3_C_5_H_2_]_2_U(OSiMe_3_)(N_3_) (**11**)

#### Method A

This compound was prepared as brown microcrystals from the reaction of [η^5^-1,2,4-(Me_3_Si)_3_C_5_H_2_]_2_U=O(dmap) (**5**; 235 mg, 0.25 mmol) and Me_3_SiN_3_ (29 mg, 0.25 mmol) in toluene (15 mL) at room temperature and recrystallization from an *n*-hexane solution by a similar procedure to that in the synthesis of **8**. The product was isolated by decantation of the supernatant, rapidly washed with cooled *n*-hexane (2 mL) and, dried at 50 °C under vacuum overnight. Yield: 172 mg (74%). M.p.: 168–170 °C (dec.). ^1^H NMR (C_6_D_6_): δ 28.67 (s, 9H, Si(C*H*_3_)_3_), 6.97 (s, 18H, Si(C*H*_3_)_3_), −7.51 (s, 18H, Si(C*H*_3_)_3_), −9.25 (s, 18H, Si(C*H*_3_)_3_) ppm; the protons of Cp-ring C*H* were not observed. ^13^C{^1^H} NMR (C_6_D_6_): δ 178.1 (ring *C*), 163.6 (ring *C*), 137.0 (ring *C*), 132.5 (ring *C*), 130.6 (ring *C*), 20.8 (Si(*C*H_3_)_3_), −0.7 (Si(*C*H_3_)_3_), −1.3 (Si(*C*H_3_)_3_), −17.0 (Si(*C*H_3_)_3_) ppm. ^29^Si{^1^H} NMR (C_6_D_6_): δ 15.8, −33.7, −83.7, −109.1 ppm. IR (KBr, cm^–1^): ν 2955 (s), 2083 (s), 1612 (s), 1249 (s), 1003 (s), 833 (s). Anal. calcd for C_31_H_67_N_3_OSi_7_U: C, 39.93; H, 7.24; N, 4.51. Found: C, 39.92; H, 7.26; N, 4.49.

#### Method B: NMR Scale

A C_6_D_6_ (0.3 mL) solution of Me_3_SiN_3_ (2.3 mg, 0.02 mmol) was slowly added to a J. Young NMR tube charged with [η^5^-1,2,4-(Me_3_Si)_3_C_5_H_2_]_2_U=O(dmap) (**5**; 18.8 mg, 0.02 mmol) and C_6_D_6_ (0.2 mL). Resonances of **11** and those of dmap were observed by ^1^H NMR spectroscopy (100% conversion) when this solution was kept at room temperature overnight.

### Preparation of [η^5^-1,2,4-(Me_3_Si)_3_C_5_H_2_]_2_U(OSiMe_3_)_2_ (**12**)

#### Method A

After a toluene (10 mL) solution of [η^5^-1,2,4-(Me_3_Si)_3_C_5_H_2_]_2_U(OSiMe_3_)(Cl) (**8**; 185 mg, 0.2 mmol) was stirred at 70 °C overnight, the solution was filtered. The volume of the filtrate was reduced to 5 mL and cooled to −20 °C, yielding red crystals [η^5^-1,2,4-(Me_3_Si)_3_C_5_H_2_]_2_UCl_2_ (**1**), which were isolated by decantation of the supernatant. Yield: 75 mg (43% based on **8**). After the red crystals of **1** were isolated, the solvent of the mother liquid was removed. The residue was extracted with *n*-hexane (5 mL × 3) and filtered. The volume of the filtrate was reduced to 4 mL, yellow crystals of **12** formed when this solution was kept at −40 °C for 2 days. Crystals of **12** were isolated by decantation of the supernatant, quickly washed with *n*-hexane (2 mL), and dried at 50 °C under vacuum overnight. Yield: 74 mg (38% based on **8**). M.p.: 135–137 °C (dec.). ^1^H NMR (C_6_D_6_): δ 36.12 (s, 18H, Si(C*H*_3_)_3_), 8.91 (s, 18H, Si(C*H*_3_)_3_), −7.81 (s, 18H, Si(C*H*_3_)_3_), −10.15 (s, 18H, Si(C*H*_3_)_3_) ppm; the protons of Cp-ring C*H* were not observed. ^13^C{^1^H} NMR (C_6_D_6_): δ 121.4 (ring *C*), 120.3 (ring *C*), 120.2 (ring *C*), 11.1 (Si(*C*H_3_)_3_), −6.5 (Si(*C*H_3_)_3_), −7.4 (Si(*C*H_3_)_3_) ppm. ^29^Si{^1^H} NMR (C_6_D_6_): δ −31.5, −32.2, −91.4 ppm. IR (KBr, cm^–1^): ν 2957 (s), 1614 (m), 1248 (s), 1093 (m), 831 (s). Anal. calcd for C_34_H_76_O_2_Si_8_U: C, 41.68; H, 7.82. Found: C, 41.71; H, 7.79.

#### Method B: NMR Scale

After a C_6_D_6_ (0.5 mL) solution of [η^5^-1,2,4-(Me_3_Si)_3_C_5_H_2_]_2_U(OSiMe_3_)(Cl) (**8**; 185 mg, 0.2 mmol) was kept at 70 °C overnight, resonances of **12** and those of [η^5^-1,2,4-(Me_3_Si)_3_C_5_H_2_]_2_UCl_2_ (**1**) (^1^ H NMR (C_6_D_6_): δ 13.70 (br s, 4H, ring C*H*), 2.79 (s, 36H, Si(C*H*_3_)_3_), −13.39 (s, 18H, Si(C*H*_3_)_3_) ppm)^17^ were observed by ^1^H NMR spectroscopy (100% conversion).

### Preparation of [η^5^-1,2,4-(Me_3_Si)_3_C_5_H_2_]_2_U[OC(=NPh)S)(dmap)·0.5C_6_H_6_ (**13**·0.5C_6_H_6_)

#### Method A

This compound was prepared as orange crystals from the reaction of [η^5^-1,2,4-(Me_3_Si)_3_C_5_H_2_]_2_U=O(dmap) (**5**; 235 mg, 0.25 mmol) and PhNCS (34 mg, 0.25 mmol) in toluene (15 mL) at room temperature and recrystallization from a benzene solution by a similar procedure as that in the synthesis of **8**. The product was isolated by decantation of the supernatant, rapidly washed with cooled *n*-hexane (2 mL), and dried at 50 °C under vacuum overnight. Yield: 239 mg (86%). M.p.: 159–161 °C (dec.). ^1^H NMR (C_6_D_6_): δ 16.09 (s, 2H, phenyl), 8.83 (s, 2H, phenyl), 7.15 (s, 3H, C_6_*H*_6_), 6.39 (br s, 18H, Si(C*H*_3_)_3_), 2.47 (s, 2H, ring C*H*), 1.45 (s, 2H, py *H*), 0.06 (br s, 18H, Si(C*H*_3_)_3_), −2.61 (s, 2H, py *H*), −3.54 (s, 1H, phenyl), −4.48 (s, 6H, N(C*H*_3_)_2_), −7.55 (s, 18H, Si(C*H*_3_)_3_) ppm; two Cp-ring C*H* were not observed. ^13^C{^1^H} NMR (C_6_D_6_): δ 177.2 (*C*N), 167.3 (py *C*), 149.5 (py *C*), 145.3 (py *C*), 140.3 (phenyl *C*), 137.0 (phenyl *C*), 132.5 (phenyl *C*), 130.6 (phenyl *C*), 129.6 (ring *C*), 129.5 (ring *C*), 129.4 (ring *C*), 128.5 (*C*_6_H_6_), 128.4 (ring *C*), 127.1 (ring *C*), 34.7 (N(*C*H_3_)_2_), 10.7 (Si(*C*H_3_)_3_), 6.7 (Si(*C*H_3_)_3_), −2.2 (Si(*C*H_3_)_3_) ppm. ^29^Si{^1^H} NMR (C_6_D_6_): δ −67.9, −97.1, −99.7 ppm. IR (KBr, cm^–1^): ν 2954 (s), 2900 (s), 1612 (s), 1573 (s), 1249 (s), 1095 (s), 995 (s), 840 (s). Anal. calcd for C_45_H_76_N_3_OSSi_6_U: C, 48.53; H, 6.88; N, 3.77. Found: C, 48.52; H, 6.86; N, 3.79.

#### Method B: NMR Scale

A C_6_D_6_ (0.3 mL) solution of PhNCS (2.7 mg, 0.02 mmol) was slowly added to a J. Young NMR tube charged with [η^5^-1,2,4-(Me_3_Si)_3_C_5_H_2_]_2_U=O(dmap) (**5**; 18.8 mg, 0.02 mmol) and C_6_D_6_ (0.2 mL). Resonances of **13** were observed by ^1^H NMR spectroscopy (100% conversion) when this solution was kept at room temperature overnight.

### Preparation of [η^5^-1,2,4-(Me_3_Si)_3_C_5_H_2_]_2_U(SSiMe_3_)(I) (**14**)

#### Method A

This compound was prepared as orange crystals from the reaction of [η^5^-1,2,4-(Me_3_Si)_3_C_5_H_2_]_2_U=S(dmap) (**6**; 239 mg, 0.25 mmol) and Me_3_SiI (50 mg, 0.25 mmol) in toluene (15 mL) at room temperature and recrystallization from an *n*-hexane solution by a similar procedure to that in the synthesis of **8**. The product was isolated by decantation of the supernatant, rapidly washed with cooled *n*-hexane (2 mL), and dried at 50 °C under vacuum overnight. Yield: 222 mg (86%). M.p.: 156–158 °C (dec.). ^1^H NMR (C_6_D_6_): δ 13.24 (s, 18H, Si(C*H*_3_)_3_), 12.98 (s, 2H, ring C*H*), −2.00 (s, 18H, Si(C*H*_3_)_3_), −3.48 (s, 2H, ring C*H*), −7.66 (s, 9H, Si(C*H*_3_)_3_), −13.46 (s, 18H, Si(C*H*_3_)_3_) ppm. ^13^C{^1^H} NMR (C_6_D_6_): δ 130.6 (ring *C*), 129.7 (ring *C*), 129.6 (ring *C*), 129.4 (ring *C*), 121.4 (ring *C*), 28.6 (Si(*C*H_3_)_3_), 16.8 (Si(*C*H_3_)_3_), 13.8 (Si(*C*H_3_)_3_), 5.5 (Si(*C*H_3_)_3_) ppm. ^29^Si{^1^H} NMR (C_6_D_6_): δ −11.4, −52.4, −86.6, −114.4 ppm. IR (KBr, cm^–1^): ν 2955 (s), 1400 (s), 1244 (s), 1087 (s), 989 (s), 929 (s), 823 (s). Anal. calcd for C_31_H_67_ISSi_7_U: C, 36.03; H, 6.53. Found: C, 36.05; H, 6.56.

#### Method B: NMR Scale

A C_6_D_6_ (0.3 mL) solution of Me_3_SiI (4.0 mg, 0.02 mmol) was slowly added to a J. Young NMR tube charged with [η^5^-1,2,4-(Me_3_Si)_3_C_5_H_2_]_2_U=S(dmap) (**6**; 19.1 mg, 0.02 mmol) and C_6_D_6_ (0.2 mL). Resonances of **14** and those of dmap were observed by ^1^H NMR spectroscopy (100% conversion) when this solution was kept at room temperature overnight.

### Reaction of [η^5^-1,2,4-(Me_3_Si)_3_C_5_H_2_]_2_U=S(dmap) (**6**) with Me_3_SiCl

#### NMR Scale

A C_6_D_6_ (0.3 mL) solution of Me_3_SiCl (2.2 mg, 0.02 mmol) was slowly added to a J. Young NMR tube charged with [η^5^-1,2,4-(Me_3_Si)_3_C_5_H_2_]_2_U=S(dmap) (**6**; 19.1 mg, 0.02 mmol) and C_6_D_6_ (0.2 mL). Resonances of **1** along with those of unreacted **6**, (Me_3_Si)_2_S (^1^H NMR (C_6_D_6_): δ 0.29 (s, 18H, Si(C*H*_3_)_3_) ppm), and dmap were observed by ^1^H NMR spectroscopy (50% conversion based on **6**) when this solution was kept at room temperature overnight. Nevertheless, when 2 equiv of Me_3_SiCl (4.4 mg, 0.04 mmol) was added to a C_6_D_6_ (0.5 mL) solution of [η^5^-1,2,4-(Me_3_Si)_3_C_5_H_2_]_2_U=S(dmap) (**6**; 19.1 mg, 0.02 mmol), resonances of **1** along with those of (Me_3_Si)_2_S and dmap were observed by ^1^H NMR spectroscopy (100% conversion) when this solution was kept at room temperature overnight.

### Reaction of [η^5^-1,2,4-(Me_3_Si)_3_C_5_H_2_]_2_U=S(dmap) (**6**) with PhNCO

#### NMR Scale

A C_6_D_6_ (0.3 mL) solution of PhNCO (2.4 mg, 0.02 mmol) was slowly added to a J. Young NMR tube charged with [η^5^-1,2,4-(Me_3_Si)_3_C_5_H_2_]_2_U=S(dmap) (**6**; 19.1 mg, 0.02 mmol) and C_6_D_6_ (0.2 mL). Resonances of **13** were observed by ^1^H NMR spectroscopy (100% conversion) when this solution was kept at room temperature overnight.

### Preparation of [η^5^-1,2,4-(Me_3_Si)_3_C_5_H_2_]_2_U[SC(=NPh)S)(dmap) (**15**)

#### Method A

This compound was prepared as brown crystals from the reaction of [η^5^-1,2,4-(Me_3_Si)_3_C_5_H_2_]_2_U=S(dmap) (**6**; 239 mg, 0.25 mmol) and PhNCS (34 mg, 0.25 mmol) in toluene (15 mL) at room temperature and recrystallization from a benzene solution by a similar procedure to that in the synthesis of **8**. The product was isolated by decantation of the supernatant, rapidly washed with cooled *n*-hexane (2 mL), and dried at 50 °C under vacuum overnight. Yield: 224 mg (82%). M.p.: 165–167 °C (dec.). ^1^H NMR (C_6_D_6_): δ 9.13 (s, 2H, phenyl), 6.59 (s, 2H, phenyl), 2.41 (s, 18H, Si(C*H*_3_)_3_), 1.38 (s, 18H, Si(C*H*_3_)_3_), −0.84 (s, 2H, py *H*), −2.59 (s, 18H, Si(C*H*_3_)_3_), −3.75 (s, 6H, N(C*H*_3_)_2_), −4.94 (s, 2H, ring C*H*), −8.72 (s, 1H, phenyl), −9.02 (s, 2H, py *H*) ppm; two Cp-ring C*H* protons were not observed. ^13^C{^1^H} NMR (C_6_D_6_): δ 144.7 (*C*N), 136.6 (py *C*), 134.8 (py *C*), 133.4 (py *C*), 129.5 (phenyl *C*), 127.2 (phenyl *C*), 127.1 (phenyl *C*), 125.7 (phenyl *C*), 122.5 (ring *C*), 95.5 (ring *C*), 35.8 (N(*C*H_3_)_2_), 14.0 (Si(*C*H_3_)_3_), 12.3 (Si(*C*H_3_)_3_), 3.8 (Si(*C*H_3_)_3_) ppm. ^29^Si{^1^H} NMR (C_6_D_6_): δ −46.9, −56.4, −110.6 ppm. IR (KBr, cm^–1^): ν 2954 (m), 1612 (s), 1535 (s), 1249 (s), 1087 (s), 995 (s), 833 (s). Anal. calcd for C_42_H_73_N_3_S_2_Si_6_U: C, 46.25; H, 6.75; N, 3.85. Found: C, 46.22; H, 6.76; N, 3.87.

#### Method B: NMR Scale

A C_6_D_6_ (0.3 mL) solution of PhNCS (2.7 mg, 0.02 mmol) was slowly added to a J. Young NMR tube charged with [η^5^-1,2,4-(Me_3_Si)_3_C_5_H_2_]_2_U=S(dmap) (**6**; 19.1 mg, 0.02 mmol) and C_6_D_6_ (0.2 mL). Resonances of **15** were observed by ^1^H NMR spectroscopy (100% conversion) when this solution was kept at room temperature overnight.

### Preparation of {[η^5^-1,2,4-(Me_3_Si)_3_C_5_H_2_]_2_U}_2_(μ-CS_3_)_2_ (**16**)

#### Method A

This compound was prepared as brown crystals from the reaction of [η^5^-1,2,4-(Me_3_Si)_3_C_5_H_2_]_2_U=S(dmap) (**6**; 239 mg, 0.25 mmol) and CS_2_ (19 mg, 0.25 mmol) in toluene (15 mL) at room temperature and recrystallization from a benzene solution by a similar procedure to that in the synthesis of **8**. The product was isolated by decantation of the supernatant, rapidly washed with cooled *n*-hexane (2 mL), and dried at 50 °C under vacuum overnight. Yield: 205 mg (90%). ^1^H NMR (C_6_D_6_): δ 20.21 (s, 9H, Si(C*H*_3_)_3_), 14.23 (br s, 9H, Si(C*H*_3_)_3_), −7.42 (s, 9H, Si(C*H*_3_)_3_), −7.68 (s, 9H, Si(C*H*_3_)_3_), −10.16 (s, 9H, Si(C*H*_3_)_3_), −12.98 (s, 9H, Si(C*H*_3_)_3_) ppm; the protons of the Cp-ring C*H* were not observed. These spectroscopic data agreed with those reported in the literature.^17^ Furthermore, this complex was also characterized by X-ray diffraction analysis and its molecular structure is shown in the Supporting Information.

#### Method B: NMR Scale

A C_6_D_6_ (0.3 mL) solution of CS_2_ (1.5 mg, 0.02 mmol) was slowly added to a J. Young NMR tube charged with [η^5^-1,2,4-(Me_3_Si)_3_C_5_H_2_]_2_U=S(dmap) (**6**; 19.1 mg, 0.02 mmol) and C_6_D_6_ (0.2 mL). Resonances of **16** and those of dmap were observed by ^1^H NMR spectroscopy (100% conversion) when this solution was kept at room temperature overnight.

### Preparation of {[η^5^-1,2,4-(Me_3_Si)_3_C_5_H_2_]_2_U}_2_(μ-Se)_2_ (**17**)

After a toluene (10 mL) solution of [η^5^-1,2,4-(Me_3_Si)_3_C_5_H_2_]_2_U=Se(dmap) (**7**; 193 mg, 0.2 mmol) was stirred at 60 °C overnight, the solvent was removed. The residue was extracted with benzene (5 mL × 3) and filtered. The volume of the filtrate was reduced to 4 mL, and brown microcrystals of **17** formed when this solution was kept at 10 °C for 2 days. The product was isolated by decantation of the supernatant, rapidly washed with cooled *n*-hexane (2 mL), and dried at 50 °C under vacuum overnight. Yield: 144 mg (82%). M.p.: 183–185 °C (dec.). ^1^H NMR (C_6_D_6_): δ 36.44 (s, 18H, Si(C*H*_3_)_3_), 13.92 (s, 36H, Si(C*H*_3_)_3_) ppm; the protons of Cp-ring C*H* were not observed. ^13^C{^1^H} NMR (C_6_D_6_): δ 150.1 (ring *C*), 139.0 (ring *C*), 137.4 (ring *C*), 4.6 (Si(*C*H_3_)_3_), 2.7 (Si(*C*H_3_)_3_) ppm. ^29^Si{^1^H} NMR (C_6_D_6_): δ −74.4, −75.8, −104.0 ppm. IR (KBr, cm^–1^): ν 2957 (s), 1595 (s), 1510 (s), 1249 (s), 1172 (s), 1030 (s), 831 (s). Anal. calcd for C_56_H_116_Se_2_Si_12_U_2_: C, 38.20; H, 6.64. Found: C, 38.23; H, 6.61. Brown crystals of **17**·0.5C_6_H_14_ suitable for X-ray structural analysis were isolated from a mixture of benzene and *n*-hexane (4:1) solution.

### Preparation of [η^5^-1,2,4-(Me_3_Si)_3_C_5_H_2_]_2_U(SeSiMe_3_)(I) (**18**)

#### Method A

This compound was prepared as orange crystals from the reaction of [η^5^-1,2,4-(Me_3_Si)_3_C_5_H_2_]_2_U=Se(dmap) (**7**; 241 mg, 0.25 mmol) and Me_3_SiI (50 mg, 0.25 mmol) in toluene (15 mL) at room temperature and recrystallization from an *n*-hexane solution by a similar procedure as that in the synthesis of **8**. The product was isolated by decantation of the supernatant, rapidly washed with cooled *n*-hexane (2 mL), and dried at 50 °C under vacuum overnight. Yield: 221 mg (85%). M.p.: 164–166 °C (dec.). ^1^H NMR (C_6_D_6_): δ 14.03 (s, 18H, (C*H*_3_)_3_Si), −1.25 (s, 18H, (C*H*_3_)_3_Si), −9.50 (s, 9H, (C*H*_3_)_3_Si), −13.57 (s, 18H, (C*H*_3_)_3_Si) ppm; the protons of Cp-ring C*H* were not observed. ^13^C{^1^H} NMR (C_6_D_6_): δ 131.4 (ring *C*), 129.5 (ring *C*), 121.7 (ring *C*), 113.7 (ring *C*), 113.6 (ring *C*) 21.7 (Si(*C*H_3_)_3_), 13.8 (Si(*C*H_3_)_3_), 8.4 (Si(*C*H_3_)_3_), 6.2 (Si(*C*H_3_)_3_) ppm. ^29^Si{^1^H} NMR (C_6_D_6_): δ −11.5, −35.3, −53.5 ppm. IR (KBr, cm^–1^): ν 2954 (s), 1597 (m), 1512 (s), 1381 (s), 1249 (s), 1172 (m), 1087 (m), 1026 (m), 833 (s). Anal. calcd for C_31_H_67_ISeSi_7_U: C, 34.46; H, 6.25. Found: C, 34.45; H, 6.26. This complex was also characterized by X-ray diffraction analysis and its molecular structure is shown in the Supporting Information. Nevertheless, the quality of the data was rather poor because of crystal twinning, but sufficient to at least establish the overall connectivity.

#### Method B: NMR Scale

A C_6_D_6_ (0.3 mL) solution of Me_3_SiI (4.0 mg, 0.02 mmol) was slowly added to a J. Young NMR tube charged with [η^5^-1,2,4-(Me_3_Si)_3_C_5_H_2_]_2_U=Se(dmap) (**7**; 19.3 mg, 0.02 mmol) and C_6_D_6_ (0.2 mL). Resonances of **18** and those of dmap were observed by ^1^H NMR spectroscopy (100% conversion) when this solution was kept at room temperature overnight.

### Reaction of [η^5^-1,2,4-(Me_3_Si)_3_C_5_H_2_]_2_U=Se(dmap) (**7**) with Me_3_SiCl

#### NMR Scale

A C_6_D_6_ (0.3 mL) solution of Me_3_SiCl (2.2 mg, 0.02 mmol) was slowly added to a J. Young NMR tube charged with [η^5^-1,2,4-(Me_3_Si)_3_C_5_H_2_]_2_U=Se(dmap) (**7**; 19.3 mg, 0.02 mmol) and C_6_D_6_ (0.2 mL). Resonances of **1** along with those of unreacted **7**, (Me_3_Si)_2_Se (^1^ H NMR (C_6_D_6_): δ 0.36 (s, 18H, Si(C*H*_3_)_3_) ppm), and dmap were observed by ^1^H NMR spectroscopy (50% conversion based on **7**) when this solution was kept at room temperature overnight. Nevertheless, when 2 equiv of Me_3_SiCl (4.4 mg, 0.04 mmol) was added to a C_6_D_6_ (0.5 mL) solution of [η^5^-1,2,4-(Me_3_Si)_3_C_5_H_2_]_2_U=Se(dmap) (**7**; 19.3 mg, 0.02 mmol), resonances of **1** along with those of (Me_3_Si)_2_Se and dmap were observed by ^1^H NMR spectroscopy (100% conversion) when this solution was kept at room temperature overnight.

### X-ray Crystallography

Single-crystal X-ray diffraction measurements were carried out on a Rigaku Saturn CCD diffractometer at 100(2) K using Mο Kα radiation (λ = 0.71073 Å) or Cu Kα radiation (λ = 1.54184 Å). An empirical absorption correction was applied using the SADABS program.^20^ All structures were solved by direct methods and refined by full-matrix least squares on *F*^2^ using the SHELXL program package.^21^ All the hydrogen atoms were geometrically fixed using the riding model. The crystal data and experimental data for **4**–**10** and **12**–**17** are summarized in the Supporting Information. Selected bond lengths and angles are listed in Table 1.

### Computational Methods

All calculations were carried out with the Gaussian 09 program (G09),^22^ employing the B3PW91 functional, plus a polarizable continuum model (PCM) (denoted as B3PW91-PCM), with standard 6-31G(d) basis set for C, H, N, O, S, Se, Si, and Cl elements and a quasi-relativistic 5f-in-valence effective-core potential (ECP60MWB or ECP80MWB) treatment with 60 or 80 electrons in the core region for U and the corresponding optimized segmented ((14s13p10d8f6g)/[10s9p5d4f3g]) basis set for the valence shells of U,^23^ to fully optimize the structures of reactants, complexes, transition state, intermediates, and products and also to mimic the experimental toluene-solvent conditions (dielectric constant ε = 2.379). All stationary points were subsequently characterized by vibrational analyses, from which their respective zero-point (vibrational) energy (ZPE) was extracted and used in the relative energy determinations; in addition, frequency calculations were also performed to ensure that the reactant, complex, intermediate, product, and transition state structures resided at minima and 1st-order saddle points on their potential energy hypersurfaces. Please note that to properly describe the reaction profile for the reaction of **4** + Ph_2_CO, dispersion effects (D3)^24^ had to be considered for the sterically encumbered uranium complexes since dispersion contributes significantly to the overall energy profile (see the Supporting Information for details, Figure S4 and Tables S6 and S7) and therefore single-point B3PW91-PCM-D3^24^ calculations based on B3PW91-PCM geometries were performed for this transformation. Bader’s quantum theory of atoms-in-molecules (QTAIM)^25^ analyses were performed using the Multiwfn program.^26^

## Supporting Information Available

Crystal parameters for compounds **4**–**10** and **12**–**17** (PDF); X-ray crystallographic data, in CIF format
Cartesian coordinates of all stationary points optimized at the B3PW91-PCM level (XYZ)

## Supplementary Material

ja3c03753_si_001.pdf

ja3c03753_si_002.xyz
