# Supplementary material for: A Comprehensive Study Concerning the Synthesis, Structure, and Reactivity of Terminal Uranium Oxido, Sulfido, and Selenido Metallocenes
Source: J Am Chem Soc. 2023 Jun 28;145(27):14839–55. doi: 10.1021/jacs.3c03753 (PMC10347556; doi:10.1021/jacs.3c03753)
Supplement: Supplementary file 1 — ja3c03753_si_001.pdf [file ja3c03753_si_001.pdf]

Supporting Information for

A Comprehensive Study Concerning the Synthesis, Structure and Reactivity of Terminal Uranium Oxido, Sulfido and Selenido Metallocenes

Tongyu Li,[†] Dongwei Wang,[†] Yi Heng,[†] Guohua Hou,[†] Guofu Zi,*[,†] Wanjian Ding,*[,†] and Marc D. Walter*[,‡]

[†]Department of Chemistry, Beijing Normal University, Beijing 100875, China

[‡]Institut für Anorganische und Analytische Chemie, Technische Universität Braunschweig, Hagenring 30, 38106 Braunschweig, Germany

Table of contents

1. **Figures** **S2**
2. **Crystallographic details** **S4**
3. **Coputational details** **S7**

1. Figures

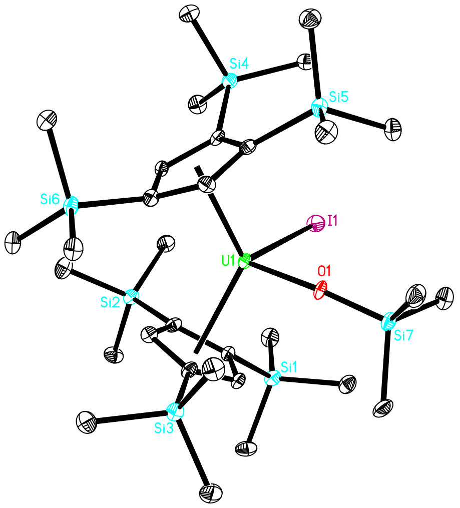

Figure S1. Molecular structure of **9** (thermal ellipsoids drawn at the 35% probability level).

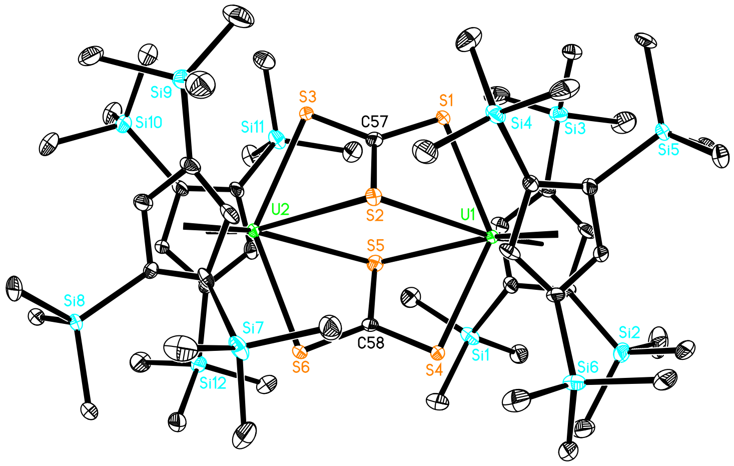

Figure S2. Molecular structure of **16** (thermal ellipsoids drawn at the 35% probability level).

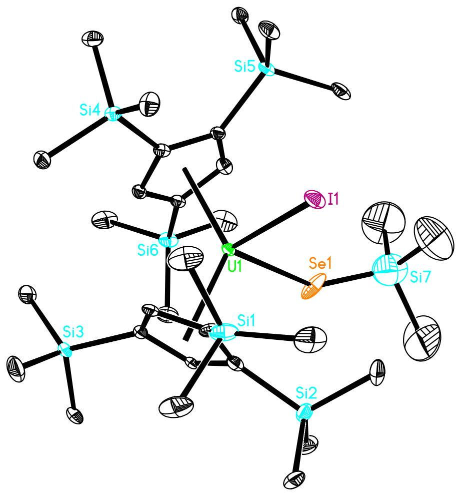

Figure S3. Molecular structure of **18** (thermal ellipsoids drawn at the 35% probability level).

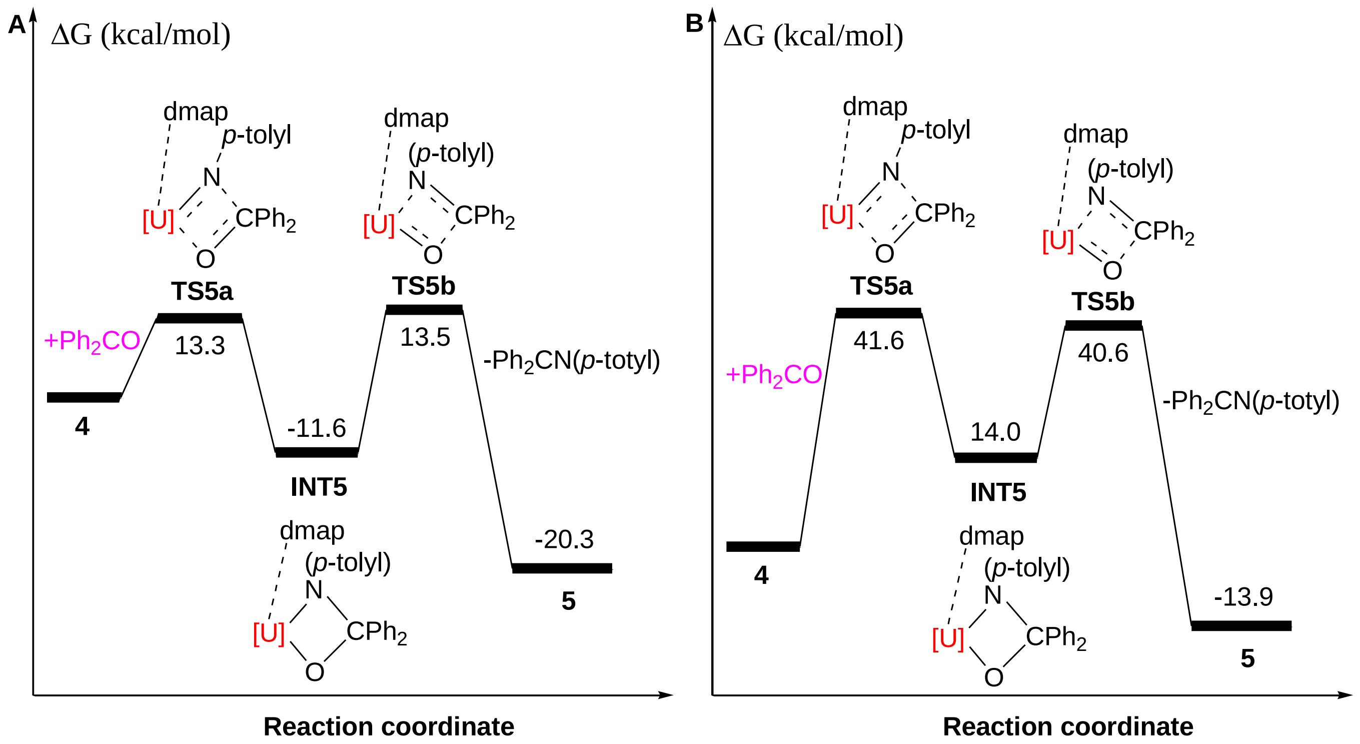

Figure S4. Energy profile (kcal/mol) for the reaction of **4**+Ph_2CO (computed at T = 298 K), obtained with B3PW91-PCM-D3 (**A**, left one) and B3PW91-PCM (**B**, right one) methods. [U] = [η^5-1,2,4-$(Me_3Si)_3C_5H_2]_2U$.

2. Crystallographic details

Table S1. Crystal Data and Experimental Parameters for Compounds 4-8

Compound	**4**	**5**·C_6H_6	**6**·0.5C_6H_6	**7**·0.5C_6H_6	**8**
Formula	$C_{42}H_{75}N_3Si_6U$	$C_{41}H_{74}N_2OSi_6U$	$C_{38}H_{71}N_2SSi_6U$	$C_{38}H_{71}N_2SeSi_6U$	$C_{31}H_{67}ClOSi_7U$
Fw	1028.62	1017.59	994.59	1041.49	925.95
crystal system	triclinic	monoclinic	triclinic	triclinic	triclinic
space group	$P\bar{1}$	$P2_1/n$	$P\bar{1}$	$P\bar{1}$	$P\bar{1}$
a (Å)	11.493(1)	12.639(1)	10.004(1)	10.076(1)	10.985(1)
b (Å)	11.518(1)	27.082(1)	12.878(1)	12.994(1)	12.177(1)
c (Å)	20.169(1)	14.911(1)	19.948(1)	19.915(1)	18.992(1)
α (deg)	85.68(1)	90	75.75(1)	75.47(1)	75.80(1)
β (deg)	82.92(1)	102.60(1)	76.63(1)	76.93(1)	89.53(1)
γ (deg)	77.04(1)	90	78.69(1)	78.31(1)	66.55(1)
V (Å^3)	2578.87(19)	4980.82(12)	2396.75(9)	2429.1(3)	2248.05(10)
Z	2	4	2	2	2
D_{calc} (g/cm^3)	1.325	1.357	1.378	1.424	1.368
μ(Mo/Kα)$_{calc}$ (cm^{-1})	10.405	10.778	11.566	4.264	12.648
size (mm)	0.15 × 0.15 × 0.15	0.15 × 0.15 × 0.15	0.10 × 0.10 × 0.10	0.15 × 0.10 × 0.10	0.15 × 0.15 × 0.15
F(000)	1048	2072	1010	1049	936
2θ range (deg)	7.89 to 153.07	6.53 to 152.53	7.16 to 153.38	6.88 to 59.07	8.21 to 144.40
no. of reflns, collected	33491	36940	31167	25021	15917
no of obsd reflns	10433	10021	9653	11545	8613
no of variables	490	481	453	453	391
abscorr (T_{max}, T_{min})	1.00, 0.60	1.00, 0.53	1.00, 0.65	1.00, 0.67	1.00, 0.77
R	0.049	0.033	0.046	0.050	0.032
R_w	0.121	0.084	0.108	0.080	0.084
R_{all}	0.053	0.035	0.053	0.072	0.033
Gof	1.03	1.02	1.05	0.97	1.05
CCDC	2251181	2251185	2251190	2251188	2251178

Table S2. Crystal Data and Experimental Parameters for Compounds 9, 10, 12 and 13

Compound	**9**	**10**	**12**	2(**13**)·C_6H_6
Formula	$C_{31}H_{67}IOSi_7U$	$C_{32}H_{67}NOSi_7U$	$C_{34}H_{76}O_2Si_8U$	$C_{90}H_{152}N_6O_2S_2Si_{12}U_2$
Fw	1017.40	916.52	979.69	2227.43
crystal system	triclinic	triclinic	monoclinic	monoclinic
space group	$P\bar{1}$	$P\bar{1}$	$P2_1/n$	$P2_1/c$
a (Å)	10.958(1)	11.024(1)	11.771(1)	21.335(1)
b (Å)	12.345(1)	12.150(1)	19.486(1)	11.945(1)
c (Å)	18.951(1)	19.006(1)	21.351(1)	43.300(2)
α (deg)	76.30(1)	75.86(1)	90	90
β (deg)	89.94(1)	89.47(1)	97.70(1)	95.13(1)
γ (deg)	66.52	66.25(1)	90	90
V (Å^3)	2271.85(9)	2247.96(16)	4852.97(15)	10990.6(8)
Z	2	2	4	4
D_{calc} (g/cm^3)	1.487	1.354	1.341	1.346
μ(Mo/Kα)$_{calc}$ (cm^{-1})	17.310	12.117	11.496	10.169
size (mm)	0.15 × 0.05 × 0.05	0.20 × 0.15 × 0.15	0.15 × 0.15 × 0.10	0.10 × 0.10 × 0.10
F(000)	1008	928	2000	4536
2θ range (deg)	8.08 to 153.08	8.24 to 144.26	8.15 to 152.94	7.68 to 154.83
no. of reflns, collected	29192	16306	34241	75121
no of obsd reflns	9170	8611	9651	22010
no of variables	392	400	431	1067
abscorr (T_{max}, T_{min})	1.00, 0.58	1.00, 0.53	1.00, 0.60	1.00, 0.44
R	0.041	0.028	0.044	0.071
R_w	0.108	0.072	0.123	0.162
R_{all}	0.045	0.029	0.048	0.132
Gof	1.09	1.05	1.09	1.03
CCDC	2251179	2251180	2251183	2251186

Table S3. Crystal Data and Experimental Parameters for Compounds 14-17

Compound	**14**	**15**	**16**	**17**·0.5C_6H_{12}
Formula	$C_{31}H_{67}ISSi_7U$	$C_{42}H_{73}N_3S_2Si_6U$	$C_{58}H_{116}S_6Si_{12}U_2$	$C_{59}H_{123}Se_2Si_{12}U$
Fw	1033.46	1090.72	1819.00	1803.63
crystal system	triclinic	monoclinic	monoclinic	triclinic
space group	$P\bar{1}$	$P2_1/c$	$P2_1/n$	$P\bar{1}$
a (Å)	11.702(1)	19.348(1)	16.390(1)	11.646(1)
b (Å)	11.955(1)	13.218(1)	21.905(1)	12.330(1)
c (Å)	19.265(1)	20.955(1)	23.290(1)	29.785(1)
α (deg)	72.55(1)	90	90	83.25(10
β (deg)	79.17(1)	100.89(1)	97.65(1)	86.38(1)
γ (deg)	65.19(1)	90	90	69.88(1)
V (Å^3)	2327.7(2)	5262.6(2)	8287.2(3)	3987.04(16)
Z	2	4	4	2
D_{calc} (g/cm^3)	1.475	1.377	1.458	1.502
μ(Mo/Kα)$_{calc}$ (cm^{-1})	4.393	10.953	14.227	14.348
size (mm)	0.20 × 0.20 × 0.20	0.10 × 0.10 × 0.10	0.10 × 0.10 × 0.10	0.15 × 0.05 × 0.05
F(000)	1024	2216	3648	1794
2θ range (deg)	6.58 to 59.69	7.95 to 153.28	6.77 to 153.61	7.68 to 153.29
no. of reflns, collected	22510	38719	69714	51271
no of obsd reflns	11074	10677	16731	15978
no of variables	391	507	739	713
abscorr (T_{max}, T_{min})	1.00, 0.71	1.00, 0.75	1.00, 0.68	1.00, 0.26
R	0.044	0.047	0.074	0.052
R_w	0.072	0.115	0.169	0.131
R_{all}	0.069	0.060	0.089	0.072
Gof	0.97	1.03	1.04	1.03
CCDC	2251182	2251184	2251187	2251189

3. Coputational details

Table S4. The optimized Cartesian Coordinates (in Å) and structures (the hydrogen atoms omitted for clarity) of stationary points for **4**+Ph_2CO, obtained with B3PW91-PCM/ECP80MWB method.

Species	Cartesian coordinates				
**4**	U	8.960975	3.916231	4.786898	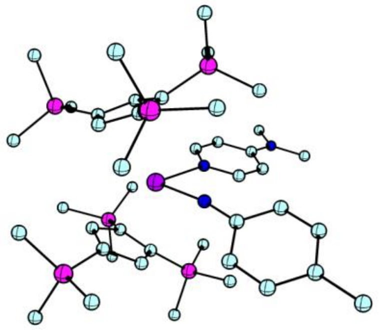 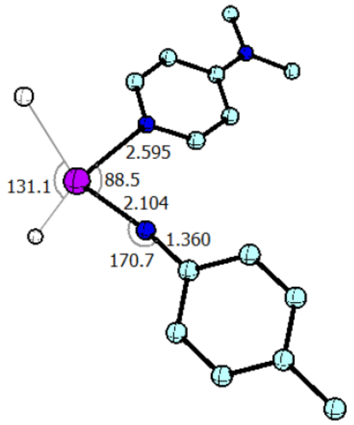
	Si	12.615783	1.980405	2.871158	
	Si	10.489735	-0.037137	5.576265	
	Si	7.114583	1.955841	1.398367	
	Si	5.820552	6.609178	4.758685	
	Si	8.866759	7.525246	7.138029	
	Si	10.745745	6.923677	1.799893	
	N	7.448686	3.332737	6.128645	
	N	10.638044	3.871151	6.766481	
	N	13.175000	3.733297	10.115310	
	C	10.774471	1.993563	3.289599	
	C	10.002561	1.221729	4.256675	
	C	8.647557	1.274949	3.831426	
	H	7.825428	0.775172	4.333200	
	C	8.512247	2.070772	2.653579	
	C	9.843395	2.498483	2.347281	
	H	10.107560	3.095664	1.480871	
	C	12.847854	2.754232	1.157909	
	H	12.313929	2.185427	0.387777	
	H	13.913073	2.748112	0.894306	
	H	12.504988	3.792718	1.106237	
	C	13.760988	2.906359	4.065209	
	H	13.600446	3.989926	4.018823	
	H	14.805234	2.722617	3.780973	
	H	13.638344	2.589110	5.105663	
	C	13.244856	0.199214	2.722685	
	H	13.269965	-0.333805	3.678121	
	H	14.266179	0.202725	2.320309	
	H	12.617250	-0.378627	2.034239	
	C	12.104284	0.359331	6.483248	
	H	12.970664	0.378455	5.814007	
	H	12.292237	-0.416075	7.237229	
	H	12.058998	1.321843	7.003211	
	C	10.661646	-1.743431	4.764240	
	H	9.719338	-2.031486	4.282748	
	H	10.897123	-2.506601	5.517379	
	H	11.444959	-1.772251	4.000041	
	C	9.097409	-0.189063	6.847064	
	H	8.826853	0.770799	7.296848	
	H	9.396444	-0.873374	7.651262	
	H	8.186281	-0.597008	6.394512	
	C	6.944642	3.538697	0.378334	
	H	6.630480	4.390564	0.990403	
	H	6.193608	3.401829	-0.409691	
	H	7.889186	3.805879	-0.109432	
	C	7.557805	0.548587	0.207230	
	H	8.502500	0.749004	-0.312154	
	H	6.777434	0.416193	-0.552991	
	H	7.670004	-0.401252	0.743359	
	C	5.468274	1.531814	2.219529	
	H	5.545715	0.622749	2.827473	
	H	4.703977	1.350020	1.453501	

	H 5.106080 2.339612 2.863442<br>C 7.691514 6.438738 4.546572<br>C 8.803897 6.730176 5.425459<br>C 9.979198 6.707960 4.610713<br>H 10.975651 6.941101 4.975174<br>C 9.662113 6.441911 3.252891<br>C 8.247366 6.240262 3.243540<br>H 7.660057 6.053312 2.348421<br>C 4.935501 5.306171 3.712171<br>H 5.256095 5.311075 2.664247<br>H 3.853252 5.487051 3.724009<br>H 5.109871 4.305985 4.124394<br>C 5.173436 6.433502 6.521183<br>H 5.434499 5.456031 6.940268<br>H 4.078011 6.503365 6.497266<br>H 5.534191 7.211693 7.200852<br>C 5.333127 8.315689 4.083782<br>H 5.819965 9.127879 4.635703<br>H 4.248128 8.467206 4.150419<br>H 5.620627 8.415719 3.030014<br>C 7.922376 9.168721 7.081966<br>H 8.328526 9.817276 6.296436<br>H 8.017583 9.699338 8.038110<br>H 6.854178 9.037185 6.881612<br>C 10.671691 7.961019 7.525928<br>H 11.313580 7.073765 7.570716<br>H 10.723421 8.453045 8.505652<br>H 11.095098 8.652288 6.787797<br>C 8.228465 6.484117 8.579445<br>H 7.231786 6.074359 8.397829<br>H 8.182115 7.100818 9.486708<br>H 8.902286 5.644372 8.781558<br>C 10.446597 8.765282 1.463077<br>H 9.397742 8.954306 1.205042<br>H 11.066844 9.120910 0.630334<br>H 10.684006 9.372787 2.344371<br>C 12.575819 6.713583 2.235748<br>H 12.842996 7.327109 3.104904<br>H 13.208235 7.040160 1.400992<br>H 12.839308 5.675445 2.463535<br>C 10.316360 5.991607 0.211952<br>H 10.548679 4.923432 0.265325<br>H 10.878564 6.410634 -0.632023<br>H 9.250163 6.090862 -0.023909<br>C 6.462332 2.771089 6.877458<br>C 6.369089 2.969780 8.280111<br>H 7.092433 3.625352 8.759130<br>C 5.376551 2.361448 9.044418<br>H 5.352324 2.544767 10.118810<br>C 4.405276 1.532828 8.473371<br>C 4.474860 1.340779 7.084718<br>H 3.733643 0.706572 6.597791<br>C 5.463580 1.932208 6.310996<br>H 5.491320 1.759424 5.238157<br>C 3.329393 0.879928 9.302493<br>H 3.435230 1.135914 10.362610<br>H 3.359994 -0.215424 9.224436<br>H 2.323842 1.191506 8.988730<br>C 10.235813 3.345177 7.944801<br>H 9.215696 2.976940 7.963215<br>C 11.031503 3.275157 9.069616<br>H 10.615491 2.834291 9.967081	

	C 12.355694 3.779452 9.036410<br>C 12.767512 4.338847 7.799837<br>H 13.757961 4.756399 7.667012<br>C 11.894600 4.358697 6.732127<br>H 12.214708 4.792063 5.790285<br>C 14.517740 4.283069 10.034391<br>H 15.121148 3.762282 9.279949<br>H 15.010262 4.166770 11.000034<br>H 14.498704 5.352664 9.790004<br>C 12.710002 3.140353 11.358427<br>H 11.850329 3.685672 11.768270<br>H 13.514694 3.174857 12.093211<br>H 12.423248 2.090882 11.217464	
**Ph_2CO**	O 8.848129 4.136323 6.173403<br>C 7.745315 4.141271 6.709492<br>C 7.054697 2.847191 7.002895<br>C 5.659034 2.714764 7.005136<br>C 7.850172 1.709260 7.201660<br>C 5.072248 1.467982 7.215150<br>C 7.263403 0.470225 7.431607<br>C 5.871805 0.347490 7.438301<br>H 5.031296 3.580494 6.815061<br>H 8.930188 1.819026 7.173681<br>H 3.989794 1.371758 7.201460<br>H 7.888148 -0.403069 7.599825<br>H 5.412277 -0.622414 7.610947<br>C 7.097891 5.441400 7.067312<br>C 6.231903 5.582812 8.160720<br>C 7.445266 6.575307 6.318472<br>C 5.714806 6.834709 8.489756<br>C 6.912342 7.819544 6.635086<br>C 6.045599 7.951358 7.722767<br>H 5.983958 4.719808 8.771809<br>H 8.135890 6.458422 5.488592<br>H 5.055476 6.937827 9.347553<br>H 7.175314 8.689913 6.039615<br>H 5.634162 8.925340 7.975296	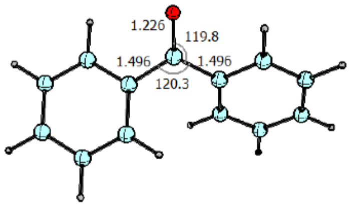
**TS5a**	U -0.680952 -0.011584 -0.284679<br>Si 5.242552 1.025732 -1.775799<br>Si 4.980934 -1.034122 1.616558<br>Si 1.598467 -2.711775 -1.893996<br>Si -4.550795 1.990555 -1.310538<br>Si -1.882893 4.209197 0.598021<br>Si 0.233929 2.097973 -4.180612<br>N -1.735605 -1.897347 -0.408172<br>N 0.809906 1.253621 1.622944<br>N 3.106366 3.657766 4.205601<br>C 3.833127 0.050986 -1.007409<br>C 3.748727 -0.706778 0.240726<br>C 2.509745 -1.336286 0.249910<br>H 2.168815 -2.013948 1.027880<br>C 1.733092 -1.042641 -0.955115<br>C 2.631983 -0.145345 -1.682443<br>H 2.412630 0.246788 -2.670814<br>C 5.020772 1.088988 -3.657526<br>H 5.064160 0.079794 -4.083955<br>H 5.831100 1.675449 -4.109292<br>H 4.075100 1.542236 -3.970043<br>C 5.324204 2.810437 -1.125005<br>H 4.391368 3.351722 -1.325073	

H	6.139700	3.367133	-1.604282
H	5.499144	2.831981	-0.042655
C	6.945157	0.234341	-1.509765
H	7.264017	0.216915	-0.463164
H	7.698787	0.798981	-2.074099
H	6.957650	-0.796533	-1.882713
C	5.877359	0.521799	2.231784
H	6.386387	1.068940	1.431664
H	6.631174	0.255399	2.983768
H	5.162240	1.206023	2.702672
C	6.270782	-2.337791	1.126209
H	5.774100	-3.272701	0.838889
H	6.936579	-2.559306	1.970550
H	6.892934	-2.021925	0.282934
C	4.055583	-1.744621	3.113703
H	3.249772	-1.079949	3.446178
H	4.746101	-1.883876	3.955159
H	3.617990	-2.723441	2.884520
C	0.304149	-2.765759	-3.272811
H	-0.719341	-2.684649	-2.895129
H	0.387450	-3.725746	-3.799572
H	0.468801	-1.975217	-4.011676
C	3.267984	-3.064244	-2.728358
H	3.420489	-2.444784	-3.619475
H	3.321073	-4.116781	-3.037421
H	4.098113	-2.868804	-2.039917
C	1.305510	-4.129289	-0.675078
H	2.243977	-4.403674	-0.178927
H	0.925077	-5.015531	-1.197395
H	0.577621	-3.858618	0.093855
C	-2.658426	1.941418	-1.294923
C	-1.690987	2.830288	-0.680539
C	-0.501299	2.731793	-1.451103
H	0.401539	3.303450	-1.261550
C	-0.667412	1.832839	-2.546381
C	-1.999529	1.328894	-2.404306
H	-2.486420	0.665768	-3.114613
C	-5.267898	0.328460	-1.843994
H	-4.896420	0.017726	-2.827123
H	-6.359562	0.411740	-1.920757
H	-5.042504	-0.467207	-1.127122
C	-5.391585	2.485102	0.305572
H	-5.169907	1.774919	1.106925
H	-6.477920	2.472330	0.146872
H	-5.124178	3.488735	0.648162
C	-5.029592	3.261326	-2.636522
H	-4.636460	4.258844	-2.411188
H	-6.121155	3.340824	-2.720048
H	-4.638407	2.967126	-3.617690
C	-3.044248	5.554256	-0.067558
H	-2.675323	5.936093	-1.027177
H	-3.076541	6.397353	0.634827
H	-4.072465	5.212743	-0.221013
C	-0.207364	5.058398	0.830841
H	0.563570	4.376693	1.204352
H	-0.310775	5.874270	1.557670

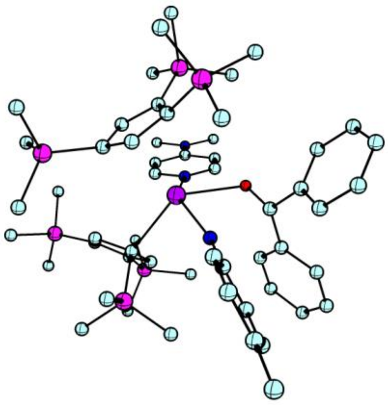

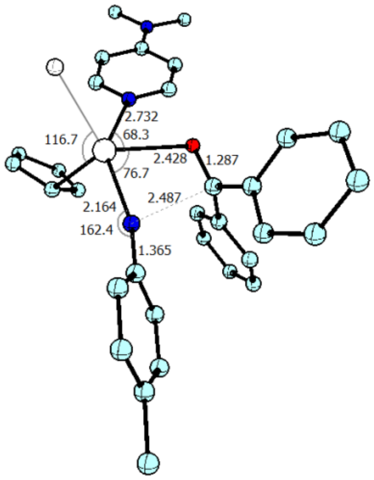

	H 0.154604 5.499579 -0.105526<br>C -2.491235 3.640562 2.296687<br>H -3.456432 3.128449 2.247029<br>H -2.605160 4.507239 2.960691<br>H -1.771649 2.957080 2.759446<br>C -0.984638 3.025692 -5.298267<br>H -1.852353 2.402589 -5.545924<br>H -0.504571 3.318703 -6.240725<br>H -1.356380 3.934525 -4.811116<br>C 1.711417 3.232307 -3.855490<br>H 1.370021 4.221755 -3.528807<br>H 2.306730 3.372251 -4.765810<br>H 2.373541 2.833951 -3.079460<br>C 0.758692 0.555763 -5.136394<br>H 1.541893 -0.026291 -4.641706<br>H 1.142342 0.859062 -6.119012<br>H -0.096046 -0.108038 -5.311003<br>C -2.407212 -2.983865 -0.889890<br>C -2.204975 -4.299333 -0.393146<br>H -1.490643 -4.448805 0.408092<br>C -2.899452 -5.389572 -0.905498<br>H -2.709147 -6.377908 -0.487553<br>C -3.827728 -5.255027 -1.946939<br>C -4.032420 -3.964457 -2.452177<br>H -4.744337 -3.816312 -3.263624<br>C -3.352040 -2.865026 -1.942788<br>H -3.534023 -1.875929 -2.351873<br>C -4.545667 -6.449321 -2.519046<br>H -5.482089 -6.158377 -3.008317<br>H -4.787275 -7.185447 -1.743111<br>H -3.935002 -6.966954 -3.272220<br>C 0.514044 1.383481 2.932079<br>H -0.361114 0.837894 3.267672<br>C 1.242002 2.146559 3.824085<br>H 0.921026 2.181808 4.858254<br>C 2.373363 2.870188 3.374941<br>C 2.691367 2.716267 2.004053<br>H 3.547722 3.209291 1.561572<br>C 1.908185 1.910449 1.200077<br>H 2.182885 1.769238 0.158547<br>C 4.239581 4.405308 3.688918<br>H 5.013071 3.739502 3.285663<br>H 4.683878 4.985406 4.498472<br>H 3.934430 5.102773 2.898065<br>C 2.740807 3.774228 5.605781<br>H 1.730475 4.186801 5.726720<br>H 3.441648 4.444935 6.103787<br>H 2.782360 2.802504 6.114622<br>O -1.662750 -0.249036 1.923731<br>C -2.302874 -1.365326 1.953946<br>C -1.633650 -2.544645 2.557486<br>C -2.337019 -3.693862 2.956745<br>C -0.259598 -2.470266 2.851324<br>C -1.679978 -4.746792 3.590012<br>C 0.391359 -3.516902 3.489943<br>C -0.315107 -4.666256 3.855434	

<table>
<tr><td></td><td>H -3.405688 -3.763180 2.794147
H 0.293973 -1.574942 2.585977
H -2.243554 -5.627587 3.886029
H 1.451550 -3.434870 3.711619
H 0.195643 -5.485685 4.354399
C -3.793311 -1.298412 1.953904
C -4.364046 -0.250665 2.695391
C -4.637900 -2.259513 1.378069
C -5.742225 -0.183832 2.882764
C -6.018886 -2.165633 1.537676
C -6.575390 -1.138684 2.300165
H -3.712463 0.492431 3.144358
H -4.219149 -3.065140 0.786202
H -6.165583 0.618780 3.481225
H -6.660766 -2.906141 1.067856
H -7.651901 -1.082881 2.439635</td><td></td></tr>
<tr><td>INT5</td><td>U -0.370420 0.043214 -0.278487
Si 5.359286 0.876617 -1.783607
Si 5.053386 -1.033638 1.693777
Si 1.777548 -2.916047 -1.877857
Si -4.384963 1.629916 -1.356449
Si -1.945977 3.976266 0.684000
Si 0.468808 2.247996 -4.163259
N -1.842943 -1.769054 0.151961
N 1.064318 1.385996 1.414034
N 3.318125 3.739779 4.062943
C 3.943744 -0.082015 -1.000142
C 3.849857 -0.792284 0.271118
C 2.632940 -1.464847 0.271390
H 2.310355 -2.141101 1.056353
C 1.876529 -1.238151 -0.960412
C 2.767743 -0.342690 -1.700418
H 2.565971 0.002000 -2.710379
C 5.134102 0.898115 -3.665387
H 5.165807 -0.120656 -4.069675
H 5.949319 1.465476 -4.132192
H 4.192703 1.355185 -3.986020
C 5.456356 2.674324 -1.174252
H 4.541446 3.231174 -1.410861
H 6.293883 3.200180 -1.650195
H 5.608109 2.719888 -0.089684
C 7.052239 0.075521 -1.492212
H 7.370466 0.090510 -0.445312
H 7.812093 0.612690 -2.074611
H 7.053469 -0.967582 -1.829441
C 5.929679 0.560714 2.231685
H 6.502784 1.029055 1.425079
H 6.626511 0.348188 3.052600
H 5.199490 1.293774 2.593031
C 6.360347 -2.351152 1.295328
H 5.878200 -3.311720 1.075872
H 7.028469 -2.502729 2.153120
H 6.978917 -2.087581 0.431515
C 4.098779 -1.664281 3.205739
H 3.286639 -0.985786 3.490447
H 4.775175 -1.759272 4.064699
H 3.662613 -2.653660 3.025127
C 0.784690 -2.834676 -3.486995
H -0.285756 -2.676480 -3.321509
H 0.888753 -3.792214 -4.014420</td><td>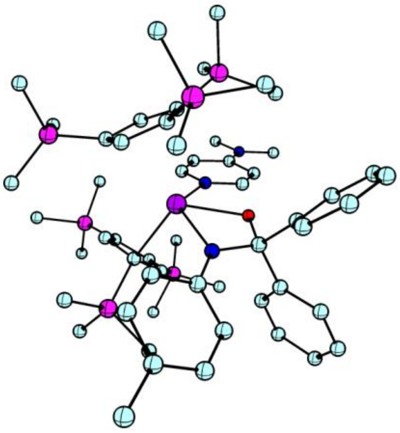
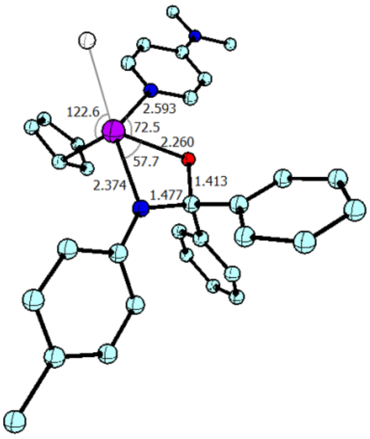
</td></tr>
</table>

	H	1.155981	-2.052355	-4.157535	
	C	3.537129	-3.430598	-2.367399	
	H	3.895476	-2.869199	-3.237434	
	H	3.561517	-4.498016	-2.622388	
	H	4.242700	-3.260748	-1.546771	
	C	1.081744	-4.289058	-0.781669	
	H	1.742236	-4.487907	0.070287	
	H	1.014504	-5.214679	-1.368154	
	H	0.082260	-4.075215	-0.391449	
	C	-2.495291	1.772064	-1.356179	
	C	-1.611105	2.684091	-0.660775	
	C	-0.402454	2.732757	-1.419461	
	H	0.441892	3.370836	-1.178914	
	C	-0.485836	1.909526	-2.577070	
	C	-1.771459	1.294156	-2.491620	
	H	-2.192521	0.644273	-3.255020	
	C	-4.926973	-0.022119	-2.091727	
	H	-4.560966	-0.160806	-3.115872	
	H	-6.023226	-0.056279	-2.133204	
	H	-4.586697	-0.873619	-1.495861	
	C	-5.212658	1.847992	0.322117	
	H	-4.846467	1.120095	1.051677	
	H	-6.291776	1.684536	0.206960	
	H	-5.077010	2.850411	0.738950	
	C	-5.017541	2.978613	-2.533669	
	H	-4.730699	3.984766	-2.210463	
	H	-6.112921	2.945716	-2.597968	
	H	-4.619331	2.829259	-3.544451	
	C	-3.264290	5.200110	0.083602	
	H	-2.979547	5.630761	-0.883967	
	H	-3.352644	6.025542	0.801932	
	H	-4.257037	4.754784	-0.029644	
	C	-0.379936	5.012156	0.933205	
	H	0.480474	4.418738	1.259219	
	H	-0.572436	5.767009	1.706384	
	H	-0.096936	5.546703	0.018499	
	C	-2.453985	3.274323	2.362627	
	H	-3.282380	2.564103	2.293270	
	H	-2.762266	4.092005	3.027138	
	H	-1.615086	2.755971	2.839023	
	C	-0.683797	3.271760	-5.264792	
	H	-1.573167	2.697534	-5.550914	
	H	-0.174510	3.580001	-6.186769	
	H	-1.026049	4.176419	-4.748954	
	C	1.984640	3.296089	-3.745092	
	H	1.684227	4.268733	-3.337673	
	H	2.588595	3.486636	-4.640362	
	H	2.628226	2.805699	-3.006904	
	C	0.951823	0.715404	-5.157752	
	H	1.751749	0.130719	-4.693285	
	H	1.303834	1.022712	-6.150639	
	H	0.093359	0.049963	-5.307488	
	C	-2.370286	-2.832087	-0.575012	
	C	-2.727975	-4.095362	-0.053157	
	H	-2.618299	-4.293262	1.005157	
	C	-3.219844	-5.107019	-0.874359	
	H	-3.483493	-6.060343	-0.417858	
	C	-3.379892	-4.942216	-2.253559	
	C	-3.033376	-3.691971	-2.777667	
	H	-3.158600	-3.503438	-3.842969	
	C	-2.552431	-2.670509	-1.966619	
	H	-2.343683	-1.698358	-2.409090	

	C -3.881159 -6.058354 -3.132412<br>H -4.386345 -5.670803 -4.024431<br>H -4.590105 -6.702152 -2.599333<br>H -3.060483 -6.701599 -3.479757<br>C 0.821643 1.388794 2.742750<br>H 0.005385 0.746768 3.059015<br>C 1.541264 2.135485 3.651978<br>H 1.271610 2.071111 4.699025<br>C 2.601534 2.965575 3.210244<br>C 2.868496 2.930959 1.818386<br>H 3.672703 3.507906 1.379824<br>C 2.098312 2.141859 0.990530<br>H 2.327175 2.094511 -0.070109<br>C 4.396361 4.574332 3.559878<br>H 5.187722 3.973345 3.094399<br>H 4.836906 5.126942 4.390074<br>H 4.030560 5.301301 2.823737<br>C 3.011828 3.735209 5.483519<br>H 1.980774 4.059765 5.672467<br>H 3.682398 4.426167 5.994968<br>H 3.150227 2.739106 5.922745<br>O -1.160782 -0.407425 1.790439<br>C -1.991015 -1.535658 1.602463<br>C -1.456400 -2.668204 2.522305<br>C -2.291647 -3.494737 3.284762<br>C -0.076522 -2.851912 2.638006<br>C -1.763656 -4.489664 4.109094<br>C 0.458010 -3.841296 3.459880<br>C -0.385412 -4.670598 4.199099<br>H -3.368851 -3.366903 3.237471<br>H 0.584246 -2.188541 2.092006<br>H -2.436782 -5.118456 4.687277<br>H 1.536600 -3.958263 3.531814<br>H 0.028342 -5.441956 4.844015<br>C -3.421222 -1.183513 2.053873<br>C -3.569564 -0.388804 3.198828<br>C -4.574736 -1.688912 1.446036<br>C -4.829355 -0.113212 3.725867<br>C -5.839194 -1.420477 1.977390<br>C -5.973227 -0.636041 3.120265<br>H -2.679531 0.009010 3.675421<br>H -4.496830 -2.310824 0.561751<br>H -4.917949 0.507561 4.614644<br>H -6.720822 -1.828916 1.488954<br>H -6.957509 -0.428787 3.533091	
**TS5b**	U 9.463702 2.537629 6.297875<br>Si 14.071557 3.079341 5.410139<br>Si 12.831961 0.966352 8.542927<br>Si 10.255540 -0.643561 3.606010<br>Si 5.373662 3.417220 4.884488<br>Si 7.137742 6.192556 7.091512<br>Si 10.205059 5.011760 2.525054<br>N 6.426610 -0.170563 7.034896<br>N 10.334779 4.054778 8.173570<br>N 11.637880 6.502626 11.328251<br>C 12.461664 2.127189 5.720012<br>C 12.085368 1.255568 6.825118<br>C 11.160172 0.311298 6.303362<br>H 10.726982 -0.497277 6.880119<br>C 10.888296 0.565589 4.920959	

C	11.701809	1.701558	4.602051
H	11.781598	2.130315	3.610910
C	14.269804	3.348733	3.546765
H	14.309270	2.392285	3.012492
H	15.217579	3.869256	3.359188
H	13.474476	3.951980	3.099854
C	14.289843	4.769089	6.244716
H	13.605674	5.523412	5.837814
H	15.309455	5.123813	6.045882
H	14.158776	4.734325	7.330736
C	15.538585	1.986458	5.902766
H	15.610524	1.798060	6.977576
H	16.472892	2.468803	5.587398
H	15.479351	1.015535	5.397038
C	13.763360	2.450638	9.260256
H	14.608730	2.782181	8.650540
H	14.161562	2.165461	10.242800
H	13.099757	3.308770	9.411544
C	14.012391	-0.512110	8.427156
H	13.464481	-1.413987	8.129196
H	14.478113	-0.712505	9.400536
H	14.812469	-0.353922	7.696383
C	11.479383	0.521140	9.779308
H	10.812846	1.369758	9.965962
H	11.932326	0.235075	10.737221
H	10.860840	-0.315051	9.439248
C	8.772691	0.002723	2.628836
H	7.898053	0.160385	3.268951
H	8.490113	-0.727713	1.860211
H	9.000585	0.945368	2.118585
C	11.691554	-0.887736	2.394226
H	11.970839	0.050382	1.900423
H	11.417295	-1.606366	1.611676
H	12.581064	-1.272367	2.907070
C	9.820519	-2.314317	4.363909
H	10.587943	-2.655599	5.068084
H	9.752975	-3.062746	3.564035
H	8.853377	-2.307614	4.877992
C	7.196894	3.910615	5.084487
C	7.846057	4.948225	5.851373
C	9.090913	5.220031	5.199194
H	9.782851	5.997557	5.508855
C	9.246798	4.421572	4.034384
C	8.089301	3.581670	4.014965
H	7.854588	2.874033	3.224681
C	5.257261	1.633033	4.310021
H	5.839959	1.439375	3.401596
H	4.213412	1.375413	4.089797
H	5.608915	0.971897	5.113385
C	4.289652	3.608710	6.414160
H	4.648540	2.983440	7.236607
H	3.278554	3.265274	6.158372
H	4.202559	4.637733	6.775031
C	4.694581	4.545734	3.515585
H	4.756811	5.607239	3.779936
H	3.641156	4.310381	3.316027

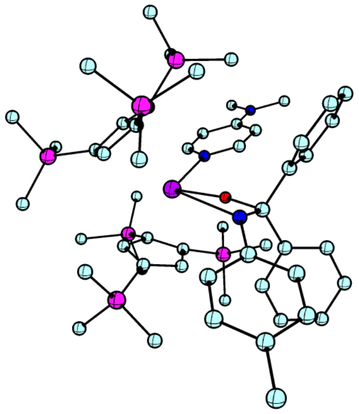

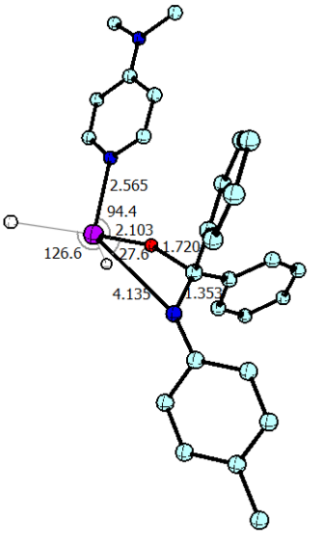

	H 5.250707 4.403850 2.581051<br>C 5.762606 7.186394 6.245469<br>H 6.127573 7.636213 5.314331<br>H 5.436032 8.001357 6.904469<br>H 4.881554 6.586125 6.000998<br>C 8.481392 7.460053 7.515654<br>H 9.359710 7.008791 7.988633<br>H 8.069899 8.196303 8.217981<br>H 8.817838 8.008613 6.627823<br>C 6.492568 5.466529 8.711877<br>H 5.908822 4.552650 8.574029<br>H 5.850802 6.201064 9.215601<br>H 7.316161 5.229999 9.393960<br>C 8.975524 6.038792 1.512743<br>H 8.149589 5.419112 1.144038<br>H 9.466210 6.495056 0.643512<br>H 8.540383 6.843369 2.116852<br>C 11.601716 6.168559 3.064745<br>H 11.189121 7.081128 3.511851<br>H 12.203910 6.472866 2.200126<br>H 12.277307 5.713935 3.796082<br>C 10.828297 3.647604 1.372741<br>H 11.680352 3.081864 1.760782<br>H 11.142985 4.098396 0.422816<br>H 10.027746 2.935133 1.140988<br>C 6.047082 -1.452993 6.737381<br>C 5.612944 -2.412259 7.692466<br>H 5.633212 -2.151150 8.746962<br>C 5.139593 -3.664473 7.315619<br>H 4.816853 -4.357625 8.093344<br>C 5.048987 -4.055676 5.974291<br>C 5.452416 -3.112560 5.020013<br>H 5.389532 -3.365975 3.961212<br>C 5.934851 -1.860005 5.381748<br>H 6.229342 -1.155508 4.608426<br>C 4.563219 -5.426381 5.577948<br>H 4.007827 -5.401595 4.632137<br>H 3.899649 -5.852430 6.340006<br>H 5.391749 -6.137448 5.442061<br>C 9.820190 3.948682 9.417880<br>H 9.042535 3.204545 9.545650<br>C 10.214265 4.727203 10.484805<br>H 9.738091 4.565252 11.443720<br>C 11.215410 5.716115 10.311428<br>C 11.741853 5.829371 8.998102<br>H 12.506403 6.556559 8.754444<br>C 11.280661 5.000286 7.998518<br>H 11.686076 5.091157 6.997771<br>C 12.653601 7.516546 11.093432<br>H 13.591819 7.068774 10.743150<br>H 12.857348 8.040242 12.027436<br>H 12.319479 8.255634 10.354488<br>C 11.058570 6.354207 12.654173<br>H 9.982951 6.571399 12.649742<br>H 11.542997 7.053977 13.335105<br>H 11.210764 5.340628 13.044518				

	O 8.298246 1.347478 7.581721<br>C 7.132067 0.206938 8.125949<br>C 8.002917 -0.864690 8.789974<br>C 7.968567 -1.179115 10.154191<br>C 8.798471 -1.669514 7.962663<br>C 8.722265 -2.235781 10.672509<br>C 9.552622 -2.721691 8.471212<br>C 9.523648 -3.008832 9.837880<br>H 7.329090 -0.617848 10.826989<br>H 8.789307 -1.472264 6.897751<br>H 8.669299 -2.456967 11.736334<br>H 10.150617 -3.331050 7.797414<br>H 10.107100 -3.833321 10.240540<br>C 6.382726 1.083460 9.127872<br>C 7.006264 1.721076 10.209240<br>C 4.990995 1.156753 9.036762<br>C 6.266081 2.421733 11.158674<br>C 4.242375 1.865005 9.979616<br>C 4.876055 2.501657 11.044454<br>H 8.082194 1.625811 10.323990<br>H 4.513870 0.640409 8.208786<br>H 6.770926 2.899128 11.996181<br>H 3.159821 1.909743 9.884148<br>H 4.295247 3.046031 11.785312	
**5**	U 6.650204 16.809966 4.699887<br>Si 10.800925 16.720879 4.253365<br>Si 8.828948 13.278501 4.513175<br>Si 6.562856 16.533691 0.280039<br>Si 2.665749 18.688062 5.108848<br>Si 5.155349 18.391011 8.228124<br>Si 7.646715 21.138690 3.849132<br>O 7.078392 15.880492 6.433610<br>N 4.647183 15.171072 4.498137<br>N 1.540715 12.345574 4.443685<br>C 9.157773 16.150298 3.508713<br>C 8.459894 14.883959 3.588647<br>C 7.418171 14.937698 2.621940<br>H 6.731837 14.120744 2.420766<br>C 7.446830 16.168476 1.894257<br>C 8.517112 16.901945 2.469144<br>H 8.843693 17.881298 2.136313<br>C 11.432302 18.171594 3.208722<br>H 11.604582 17.863246 2.170589<br>H 12.387642 18.531090 3.611325<br>H 10.744703 19.024189 3.193040<br>C 10.687084 17.283282 6.057422<br>H 10.059549 18.176194 6.168893<br>H 11.686229 17.536435 6.435166<br>H 10.264962 16.506661 6.703841<br>C 12.127287 15.375650 4.128379<br>H 11.925330 14.514099 4.771890<br>H 13.096692 15.792775 4.430602<br>H 12.225823 15.012978 3.098653<br>C 10.107191 12.271292 3.532956<br>H 9.745847 12.078583 2.515144<br>H 10.275836 11.299825 4.015979	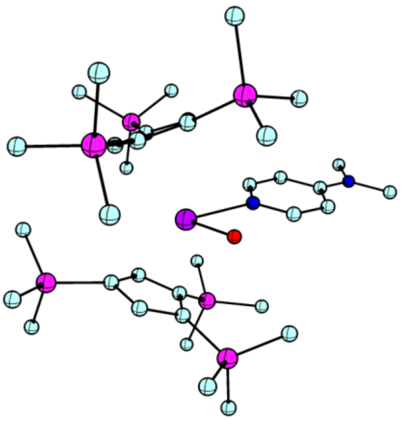<br>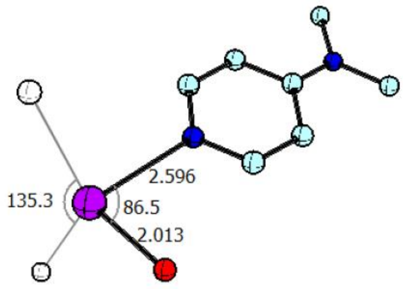

	H 11.075278 12.775168 3.450838<br>C 7.257580 12.218621 4.534686<br>H 6.437461 12.713067 5.064577<br>H 7.463033 11.270402 5.047921<br>H 6.911388 11.973103 3.523578<br>C 9.389380 13.534241 6.294025<br>H 10.378732 13.997592 6.374737<br>H 9.430827 12.572405 6.821283<br>H 8.660683 14.188570 6.788724<br>C 5.761843 14.936767 -0.349120<br>H 6.513166 14.151296 -0.491555<br>H 5.270953 15.108871 -1.315032<br>H 5.003516 14.546440 0.340369<br>C 5.225079 17.870224 0.408529<br>H 4.417193 17.597947 1.097764<br>H 4.771562 18.048793 -0.574878<br>H 5.647543 18.820621 0.754003<br>C 7.841859 17.121070 -0.984555<br>H 8.329911 18.047253 -0.658201<br>H 7.374353 17.317589 -1.957519<br>H 8.624727 16.368003 -1.131188<br>C 4.543080 18.798681 5.241798<br>C 5.420598 18.787866 6.398241<br>C 6.644307 19.374271 5.975773<br>H 7.500707 19.536162 6.623374<br>C 6.572451 19.796367 4.607822<br>C 5.272705 19.416831 4.179430<br>H 4.868370 19.611116 3.189566<br>C 2.159081 18.341690 3.315261<br>H 2.594709 19.060178 2.611757<br>H 1.068026 18.414527 3.221315<br>H 2.448573 17.334676 2.996417<br>C 1.839753 17.377846 6.194240<br>H 2.199169 16.367950 5.968798<br>H 0.757009 17.395150 6.013804<br>H 1.995057 17.559865 7.262384<br>C 1.948133 20.383044 5.564462<br>H 2.204871 20.673523 6.588845<br>H 0.853948 20.382739 5.477830<br>H 2.338289 21.159002 4.895120<br>C 4.668926 16.607709 8.613921<br>H 3.742297 16.293998 8.122404<br>H 4.533890 16.482011 9.696107<br>H 5.482678 15.955573 8.277153<br>C 3.847927 19.564532 8.950567<br>H 4.109632 20.612618 8.761810<br>H 3.780775 19.427380 10.037703<br>H 2.848474 19.391368 8.536402<br>C 6.779772 18.731630 9.137116<br>H 7.582645 18.089732 8.757594<br>H 6.657254 18.513511 10.205592<br>H 7.098582 19.776761 9.047614<br>C 6.691803 22.768566 4.009019<br>H 5.754149 22.736603 3.441286<br>H 7.283698 23.612662 3.632637<br>H 6.437401 22.974442 5.055379	

	C 9.257477 21.309767 4.824570<br>H 9.884763 22.097878 4.390109<br>H 9.847081 20.387127 4.837706<br>H 9.053750 21.589372 5.865114<br>C 8.009273 20.888600 2.008191<br>H 7.085689 20.728756 1.439015<br>H 8.672918 20.039461 1.815440<br>H 8.493177 21.784480 1.599059<br>C 3.894967 14.959934 3.400130<br>H 4.143459 15.554222 2.525401<br>C 2.864305 14.047261 3.328758<br>H 2.318914 13.950826 2.398049<br>C 2.544669 13.256685 4.462512<br>C 3.345277 13.477131 5.610844<br>H 3.190936 12.916103 6.524280<br>C 4.357501 14.414525 5.579589<br>H 4.993263 14.585038 6.442893<br>C 0.756752 12.150140 3.235969<br>H 0.003599 11.383435 3.418913<br>H 1.383554 11.817383 2.398705<br>H 0.238637 13.071282 2.940369<br>C 1.251964 11.555157 5.628998<br>H 2.110176 10.935386 5.918263<br>H 0.411156 10.893238 5.420393<br>H 0.979670 12.193367 6.478935	
**Ph$_2$CN(*p*-totyl)**	N -5.331834 -4.555083 1.793001<br>C -5.110301 -5.514875 0.796995<br>C -5.092225 -6.895575 1.049203<br>H -5.185189 -7.254551 2.070018<br>C -4.972069 -7.801904 -0.000210<br>H -4.966862 -8.867816 0.220500<br>C -4.852993 -7.375505 -1.327820<br>C -4.876640 -5.995850 -1.570969<br>H -4.792184 -5.631453 -2.593190<br>C -5.025214 -5.080520 -0.535090<br>H -5.065918 -4.013857 -0.739859<br>C -4.743098 -8.363430 -2.459930<br>H -4.329396 -9.318704 -2.119600<br>H -4.101371 -7.984035 -3.262959<br>H -5.725590 -8.573775 -2.903972<br>C -4.676795 -4.485879 2.898271<br>C -3.469569 -5.305311 3.233338<br>C -3.415874 -6.046391 4.422064<br>C -2.372445 -5.338550 2.362466<br>C -2.296771 -6.820501 4.722912<br>C -1.247186 -6.097871 2.673271<br>C -1.208404 -6.845236 3.850923<br>H -4.259082 -6.022291 5.107839<br>H -2.402940 -4.763771 1.441253<br>H -2.273694 -7.400576 5.641939<br>H -0.399772 -6.107635 1.992709<br>H -0.332035 -7.442499 4.089190<br>C -5.144097 -3.488863 3.901058<br>C -4.296323 -3.001045 4.906484<br>C -6.457341 -2.996546 3.828444<br>C -4.749978 -2.045393 5.814280	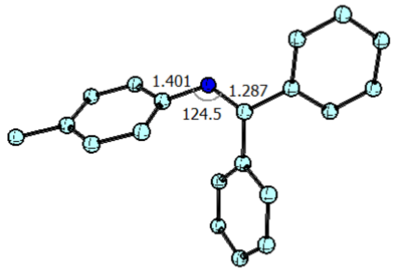

	C -6.910673 -2.049557 4.739045<br>C -6.057857 -1.569977 5.736584<br>H -3.273277 -3.359175 4.971065<br>H -7.107938 -3.373456 3.045409<br>H -4.077755 -1.671178 6.582168<br>H -7.932779 -1.684850 4.674913<br>H -6.413057 -0.829845 6.449247	

Table S5. Frequencies of the stationary points optimized for **4**+Ph_2CO, obtained with B3PW91-PCM/ECP80MWB method.

Species	Frequencies (cm^{-1})
**4**	22 22 25 28 30 32 39 42 44 47 48 54 55 57 59 61 64 67<br>70 74 79 82 83 84 91 96 100 103 105 109 115 120 123 124 125 127 135<br>135 137 139 141 142 145 148 149 151 156 163 164 167 168 169 172 173 176 177<br>180 185 185 186 187 190 195 200 204 205 207 209 210 211 216 218 220 222 225<br>227 228 230 237 245 259 264 264 266 270 271 281 283 293 295 299 327 352 357<br>360 366 372 374 378 406 425 428 430 430 490 498 507 508 534 547 565 567 621<br>622 623 624 637 638 646 648 653 656 659 676 683 684 685 686 687 688 688 690<br>692 693 693 693 693 696 697 699 700 710 725 749 774 775 776 777 778 779 779<br>780 781 782 785 786 789 797 829 832 836 841 842 848 862 863 865 866 866 868<br>869 869 871 871 872 873 874 875 876 881 882 887 892 893 935 943 944 948 952<br>976 983 1004 1006 1007 1010 1022 1028 1066 1092 1100<br>1123 1124 1135 1147 1149 1151 1180 1195 1202 1217<br>1240 1248 1255 1265 1279 1289 1291 1296 1298 1304<br>1305 1306 1306 1306 1307 1308 1309 1309 1310 1311<br>1313 1314 1318 1318 1319 1321 1323 1324 1345 1373<br>1378 1386 1390 1408 1432 1437 1460 1466 1472 1473<br>1475 1476 1476 1476 1477 1477 1478 1478 1479 1480<br>1480 1481 1482 1483 1485 1486 1486 1487 1487 1488<br>1488 1488 1489 1491 1492 1493 1495 1495 1495 1496<br>1498 1498 1499 1501 1504 1505 1505 1506 1507 1507<br>1515 1516 1533 1542 1548 1588 1595 1596 1669 1687<br>3034 3043 3046 3047 3047 3049 3049 3050 3051 3052<br>3053 3054 3054 3055 3055 3056 3056 3056 3057 3057<br>3058 3089 3105 3107 3124 3125 3126 3126 3126 3127<br>3128 3128 3129 3129 3131 3131 3131 3132 3132 3133<br>3134 3134 3134 3135 3136 3136 3139 3146 3148 3148<br>3149 3150 3150 3151 3152 3152 3153 3156 3161 3161<br>3163 3164 3176 3179 3189 3204 3209 3218 3219 3234<br>3240 3241 3244 3258 3260
**Ph_2CO**	41 63 94 136 213 234 290 377 410 422 445 448 572 626 629 649 697 710<br>716 734 780 832 866 870 939 954 961 985 989 1010 1011 1016 1017<br>1059 1062 1113 1117 1179 1191 1192 1205 1212 1309<br>1349 1350 1383 1384 1493 1493 1536 1541 1646 1647<br>1667 1668 1749 3200 3200 3209 3210 3218 3220 3227<br>3228 3232 3233
**TS5a**	-104 12 20 21 25 28 30 33 36 38 40 43 44 46 49 54 56 59<br>62 63 68 70 71 74 76 80 84 84 86 88 91 94 97 101 102 106 110<br>112 113 118 119 122 127 128 135 135 137 138 142 146 150 150 153 156 162 163<br>166 171 172 174 175 177 179 181 183 186 187 191 192 193 196 199 200 200 203<br>208 209 211 214 216 219 221 221 223 227 229 233 235 237 240 251 254 259 259<br>264 267 269 276 283 289 292 303 308 321 338 343 351 366 371 372 376 387 399<br>418 422 426 428 428 429 434 456 469 474 483 489 508 537 548 553 562 578 619<br>623 623 624 626 628 635 637 644 644 652 658 662 669 678 679 682 682 682 686<br>687 688 690 692 695 695 696 697 699 700 703 704 709 714 715 731 738 740 750<br>775 775 776 777 777 778 779 781 782 782 783 787 788 789 792 800 831 834 838<br>839 849 852 860 862 863 865 867 868 869 870 872 873 873 873 874 875 876 877<br>879 880 882 883 887 891 905 920 937 942 953 956 959 966 979 983 990 996 1001<br>1002 1009 1011 1011 1015 1015 1021 1024 1031 1051<br>1061 1065 1070 1091 1095 1116 1123 1138 1138 1148<br>1150 1151 1177 1189 1190 1191 1195 1204 1212 1214<br>1219 1235 1239 1242 1250 1260 1285 1289 1295 1298<br>1301 1302 1303 1304 1305 1306 1306 1308 1309 1310<br>1310 1311 1313 1314 1314 1314 1319 1320 1320 1324<br>1343 1346 1346 1364 1368 1378 1385 1393 1401 1432

	1432 1445 1458 1462 1464 1474 1474 1475 1476 1477<br>1477 1478 1478 1480 1480 1481 1482 1483 1484 1485<br>1485 1486 1486 1487 1487 1487 1488 1489 1489 1490<br>1491 1492 1492 1493 1493 1495 1495 1496 1497 1500<br>1501 1502 1503 1503 1504 1504 1505 1507 1507 1512<br>1514 1532 1536 1541 1546 1549 1588 1596 1597 1641<br>1646 1663 1664 1666 1683 3038 3039 3044 3046 3046<br>3048 3048 3049 3050 3051 3052 3052 3054 3054 3055<br>3055 3056 3057 3058 3058 3061 3098 3099 3102 3124<br>3124 3124 3126 3127 3128 3128 3128 3128 3129 3129<br>3130 3130 3130 3131 3133 3134 3137 3138 3138 3138<br>3141 3141 3142 3146 3149 3151 3153 3153 3156 3156<br>3157 3157 3164 3169 3170 3171 3173 3173 3176 3185<br>3200 3203 3210 3215 3217 3219 3222 3224 3228 3230<br>3231 3233 3234 3235 3242 3242 3248 3252 3255 3260
**INT5**	17 21 24 29 31 33 37 39 40 43 48 50 51 52 54 58 59 62<br>64 65 66 68 73 77 80 80 82 83 85 87 91 94 97 98 104 105 108<br>112 118 121 123 125 129 133 135 138 139 141 142 144 146 151 152 153 159 163<br>165 169 172 174 177 178 179 179 180 183 184 190 191 192 192 193 195 196 198<br>200 206 211 215 216 219 222 225 226 227 230 232 236 243 244 252 257 262 264<br>265 272 274 286 289 295 298 304 309 334 349 356 359 366 371 374 402 407 414<br>418 421 424 427 433 434 457 479 485 491 506 514 543 550 567 586 612 620 621<br>623 624 630 631 635 637 646 646 654 657 669 676 682 683 683 684 684 687 687<br>687 688 690 691 693 694 695 697 699 703 706 708 716 718 724 740 745 750 774<br>775 776 777 778 779 780 781 782 782 783 784 786 788 796 807 818 834 840 843<br>856 860 861 862 864 865 865 866 867 868 869 870 871 873 874 874 874 876 878<br>879 882 885 888 893 907 917 922 940 946 954 957 970 979 981 983 987 996 997<br>999 999 1014 1016 1020 1022 1027 1036 1042 1050 1062<br>1068 1068 1087 1091 1101 1118 1120 1131 1149 1150<br>1150 1155 1183 1187 1187 1189 1196 1210 1214 1215<br>1219 1227 1229 1234 1239 1254 1271 1289 1293 1295<br>1299 1301 1303 1304 1304 1306 1306 1308 1311 1311<br>1312 1312 1314 1315 1319 1320 1323 1323 1324 1326<br>1340 1346 1356 1363 1373 1377 1384 1389 1395 1435<br>1436 1457 1461 1466 1472 1473 1474 1474 1475 1475<br>1477 1478 1479 1480 1480 1481 1481 1481 1482 1482<br>1484 1485 1486 1486 1486 1487 1488 1489 1489 1490<br>1490 1491 1492 1492 1492 1494 1495 1496 1497 1498<br>1500 1501 1502 1503 1504 1505 1507 1508 1515 1515<br>1533 1540 1547 1547 1553 1589 1594 1615 1649 1651<br>1667 1670 1678 1688 3039 3043 3046 3048 3049 3049<br>3049 3050 3051 3051 3052 3053 3053 3055 3055 3055<br>3055 3056 3056 3058 3063 3102 3105 3106 3124 3125<br>3125 3125 3127 3128 3128 3128 3128 3129 3129 3130<br>3131 3131 3132 3132 3134 3134 3137 3137 3140 3142<br>3145 3146 3147 3149 3150 3151 3152 3153 3156 3156<br>3159 3159 3166 3169 3169 3174 3177 3178 3188 3189<br>3190 3191 3200 3201 3212 3215 3215 3222 3225 3232<br>3234 3235 3236 3239 3248 3255 3260 3263 3264
**TS5b**	-270 10 16 21 24 25 29 31 32 38 41 45 48 51 51 53 57 59<br>63 66 67 70 70 73 75 77 81 87 90 92 95 96 99 102 106 110 113<br>119 120 126 128 128 131 135 137 138 140 141 144 146 149 152 153 155 161 162<br>165 166 169 171 175 179 181 183 185 187 187 188 194 195 196 199 200 205 207<br>211 215 216 219 222 223 225 228 232 232 234 237 242 248 257 259 263 264 269<br>270 271 276 282 286 290 295 298 302 305 317 323 355 358 363 368 375 376 379<br>405 421 427 428 429 431 435 439 487 489 501 504 509 529 545 549 566 600 620

	624 624 625 629 631 637 640 644 650 650 654 655 658 675 680 684 684 685 687<br>689 690 691 691 693 693 695 696 698 700 702 702 703 712 715 718 726 729 748<br>773 773 776 777 778 778 780 782 783 784 786 786 787 790 798 813 825 830 835<br>844 855 858 862 864 864 866 868 868 870 871 871 872 872 874 875 876 876 877<br>878 880 881 885 892 895 905 933 939 942 943 944 949 962 980 981 988 995 997<br>999 999 1004 1006 1008 1017 1021 1023 1030 1059 1065<br>1066 1090 1099 1103 1111 1125 1129 1142 1146 1146<br>1150 1156 1182 1184 1186 1195 1205 1209 1213 1214<br>1230 1238 1251 1251 1258 1270 1277 1284 1287 1295<br>1301 1303 1304 1306 1308 1308 1309 1310 1310 1312<br>1314 1315 1316 1318 1320 1323 1323 1324 1325 1332<br>1332 1348 1349 1375 1378 1381 1392 1392 1409 1429<br>1441 1442 1465 1465 1472 1473 1474 1475 1476 1476<br>1477 1478 1478 1479 1479 1480 1481 1481 1482 1484<br>1486 1486 1486 1487 1487 1488 1488 1490 1490 1492<br>1492 1492 1493 1494 1494 1495 1497 1498 1499 1499<br>1500 1501 1502 1504 1505 1507 1510 1516 1516 1520<br>1533 1533 1538 1547 1557 1591 1599 1600 1645 1649<br>1665 1667 1676 1691 3025 3032 3045 3048 3048 3049<br>3051 3051 3052 3052 3053 3053 3054 3055 3056 3057<br>3058 3058 3058 3059 3059 3086 3109 3111 3118 3121<br>3124 3126 3126 3128 3129 3129 3129 3130 3130 3130<br>3131 3132 3132 3133 3134 3135 3135 3137 3137 3138<br>3141 3147 3148 3149 3150 3151 3152 3156 3157 3158<br>3158 3159 3163 3164 3167 3171 3172 3180 3183 3187<br>3192 3195 3197 3207 3209 3210 3213 3219 3224 3225<br>3228 3231 3251 3254 3257 3258 3263 3268 3270
**5**	16 21 24 31 34 36 41 45 52 53 54 58 61 63 66 67 73 80 81<br>83 87 95 99 101 109 110 119 124 126 127 132 134 138 140 141 145 146 148<br>151 153 154 156 159 164 166 167 169 170 174 176 176 179 179 183 187 187 195<br>198 199 202 204 207 209 211 212 217 219 219 223 224 226 228 234 237 250 264<br>264 265 272 275 283 286 293 295 299 352 361 367 376 380 386 406 426 433 436<br>488 500 508 552 564 623 623 623 624 636 638 644 648 656 661 675 680 683 685<br>686 687 688 689 689 690 690 691 692 694 695 696 699 707 710 729 751 773 775<br>777 778 778 779 779 780 781 781 783 790 795 834 841 845 850 862 863 864 866<br>867 868 869 871 871 872 873 873 874 876 877 879 885 888 894 899 949 952 980<br>989 1006 1008 1016 1025 1090 1100 1121 1131 1148<br>1148 1151 1182 1190 1215 1247 1250 1267 1289 1289<br>1289 1296 1301 1302 1303 1304 1305 1306 1306 1308<br>1309 1310 1311 1312 1312 1315 1317 1319 1320 1321<br>1323 1372 1386 1393 1401 1434 1465 1473 1474 1474<br>1475 1476 1476 1477 1478 1478 1478 1480 1480 1481<br>1481 1482 1483 1483 1485 1485 1486 1487 1489 1489<br>1490 1491 1491 1492 1493 1493 1494 1496 1497 1498<br>1499 1500 1501 1503 1505 1506 1508 1515 1518 1533<br>1547 1589 1594 1687 3039 3042 3047 3047 3047 3048<br>3048 3049 3050 3050 3051 3051 3052 3052 3052 3054<br>3054 3054 3056 3056 3103 3107 3121 3122 3125 3126<br>3126 3127 3128 3128 3129 3129 3129 3130 3131 3131<br>3131 3132 3132 3133 3136 3136 3138 3139 3139 3141<br>3141 3146 3146 3146 3147 3148 3148 3150 3152 3152<br>3155 3157 3179 3189 3216 3218 3227 3236 3239 3241<br>3258 3260
**Ph_2CN(*p*-totyl)**	35 39 41 45 61 64 80 137 143 210 219 270 289 315 337 408 410 418<br>421 464 475 508 550 584 626 629 648 661 691 709 713 717 740 769 791 807 840<br>847 863 865 866 935 947 957 963 974 978 985 1004 1007 1015 1016

	1017	1035	1060	1064	1068	1114	1115	1143	1173	1189
	1191	1207	1209	1213	1249	1273	1312	1335	1349	1351
	1358	1376	1384	1434	1458	1490	1493	1509	1513	1538
	1542	1555	1627	1644	1646	1664	1669	1676	1696	3047
	3111	3139	3187	3188	3197	3197	3205	3207	3213	3214
	3218	3221	3223	3228	3228	3234				

Table S6. The energies, enthalpies and free energies (in au at 298 K) and corresponding relative values with ZPE correction (in kcal/mol) for **4**+Ph_2CO, obtained with B3PW91-PCM//B3PW91-PCM/6-31G(d)/ECP80MWB method

species	E	ZPE	G	H	S (sol)
**4**	-3582.63508	1.06857	-3582.73897	-3582.55804	380.8
**Ph_2CO**	-576.21078	0.19242	-576.23790	-576.19906	81.7
**4+ Ph_2CO**	-4158.84585 (0.0)	1.26099	-4158.97687 (0.0)	-4158.75710 (0.0)	462.5
**TS5a**	-4158.79361 (32.8)	1.26244	-4158.91061 (41.6)	-4158.70538 (32.5)	431.9
**INT5**	-4158.83811 (4.9)	1.26381	-4158.95463 (14.0)	-4158.74966 (4.7)	431.4
**TS5b**	-4158.79548 (31.6)	1.26242	-4158.91216 (40.6)	-4158.707514 (31.1)	430.7
**5**	-3332.38749	0.94845	-3332.48329	-3332.31732	349.3
**Ph_2CN(*p*-totyl)**	-826.47814	0.31275	-826.51574	-826.45966	118.0
**5+Ph_2CN(*p*-totyl)**	-4158.86563 (-12.4)	1.26120	-4158.99903 (-13.9)	-4158.77698 (-12.5)	467.3

Table S7. The energies, enthalpies and free energies (in au at 298 K) and corresponding relative values with ZPE correction (in kcal/mol) for **4**+Ph_2CO, obtained with B3PW91-D3-PCM/6-31G(d)/ECP80MWB method

species	E	ZPE	G	H	S (sol)
**4**	-3582.99679	1.06857	-3583.10068	-3582.91974	380.8
**Ph_2CO**	-576.26261	0.19242	-576.28973	-576.25089	81.7
**4+ Ph_2CO**	-4159.25939 (0.0)	1.26099	-4159.39041 (0.0)	-4159.17063 (0.0)	462.5
**TS5a**	-4159.25216 (4.5)	1.26244	-4159.36916 (13.3)	-4159.16393 (4.2)	431.9
**INT5**	-4159.29245 (-20.7)	1.26381	-4159.40897 (-11.6)	-4159.20399 (-20.9)	431.4
**TS5b**	-4159.25219 (4.5)	1.26242	-4159.36888 (13.5)	-4159.16423 (4.0)	430.7
**5**	-3332.72195	0.94845	-3332.81775	-3332.65178	349.3
**Ph_2CN(*p*-totyl)**	-826.56746	0.31275	-826.60506	-826.54898	118.0
**5+Ph_2CN(*p*-totyl)**	-4159.28941 (-18.8)	1.26120	-4159.42282 (-20.3)	-4159.20076 (18.9)	467.3

Table S8. The optimized Cartesian Coordinates (in Å) and structures (the hydrogen atoms omitted for clarity) of stationary points for **5**+Me_3SiCl, obtained with B3PW91-PCM/6-31G(d)/ECP80MWB method.

Species	Cartesian coordinates				
	U	6.650204	16.809966	4.699887	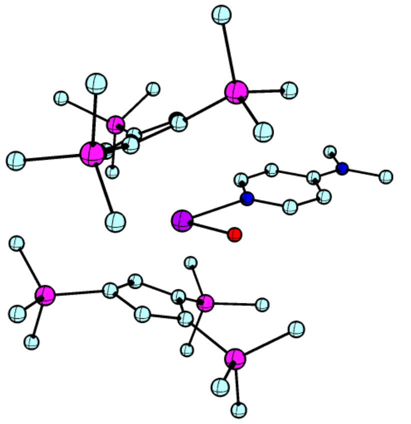 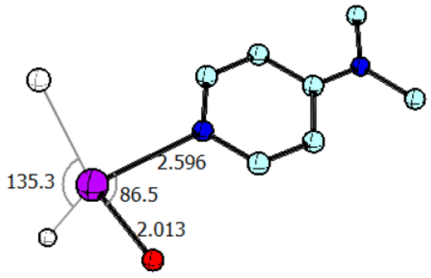
	Si	10.800925	16.720879	4.253365	
	Si	8.828948	13.278501	4.513175	
	Si	6.562856	16.533691	0.280039	
	Si	2.665749	18.688062	5.108848	
	Si	5.155349	18.391011	8.228124	
	Si	7.646715	21.138690	3.849132	
	O	7.078392	15.880492	6.433610	
	N	4.647183	15.171072	4.498137	
	N	1.540715	12.345574	4.443685	
	C	9.157773	16.150298	3.508713	
	C	8.459894	14.883959	3.588647	
	C	7.418171	14.937698	2.621940	
	H	6.731837	14.120744	2.420766	
	C	7.446830	16.168476	1.894257	
	C	8.517112	16.901945	2.469144	
	H	8.843693	17.881298	2.136313	
	C	11.432302	18.171594	3.208722	
	H	11.604582	17.863246	2.170589	
	H	12.387642	18.531090	3.611325	
	H	10.744703	19.024189	3.193040	
	C	10.687084	17.283282	6.057422	
	H	10.059549	18.176194	6.168893	
	H	11.686229	17.536435	6.435166	
	H	10.264962	16.506661	6.703841	
	C	12.127287	15.375650	4.128379	
**5**	H	11.925330	14.514099	4.771890	
	H	13.096692	15.792775	4.430602	
	H	12.225823	15.012978	3.098653	
	C	10.107191	12.271292	3.532956	
	H	9.745847	12.078583	2.515144	
	H	10.275836	11.299825	4.015979	
	H	11.075278	12.775168	3.450838	
	C	7.257580	12.218621	4.534686	
	H	6.437461	12.713067	5.064577	
	H	7.463033	11.270402	5.047921	
	H	6.911388	11.973103	3.523578	
	C	9.389380	13.534241	6.294025	
	H	10.378732	13.997592	6.374737	
	H	9.430827	12.572405	6.821283	
	H	8.660683	14.188570	6.788724	
	C	5.761843	14.936767	-0.349120	
	H	6.513166	14.151296	-0.491555	
	H	5.270953	15.108871	-1.315032	
	H	5.003516	14.546440	0.340369	
	C	5.225079	17.870224	0.408529	
	H	4.417193	17.597947	1.097764	
	H	4.771562	18.048793	-0.574878	
	H	5.647543	18.820621	0.754003	
	C	7.841859	17.121070	-0.984555	
	H	8.329911	18.047253	-0.658201	
	H	7.374353	17.317589	-1.957519	
	H	8.624727	16.368003	-1.131188	

	C	4.543080	18.798681	5.241798	
	C	5.420598	18.787866	6.398241	
	C	6.644307	19.374271	5.975773	
	H	7.500707	19.536162	6.623374	
	C	6.572451	19.796367	4.607822	
	C	5.272705	19.416831	4.179430	
	H	4.868370	19.611116	3.189566	
	C	2.159081	18.341690	3.315261	
	H	2.594709	19.060178	2.611757	
	H	1.068026	18.414527	3.221315	
	H	2.448573	17.334676	2.996417	
	C	1.839753	17.377846	6.194240	
	H	2.199169	16.367950	5.968798	
	H	0.757009	17.395150	6.013804	
	H	1.995057	17.559865	7.262384	
	C	1.948133	20.383044	5.564462	
	H	2.204871	20.673523	6.588845	
	H	0.853948	20.382739	5.477830	
	H	2.338289	21.159002	4.895120	
	C	4.668926	16.607709	8.613921	
	H	3.742297	16.293998	8.122404	
	H	4.533890	16.482011	9.696107	
	H	5.482678	15.955573	8.277153	
	C	3.847927	19.564532	8.950567	
	H	4.109632	20.612618	8.761810	
	H	3.780775	19.427380	10.037703	
	H	2.848474	19.391368	8.536402	
	C	6.779772	18.731630	9.137116	
	H	7.582645	18.089732	8.757594	
	H	6.657254	18.513511	10.205592	
	H	7.098582	19.776761	9.047614	
	C	6.691803	22.768566	4.009019	
	H	5.754149	22.736603	3.441286	
	H	7.283698	23.612662	3.632637	
	H	6.437401	22.974442	5.055379	
	C	9.257477	21.309767	4.824570	
	H	9.884763	22.097878	4.390109	
	H	9.847081	20.387127	4.837706	
	H	9.053750	21.589372	5.865114	
	C	8.009273	20.888600	2.008191	
	H	7.085689	20.728756	1.439015	
	H	8.672918	20.039461	1.815440	
	H	8.493177	21.784480	1.599059	
	C	3.894967	14.959934	3.400130	
	H	4.143459	15.554222	2.525401	
	C	2.864305	14.047261	3.328758	
	H	2.318914	13.950826	2.398049	
	C	2.544669	13.256685	4.462512	
	C	3.345277	13.477131	5.610844	
	H	3.190936	12.916103	6.524280	
	C	4.357501	14.414525	5.579589	
	H	4.993263	14.585038	6.442893	
	C	0.756752	12.150140	3.235969	
	H	0.003599	11.383435	3.418913	
	H	1.383554	11.817383	2.398705	
	H	0.238637	13.071282	2.940369	

	C	1.251964	11.555157	5.628998	
	H	2.110176	10.935386	5.918263	
	H	0.411156	10.893238	5.420393	
	H	0.979670	12.193367	6.478935	
**Me$_3$SiCl**	Si	-0.560091	0.056972	0.321323	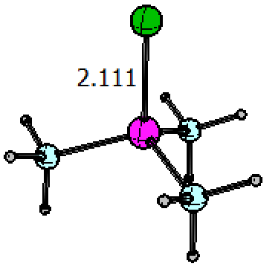
	C	0.591773	1.104670	1.369020	
	C	-1.607788	-1.094891	1.369020	
	C	-1.607788	1.104670	-0.830541	
	H	1.234617	1.733735	0.743728	
	H	1.234616	0.479377	1.998086	
	H	0.011059	1.762993	2.027343	
	H	-2.236853	-1.737735	0.743728	
	H	-0.982496	-1.737735	1.998085	
	H	-2.266111	-0.514177	2.027343	
	H	-2.236853	0.479377	-1.473384	
	H	-0.982496	1.733735	-1.473385	
	H	-2.266111	1.762992	-0.249827	
	Cl	0.658507	-1.161625	-0.897275	
**TS8a**	U	6.773680	16.245771	5.704589	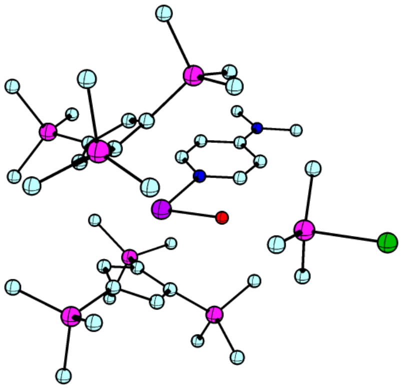 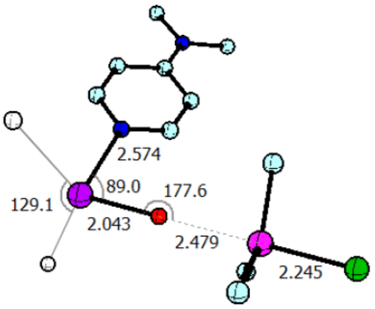
	Si	10.943087	15.953293	4.336005	
	Si	8.810129	12.611994	4.706148	
	Si	6.380867	16.206472	1.064777	
	Si	2.764152	18.308099	4.931591	
	Si	3.897621	18.180624	8.688940	
	Si	8.108270	20.303205	5.543553	
	O	7.424749	15.461579	7.474720	
	N	4.803016	14.589805	5.747101	
	N	1.801056	11.664363	5.990346	
	C	9.098657	15.595220	4.071122	
	C	8.341199	14.362997	4.176970	
	C	7.180002	14.532798	3.365528	
	H	6.440238	13.755076	3.198974	
	C	7.182763	15.800794	2.715600	
	C	8.351016	16.449848	3.198812	
	H	8.682944	17.432462	2.879928	
	C	11.348587	17.620248	3.531750	
	H	11.152169	17.606248	2.453255	
	H	12.416869	17.831240	3.667386	
	H	10.795705	18.459782	3.964856	
	C	11.554489	16.005609	6.120319	
	H	11.107137	16.827255	6.689699	
	H	12.641517	16.158863	6.123172	
	H	11.346095	15.078750	6.661220	
	C	11.968506	14.670657	3.390617	
	H	11.913593	13.669328	3.828333	
	H	13.023585	14.973323	3.393175	
	H	11.643399	14.599515	2.346044	
	C	9.533447	11.716256	3.197129	
	H	8.799787	11.680458	2.382551	
	H	9.795580	10.681999	3.455265	
	H	10.433666	12.203539	2.809528	
	C	7.244370	11.643176	5.154549	
	H	6.687196	12.077598	5.989406	
	H	7.518203	10.618660	5.437020	
	H	6.562185	11.571022	4.298698	
	C	10.053349	12.533161	6.124052	

	H 11.061030 12.817812 5.803577<br>H 10.111187 11.507458 6.509269<br>H 9.762621 13.186353 6.951731<br>C 4.791661 15.215277 0.782464<br>H 4.964727 14.143649 0.939569<br>H 4.463167 15.341721 -0.256757<br>H 3.959018 15.524594 1.422539<br>C 6.084783 18.058574 0.840820<br>H 5.330890 18.447365 1.531154<br>H 5.742222 18.268094 -0.180113<br>H 7.009780 18.625518 0.999036<br>C 7.609649 15.662004 -0.271827<br>H 8.561581 16.197374 -0.175112<br>H 7.213467 15.859509 -1.276126<br>H 7.825531 14.589520 -0.199282<br>C 4.492810 18.321430 5.692173<br>C 4.908076 18.260804 7.087115<br>C 6.251183 18.708614 7.126759<br>H 6.838245 18.811379 8.034153<br>C 6.714431 19.070735 5.819119<br>C 5.599404 18.822254 4.956812<br>H 5.594789 19.022107 3.888995<br>C 2.867231 17.913857 3.084347<br>H 3.346020 18.726563 2.527294<br>H 1.857553 17.791058 2.672819<br>H 3.424440 16.995093 2.879870<br>C 1.561872 17.069591 5.707075<br>H 1.921726 16.038972 5.615645<br>H 0.598923 17.133548 5.183869<br>H 1.370514 17.263967 6.766282<br>C 2.028174 20.052260 5.031174<br>H 1.913377 20.411083 6.057874<br>H 1.040735 20.079399 4.552278<br>H 2.674074 20.763352 4.501902<br>C 3.196021 16.478489 9.131333<br>H 2.561030 16.070390 8.338311<br>H 2.584559 16.561365 10.039228<br>H 3.988712 15.752605 9.334204<br>C 2.446410 19.402123 8.640129<br>H 2.788203 20.409157 8.373947<br>H 2.006197 19.458219 9.644416<br>H 1.646857 19.122559 7.948205<br>C 4.985296 18.811593 10.104538<br>H 5.913088 18.248880 10.236294<br>H 4.427832 18.749062 11.047745<br>H 5.245709 19.864937 9.944965<br>C 7.532147 21.967101 6.241586<br>H 6.619564 22.312103 5.741350<br>H 8.302115 22.737525 6.107813<br>H 7.314904 21.892321 7.313546<br>C 9.659630 19.787495 6.492352<br>H 10.451157 20.539122 6.383269<br>H 10.063822 18.829688 6.148887<br>H 9.445936 19.689996 7.563766<br>C 8.465686 20.548536 3.703592<br>H 7.562410 20.871573 3.172448	

	H 8.836831 19.643745 3.212614<br>H 9.222221 21.331539 3.569032<br>C 3.998671 14.305787 4.701707<br>H 4.177556 14.869923 3.792252<br>C 2.998784 13.358207 4.728933<br>H 2.407725 13.202131 3.835032<br>C 2.765837 12.611612 5.913253<br>C 3.611567 12.918643 7.008676<br>H 3.520074 12.403199 7.956548<br>C 4.587680 13.883097 6.879172<br>H 5.252675 14.111221 7.705397<br>C 0.968696 11.372693 4.834724<br>H 0.268209 10.577839 5.091226<br>H 1.570549 11.032528 3.982523<br>H 0.387879 12.250926 4.525542<br>C 1.603564 10.926144 7.227740<br>H 2.499809 10.355871 7.502114<br>H 0.781172 10.222826 7.096364<br>H 1.347785 11.597331 8.056817<br>Si 8.117824 14.521314 9.661296<br>C 6.709611 15.560593 10.337502<br>C 7.694542 12.860151 8.881911<br>C 9.754221 15.375634 9.312342<br>H 7.091238 16.252696 11.096498<br>H 5.965048 14.923143 10.828259<br>H 6.237577 16.126637 9.535236<br>H 8.537553 12.169338 8.986810<br>H 6.847149 12.416186 9.418762<br>H 7.443354 12.962899 7.826890<br>H 10.563325 14.639844 9.242829<br>H 9.995673 16.044852 10.146164<br>H 9.708897 15.949105 8.387297<br>Cl 8.726391 13.657770 11.641802	
**TS8b**	U 6.970957 16.855643 5.170435<br>Si 11.118590 16.569620 3.839269<br>Si 9.029555 13.143498 4.120953<br>Si 6.518628 16.682183 0.572635<br>Si 2.801491 18.757122 4.375966<br>Si 4.080573 18.604659 8.099802<br>Si 7.995877 21.015902 4.775582<br>O 7.705212 16.177050 6.960165<br>N 5.058743 15.140901 5.284836<br>N 2.139289 12.144511 5.634935<br>C 9.292468 16.164687 3.545898<br>C 8.562186 14.921385 3.669434<br>C 7.381963 15.069196 2.877679<br>H 6.651111 14.279076 2.725308<br>C 7.353518 16.330629 2.218244<br>C 8.518063 17.004609 2.681972<br>H 8.828368 17.991034 2.353081<br>C 11.448165 18.356862 3.307326<br>H 11.223131 18.518728 2.246605<br>H 12.513099 18.579136 3.451327<br>H 10.883307 19.090666 3.890474<br>C 11.727455 16.373980 5.612502<br>H 11.090931 16.914149 6.322318	

H	12.744664	16.777474	5.696688
H	11.758938	15.326257	5.923706
C	12.148469	15.493508	2.664054
H	11.989943	14.420635	2.811020
H	13.218920	15.694921	2.798586
H	11.894280	15.723588	1.622194
C	9.231392	12.224833	2.471489
H	8.313351	12.263966	1.873298
H	9.479369	11.168981	2.639831
H	10.035099	12.665122	1.869244
C	7.610608	12.320474	5.065418
H	7.480542	12.762425	6.059046
H	7.815363	11.250449	5.197756
H	6.657245	12.412367	4.532217
C	10.624491	12.860673	5.091782
H	11.515515	13.210552	4.561590
H	10.736812	11.777423	5.233104
H	10.611153	13.321288	6.082149
C	4.963631	15.627243	0.335147
H	5.175602	14.566128	0.514048
H	4.612927	15.718834	-0.700410
H	4.132501	15.922593	0.983750
C	6.141753	18.514663	0.311992
H	5.368939	18.881736	0.993512
H	5.791824	18.687874	-0.713282
H	7.040191	19.125177	0.460340
C	7.749462	16.158997	-0.770669
H	8.680146	16.734263	-0.699696
H	7.331246	16.317992	-1.772882
H	8.009798	15.098020	-0.677869
C	4.552695	18.790000	5.082536
C	5.020042	18.748819	6.461745
C	6.333649	19.286776	6.460279
H	6.951242	19.410947	7.343605
C	6.728880	19.669499	5.137145
C	5.605207	19.347535	4.312016
H	5.554971	19.545596	3.245382
C	2.832132	18.344315	2.528928
H	3.356646	19.111893	1.949802
H	1.803910	18.299669	2.148234
H	3.306446	17.381842	2.315343
C	1.608276	17.539836	5.197308
H	2.001624	16.517678	5.201493
H	0.667602	17.533572	4.631457
H	1.365008	17.812396	6.228574
C	2.076284	20.504896	4.491829
H	2.025265	20.874234	5.520499
H	1.061793	20.529146	4.073255
H	2.690870	21.209288	3.918374
C	3.287517	16.923298	8.467009
H	2.643049	16.578114	7.653279
H	2.667658	17.011331	9.369043
H	4.031102	16.144022	8.656500
C	2.694369	19.899553	8.161164
H	3.077379	20.897281	7.916588
H	2.286013	19.941254	9.179457

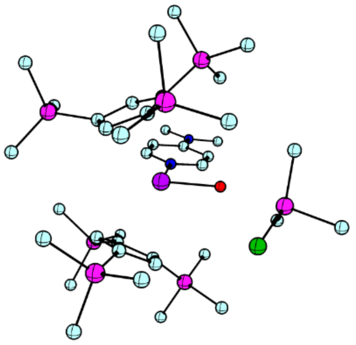

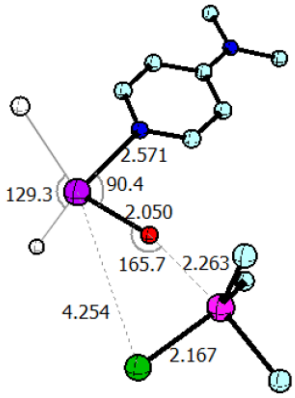

	H 1.862449 19.682965 7.483683<br>C 5.261193 19.064318 9.503821<br>H 6.194754 18.494103 9.494476<br>H 4.771834 18.889094 10.470274<br>H 5.521791 20.128445 9.455390<br>C 7.152648 22.667665 5.166943<br>H 6.266165 22.820380 4.539933<br>H 7.835884 23.509301 4.995764<br>H 6.829294 22.704547 6.213826<br>C 9.508558 20.864681 5.895878<br>H 10.235334 21.652673 5.661783<br>H 10.020772 19.900955 5.810492<br>H 9.222881 20.981835 6.947585<br>C 8.487203 21.056698 2.949082<br>H 7.602616 21.068419 2.301184<br>H 9.110599 20.207013 2.654650<br>H 9.058022 21.970117 2.739824<br>C 4.190464 14.873908 4.288010<br>H 4.294710 15.475487 3.391005<br>C 3.216934 13.900545 4.349731<br>H 2.567453 13.761038 3.494530<br>C 3.082991 13.110085 5.520949<br>C 4.002780 13.392636 6.561050<br>H 3.991266 12.840196 7.492350<br>C 4.946162 14.384608 6.398755<br>H 5.665688 14.597476 7.182650<br>C 1.222635 11.884782 4.537227<br>H 0.553609 11.070525 4.816106<br>H 1.759796 11.585710 3.628331<br>H 0.609311 12.765550 4.307811<br>C 2.036644 11.369919 6.861077<br>H 2.956328 10.804321 7.056528<br>H 1.215563 10.659045 6.766909<br>H 1.831549 12.014094 7.725182<br>Si 8.611759 15.977449 9.024057<br>C 9.576340 14.557403 8.241403<br>C 6.818051 15.658572 9.543270<br>C 9.449627 15.984975 10.763292<br>H 10.561564 14.912553 7.919869<br>H 9.058034 14.144688 7.376496<br>H 9.742784 13.772524 8.990808<br>H 6.109387 16.222766 8.939779<br>H 6.611447 14.589033 9.396618<br>H 6.655737 15.885504 10.602857<br>H 9.072404 16.792880 11.403297<br>H 10.539929 16.097695 10.700987<br>H 9.245808 15.034241 11.279272<br>Cl 9.234624 18.006083 8.583355	
**INT8**	U 0.481417 0.321509 0.177782<br>Si 4.579903 -0.622970 -1.080981<br>Si 2.007603 -3.671695 -1.198123<br>Si 0.092051 0.492540 -4.343335<br>Si -3.906108 1.643942 -0.551630<br>Si -2.417334 1.860866 3.101129<br>Si 0.976646 4.494706 -0.646177<br>O 0.708776 -0.688981 2.151999	

N	-1.494376	-1.505833	0.229320
N	-4.481577	-4.469430	0.365818
C	2.715087	-0.693685	-1.433624
C	1.793217	-1.805441	-1.434707
C	0.654195	-1.381829	-2.185553
H	-0.181627	-2.028811	-2.429365
C	0.828359	-0.062921	-2.696962
C	2.091582	0.354977	-2.177080
H	2.556911	1.312343	-2.382181
C	5.235582	1.100310	-1.492565
H	5.021074	1.386304	-2.528968
H	6.325950	1.112415	-1.367547
H	4.810030	1.855430	-0.825228
C	5.113076	-1.068314	0.673129
H	4.622917	-0.405184	1.392219
H	6.198508	-0.935906	0.769652
H	4.880681	-2.103987	0.936393
C	5.420803	-1.802866	-2.311054
H	5.125135	-2.849168	-2.183802
H	6.510952	-1.750366	-2.192461
H	5.185093	-1.515141	-3.342880
C	2.334796	-4.379676	-2.928565
H	1.500621	-4.161312	-3.606245
H	2.458163	-5.469573	-2.888322
H	3.240994	-3.955398	-3.375661
C	0.408779	-4.453366	-0.559332
H	0.216782	-4.178778	0.482954
H	0.491902	-5.546809	-0.607526
H	-0.469638	-4.162327	-1.145896
C	3.395922	-4.259684	-0.061606
H	4.391012	-3.948791	-0.392151
H	3.382862	-5.357720	-0.056304
H	3.257788	-3.924730	0.970214
C	-1.367742	-0.618575	-4.807512
H	-1.076832	-1.675255	-4.825923
H	-1.721347	-0.358438	-5.813108
H	-2.218624	-0.508811	-4.126972
C	-0.434265	2.303977	-4.497219
H	-1.401210	2.514370	-4.028988
H	-0.530513	2.552187	-5.561959
H	0.304718	2.989377	-4.068782
C	1.467243	0.214808	-5.616517
H	2.327704	0.864110	-5.415746
H	1.114398	0.430085	-6.633100
H	1.823219	-0.821675	-5.592857
C	-2.119898	1.819427	0.047906
C	-1.613234	2.012672	1.394847
C	-0.404780	2.750653	1.269227
H	0.216380	3.065141	2.099651
C	-0.123010	3.056067	-0.099455
C	-1.186720	2.450860	-0.827530
H	-1.310685	2.527471	-1.901564
C	-4.014476	1.073146	-2.355825
H	-3.279459	1.563750	-3.002049
H	-5.008073	1.331071	-2.744490
H	-3.897411	-0.009306	-2.467204

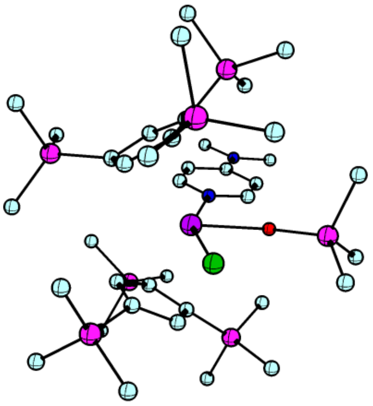

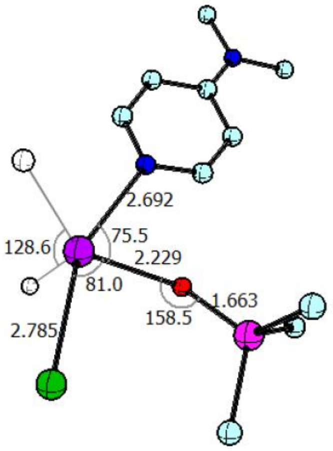

	C -5.039005 0.515024 0.456375<br>H -4.639041 -0.499287 0.553499<br>H -6.004721 0.444294 -0.061283<br>H -5.237300 0.903312 1.459831<br>C -4.631133 3.396203 -0.528035<br>H -4.576832 3.854304 0.464777<br>H -5.683733 3.385756 -0.839082<br>H -4.083084 4.046288 -1.220483<br>C -3.095762 0.156103 3.551367<br>H -3.812386 -0.228474 2.821017<br>H -3.605941 0.217184 4.521614<br>H -2.289476 -0.575575 3.646753<br>C -3.829992 3.119898 3.244439<br>H -3.486785 4.125830 2.975341<br>H -4.186532 3.156248 4.282107<br>H -4.689682 2.879086 2.610491<br>C -1.135523 2.345266 4.403232<br>H -0.233417 1.728818 4.338805<br>H -1.559193 2.214870 5.406989<br>H -0.833474 3.394693 4.308761<br>C -0.175412 5.705715 -1.544636<br>H -0.597658 5.260504 -2.453741<br>H 0.368024 6.612062 -1.840712<br>H -1.011467 6.008675 -0.903374<br>C 1.668332 5.335245 0.896205<br>H 2.261461 6.214403 0.614746<br>H 2.318056 4.652381 1.454004<br>H 0.868245 5.672658 1.565648<br>C 2.404574 4.095037 -1.814745<br>H 2.066998 3.637688 -2.751306<br>H 3.123318 3.422227 -1.338470<br>H 2.928584 5.024082 -2.074371<br>C -2.301736 -1.758963 -0.817680<br>H -2.138284 -1.155332 -1.703310<br>C -3.293268 -2.719206 -0.830452<br>H -3.884131 -2.845323 -1.729673<br>C -3.521227 -3.510531 0.323498<br>C -2.679737 -3.232476 1.426640<br>H -2.769633 -3.778134 2.358110<br>C -1.709409 -2.254315 1.327610<br>H -1.044911 -2.041811 2.157120<br>C -5.312359 -4.722137 -0.798439<br>H -6.005632 -5.533095 -0.573503<br>H -4.709492 -5.022844 -1.664990<br>H -5.901882 -3.837941 -1.073981<br>C -4.679895 -5.244016 1.578602<br>H -3.781548 -5.817174 1.841609<br>H -5.497328 -5.948676 1.423353<br>H -4.943133 -4.600204 2.427553<br>Si 1.409444 -1.123450 3.596031<br>C 2.474228 -2.667865 3.372919<br>C 0.083925 -1.574126 4.875881<br>C 2.490722 0.227567 4.343490<br>H 3.325168 -2.468300 2.713441<br>H 1.897905 -3.497195 2.945446<br>H 2.874388 -3.003584 4.338252	

	H	-0.583863	-2.372609	4.528822	
	H	0.573910	-1.938126	5.788713	
	H	-0.535880	-0.716823	5.159541	
	H	1.937210	1.155460	4.523603	
	H	3.325040	0.475632	3.680717	
	H	2.897570	-0.113228	5.305004	
	Cl	2.728323	1.679141	1.106058	
**8**	U	5.609093	10.487666	13.491877	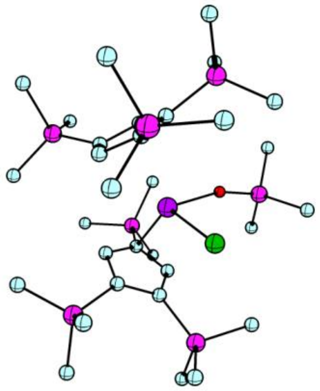 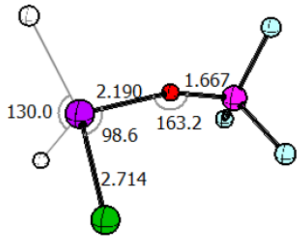
	Cl	3.122307	10.768853	14.541157	
	Si	4.840320	6.368426	12.811983	
	Si	4.257013	7.683166	16.519889	
	Si	9.569120	8.742471	14.832776	
	Si	3.912276	14.476703	13.553021	
	Si	6.384806	13.520713	16.522913	
	Si	8.750281	12.275192	11.348876	
	Si	4.343681	10.222126	9.900701	
	O	5.045343	10.111772	11.409286	
	C	5.757904	7.660687	13.851529	
	C	5.532771	8.156352	15.194937	
	C	6.715924	8.855750	15.573872	
	H	6.875738	9.306001	16.550159	
	C	7.701349	8.800883	14.538082	
	C	7.070030	8.077391	13.479323	
	H	7.548062	7.822409	12.538725	
	C	5.926461	5.975300	11.313715	
	H	6.156499	6.863378	10.716337	
	H	5.409589	5.262591	10.658944	
	H	6.873155	5.513475	11.618615	
	C	4.680340	4.754378	13.791127	
	H	5.657735	4.418529	14.157272	
	H	4.282293	3.968711	13.136005	
	H	4.009808	4.834152	14.652498	
	C	3.135023	6.913207	12.218055	
	H	2.466220	7.168840	13.045164	
	H	2.662375	6.111024	11.637190	
	H	3.216177	7.794139	11.575153	
	C	2.521429	7.306179	15.884116	
	H	2.112792	8.154152	15.325513	
	H	1.865717	7.123547	16.745348	
	H	2.478094	6.416720	15.248205	
	C	4.945292	6.154081	17.406992	
	H	5.085951	5.305420	16.729328	
	H	4.262048	5.836871	18.205246	
	H	5.915879	6.375440	17.867151	
	C	4.129126	9.061406	17.803513	
	H	5.098765	9.304952	18.253844	
	H	3.464638	8.743490	18.616791	
	H	3.709702	9.971363	17.363798	
	C	10.509879	8.496346	13.213484	
	H	10.133221	7.629142	12.658480	
	H	11.567049	8.305268	13.436667	
	H	10.466599	9.365867	12.551271	
	C	10.293169	10.202123	15.787725	
	H	10.284015	11.143315	15.229166	
	H	11.339845	9.978497	16.031023	
	H	9.769315	10.367615	16.735262	

	C	9.835079	7.196718	15.894894	
	H	9.329494	7.286536	16.863544	
	H	10.902833	7.032406	16.087023	
	H	9.440441	6.303151	15.397432	
	C	5.457401	13.374801	13.630677	
	C	6.364177	13.000144	14.699853	
	C	7.520900	12.445406	14.075381	
	H	8.400595	12.105820	14.609918	
	C	7.391828	12.461963	12.649855	
	C	6.106174	13.034771	12.412106	
	H	5.691511	13.212090	11.426230	
	C	2.799624	13.888524	12.144938	
	H	3.314481	13.868984	11.177948	
	H	1.940245	14.563199	12.043382	
	H	2.414802	12.884256	12.351568	
	C	4.526053	16.221165	13.133766	
	H	5.192231	16.619119	13.907408	
	H	3.681808	16.914347	13.028799	
	H	5.080674	16.223424	12.187811	
	C	2.857285	14.553798	15.114658	
	H	2.480797	13.562692	15.387046	
	H	1.989979	15.195342	14.910557	
	H	3.379263	14.978083	15.977734	
	C	6.436447	15.412110	16.597393	
	H	7.280632	15.800412	16.015328	
	H	6.563982	15.744544	17.635452	
	H	5.524281	15.877719	16.210754	
	C	4.953925	12.867352	17.565149	
	H	3.980950	12.978064	17.080459	
	H	4.919159	13.401632	18.523254	
	H	5.091743	11.804182	17.787208	
	C	7.980263	12.887242	17.316799	
	H	8.028028	11.792684	17.321022	
	H	8.020068	13.219417	18.361836	
	H	8.879779	13.263742	16.817173	
	C	10.427339	12.473651	12.197437	
	H	10.497102	13.446021	12.699146	
	H	11.235322	12.421952	11.457150	
	H	10.618934	11.697430	12.945630	
	C	8.539265	13.686238	10.108795	
	H	7.575923	13.636156	9.588943	
	H	9.328191	13.651174	9.347096	
	H	8.598356	14.659916	10.608969	
	C	8.684174	10.631531	10.416002	
	H	8.753771	9.768482	11.086781	
	H	9.512596	10.564508	9.699627	
	H	7.747972	10.534888	9.856636	
	C	4.923964	11.760858	8.965663	
	H	4.611174	12.689589	9.455816	
	H	4.481938	11.762086	7.960776	
	H	6.012364	11.791578	8.843086	
	C	2.465267	10.319958	10.049413	
	H	2.041601	9.460184	10.579602	
	H	2.009879	10.353743	9.051156	
	H	2.156055	11.223748	10.585928	
	C	4.811280	8.724685	8.849626	

	H	5.899181	8.610197	8.771842	
	H	4.418160	8.843131	7.831631	
	H	4.404404	7.791811	9.254360	
**DMAP**	N	10.677584	4.119574	6.630946	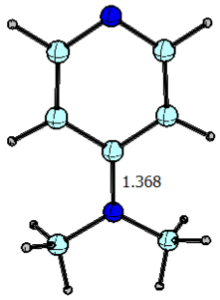
	N	13.548695	4.052009	9.729144	
	C	10.378385	3.739019	7.879752	
	H	9.343339	3.443050	8.052362	
	C	11.272850	3.698966	8.939686	
	H	10.923026	3.377133	9.914015	
	C	12.617899	4.078686	8.726893	
	C	12.939817	4.486243	7.412016	
	H	13.942271	4.803080	7.147467	
	C	11.952239	4.482670	6.437369	
	H	12.209153	4.797431	5.425637	
	C	14.902288	4.500300	9.471144	
	H	15.379904	3.903564	8.682759	
	H	15.496216	4.389513	10.379695	
	H	14.936262	5.556834	9.169070	
	C	13.154623	3.677386	11.072137	
	H	12.407789	4.367343	11.490474	
	H	14.031487	3.694414	11.721217	
	H	12.736209	2.662563	11.099869	
**INT8a**	U	7.115675	17.303146	4.805345	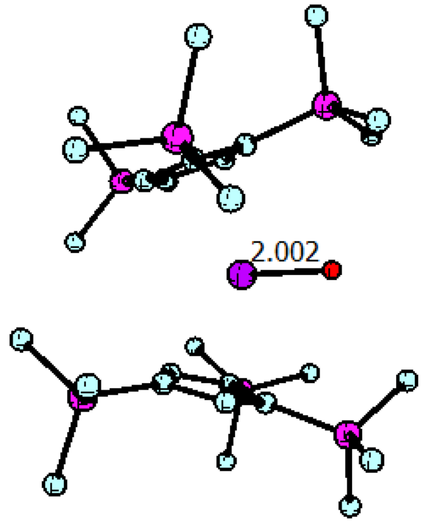
	Si	11.169998	16.899828	3.952114	
	Si	8.925095	13.687790	4.702679	
	Si	6.561350	16.757752	0.393327	
	Si	3.011120	18.666423	4.813469	
	Si	5.105639	18.498551	8.235102	
	Si	7.987446	21.528016	4.223230	
	O	7.356224	16.240995	6.485173	
	C	9.416714	16.436865	3.406920	
	C	8.638294	15.239207	3.652727	
	C	7.510599	15.312950	2.786242	
	H	6.748074	14.541997	2.707363	
	C	7.563874	16.482853	1.960458	
	C	8.738417	17.164311	2.370513	
	H	9.106061	18.085205	1.928124	
	C	11.758998	18.332277	2.861788	
	H	11.777317	18.046305	1.803551	
	H	12.780090	18.616330	3.145436	
	H	11.135560	19.229045	2.955431	
	C	11.288261	17.447714	5.762298	
	H	10.793050	18.411932	5.932368	
	H	12.341230	17.576583	6.044115	
	H	10.845270	16.719516	6.449285	
	C	12.355270	15.455821	3.657403	
	H	12.140334	14.593744	4.296418	
	H	13.386439	15.770873	3.861617	
	H	12.307855	15.120122	2.614858	
	C	9.852577	12.420502	3.636458	
	H	9.287174	12.188845	2.725522	
	H	9.993489	11.482667	4.189104	
	H	10.841158	12.778987	3.329201	
	C	7.241087	12.948342	5.145749	
	H	6.696944	13.642632	5.795428	
	H	7.378442	12.009535	5.696994	

	H	6.623289	12.721898	4.268673		
	C	9.853788	13.988183	6.315098		
	H	10.881606	14.337837	6.175791		
	H	9.899219	13.052332	6.886941		
	H	9.299979	14.727570	6.904318		
	C	5.038352	15.635614	0.411509		
	H	5.322549	14.579284	0.487344		
	H	4.469684	15.755161	-0.518842		
	H	4.356739	15.859377	1.240400		
	C	6.036731	18.561487	0.183346		
	H	5.326466	18.872615	0.957227		
	H	5.551323	18.707143	-0.789641		
	H	6.898557	19.237476	0.226033		
	C	7.646170	16.274728	-1.080808		
	H	8.545910	16.899271	-1.133058		
	H	7.102457	16.389808	-2.026904		
	H	7.972425	15.230891	-1.004158		
	C	4.823026	19.028000	5.212603		
	C	5.550341	19.031524	6.469605		
	C	6.765252	19.723275	6.223427		
	H	7.523521	19.913921	6.977241		
	C	6.832030	20.189817	4.867897		
	C	5.621682	19.748600	4.269701		
	H	5.330835	19.949560	3.242572		
	C	2.831382	18.386588	2.948769		
	H	3.168252	19.252057	2.366786		
	H	1.774752	18.223336	2.702485		
	H	3.385180	17.508868	2.595565		
	C	2.297006	17.150614	5.689716		
	H	2.889265	16.244158	5.516314		
	H	1.285645	16.959644	5.308833		
	H	2.216037	17.293322	6.771886		
	C	1.976732	20.188440	5.256081		
	H	2.046432	20.437107	6.320230		
	H	0.918861	20.023257	5.015745		
	H	2.316925	21.063311	4.689312		
	C	4.765531	16.657689	8.470589		
	H	3.900738	16.295304	7.905735		
	H	4.575425	16.454733	9.532664		
	H	5.648686	16.092341	8.153337		
	C	3.607772	19.502723	8.824834		
	H	3.771475	20.578527	8.689954		
	H	3.430586	19.322279	9.892849		
	H	2.688389	19.235041	8.291981		
	C	6.573456	18.947150	9.342273		
	H	7.475994	18.401405	9.043558		
	H	6.349294	18.669202	10.379732		
	H	6.796251	20.020541	9.329379		
	C	7.204651	23.207557	4.607290		
	H	6.232216	23.312539	4.112053		
	H	7.847156	24.028824	4.265724		
	H	7.043555	23.331356	5.684552		
	C	9.661079	21.431819	5.098519		
	H	10.301150	22.265105	4.783207		
	H	10.199123	20.504569	4.871714		
	H	9.552516	21.498638	6.187674		

	C	8.229273	21.393415	2.353354	
	H	7.271470	21.422301	1.820782	
	H	8.742646	20.468310	2.068415	
	H	8.833310	22.233056	1.987837	
**TS8c**	U	7.264436	16.594352	5.960222	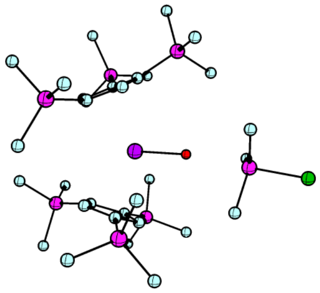 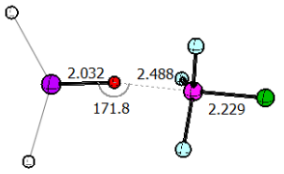
	Si	10.449644	13.801921	5.406709	
	Si	6.792075	12.555945	4.487504	
	Si	8.060105	17.396127	1.644688	
	Si	2.901499	17.127699	4.978854	
	Si	4.277861	16.714373	8.711783	
	Si	7.226598	20.833001	5.818529	
	O	8.595797	16.761050	7.486870	
	C	8.964421	14.695046	4.631749	
	C	7.618776	14.251251	4.303552	
	C	7.075804	15.216017	3.401538	
	H	6.096879	15.140584	2.936421	
	C	8.005095	16.267123	3.159040	
	C	9.154132	15.925558	3.947962	
	H	10.079495	16.493663	3.956147	
	C	11.985958	14.852870	5.070402	
	H	12.161485	14.970427	3.994494	
	H	12.865862	14.353658	5.495213	
	H	11.931481	15.849588	5.519131	
	C	10.295960	13.501656	7.262024	
	H	10.102640	14.444374	7.782377	
	H	11.230231	13.079436	7.653075	
	H	9.489453	12.805161	7.514017	
	C	10.745943	12.154756	4.517650	
	H	9.971320	11.406399	4.712090	
	H	11.700889	11.730087	4.853409	
	H	10.811073	12.298956	3.432732	
	C	7.299436	11.474728	3.020589	
	H	6.984038	11.935038	2.076703	
	H	6.822947	10.488605	3.088174	
	H	8.382146	11.323130	2.969463	
	C	4.913282	12.761786	4.418704	
	H	4.527194	13.243112	5.324334	
	H	4.438776	11.774824	4.352563	
	H	4.578057	13.343348	3.552417	
	C	7.178886	11.664641	6.109708	
	H	8.233516	11.393887	6.214003	
	H	6.599313	10.733214	6.146114	
	H	6.895422	12.254977	6.989230	
	C	7.823836	16.281797	0.134394	
	H	8.584199	15.492980	0.102575	
	H	7.897626	16.860395	-0.794804	
	H	6.840997	15.795700	0.146870	
	C	6.729082	18.735607	1.597738	
	H	5.721866	18.316000	1.697544	
	H	6.772183	19.265478	0.637923	
	H	6.872021	19.478142	2.389636	
	C	9.764142	18.207866	1.559048	
	H	9.977730	18.833831	2.433193	
	H	9.830338	18.850072	0.672303	
	H	10.559429	17.457326	1.483567	
	C	4.511680	17.667895	5.811374	

	C	4.920828	17.665010	7.211853	
	C	5.946064	18.639649	7.344925	
	H	6.440503	18.883608	8.277450	
	C	6.250465	19.233455	6.084489	
	C	5.343285	18.615012	5.159909	
	H	5.268309	18.878441	4.110171	
	C	3.138734	17.123838	3.101183	
	H	3.388840	18.118678	2.715605	
	H	2.207727	16.811229	2.612219	
	H	3.926094	16.430147	2.784140	
	C	2.236491	15.427328	5.468308	
	H	2.859489	14.613690	5.084580	
	H	1.238459	15.305278	5.028004	
	H	2.130996	15.293826	6.548960	
	C	1.590263	18.425868	5.403024	
	H	1.400691	18.476962	6.480914	
	H	0.639689	18.195505	4.905545	
	H	1.908955	19.422500	5.076010	
	C	4.619892	14.866341	8.451750	
	H	4.100123	14.456968	7.578512	
	H	4.307493	14.277724	9.323224	
	H	5.697717	14.685678	8.330087	
	C	2.442072	17.017653	9.043094	
	H	2.260265	18.085854	9.211682	
	H	2.136234	16.482943	9.951229	
	H	1.785601	16.692463	8.230408	
	C	5.236812	17.282714	10.234231	
	H	6.316066	17.132857	10.121769	
	H	4.913034	16.712655	11.113727	
	H	5.063152	18.343944	10.447637	
	C	7.235695	21.804684	7.437107	
	H	6.214850	22.004181	7.783022	
	H	7.735030	22.770546	7.290650	
	H	7.766032	21.278150	8.237738	
	C	9.011081	20.538187	5.260561	
	H	9.536669	21.496402	5.162452	
	H	9.059299	20.041870	4.283815	
	H	9.571150	19.931215	5.981893	
	C	6.327705	21.857205	4.507201	
	H	5.297835	22.069675	4.817257	
	H	6.284828	21.356599	3.533408	
	H	6.837665	22.817385	4.360291	
	Si	10.038943	17.251080	9.453356	
	C	9.146671	18.908639	9.471962	
	C	9.180730	15.732347	10.170691	
	C	11.435376	17.108078	8.200948	
	H	9.868515	19.714535	9.643503	
	H	8.410312	18.946645	10.283229	
	H	8.637498	19.074434	8.521462	
	H	9.923856	14.961821	10.404809	
	H	8.698740	16.010408	11.115306	
	H	8.433899	15.315525	9.494052	
	H	12.039797	16.213562	8.387091	
	H	12.096413	17.976602	8.299253	
	H	11.036830	17.065127	7.186288	
	Cl	11.325270	17.607226	11.238296	

**TS8d**	U	7.471797	16.456629	4.625217	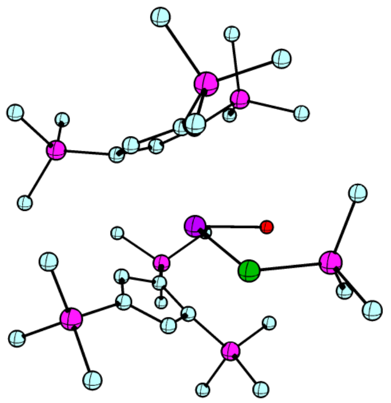 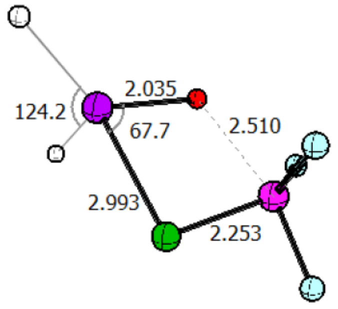
	Si	11.547803	15.859154	4.429558	
	Si	9.390976	12.601950	3.678055	
	Si	7.452407	16.756063	0.298283	
	Si	4.353476	19.181322	5.931076	
	Si	7.492320	18.462367	8.343999	
	Si	9.067908	20.705587	3.359372	
	O	6.925662	15.152987	6.089566	
	C	9.937244	15.609087	3.465670	
	C	9.099712	14.428999	3.307943	
	C	8.111319	14.767446	2.349921	
	H	7.342486	14.087498	1.998266	
	C	8.308762	16.091495	1.839950	
	C	9.443852	16.582161	2.537961	
	H	9.878763	17.564045	2.400088	
	C	12.191687	17.602434	4.078401	
	H	12.476639	17.725954	3.027102	
	H	13.083376	17.796730	4.687163	
	H	11.451514	18.372491	4.321399	
	C	11.314955	15.688404	6.299627	
	H	10.720561	16.513709	6.708082	
	H	12.290387	15.716373	6.802199	
	H	10.822221	14.751954	6.580645	
	C	12.900316	14.648719	3.895211	
	H	12.715642	13.631356	4.254691	
	H	13.861741	14.973616	4.313358	
	H	13.004752	14.611099	2.805047	
	C	10.744155	11.940329	2.524839	
	H	10.481987	12.136123	1.478125	
	H	10.835487	10.852888	2.646322	
	H	11.728399	12.379870	2.709110	
	C	7.831310	11.644539	3.172683	
	H	6.895311	12.127725	3.467811	
	H	7.842145	10.639781	3.612749	
	H	7.807947	11.521936	2.082973	
	C	9.886234	12.220009	5.462749	
	H	10.915154	12.537083	5.668119	
	H	9.839465	11.138270	5.640740	
	H	9.231150	12.710588	6.189280	
	C	6.363418	15.382382	-0.409028	
	H	6.952427	14.492964	-0.661176	
	H	5.868818	15.724845	-1.326346	
	H	5.579041	15.078014	0.294035	
	C	6.358834	18.269220	0.617032	
	H	5.507772	18.024014	1.264250	
	H	5.951058	18.643669	-0.330527	
	H	6.913917	19.089093	1.085590	
	C	8.778008	17.212008	-0.973115	
	H	9.452350	17.992461	-0.602521	
	H	8.321828	17.578861	-1.901020	
	H	9.391662	16.338237	-1.222130	
	C	6.184762	18.823751	5.603498	
	C	7.332046	18.621287	6.462280	
	C	8.481965	18.957104	5.678333	
	H	9.497285	18.963324	6.064759	
	C	8.106121	19.443653	4.386923	

	C	6.696653	19.304198	4.350576	
	H	6.070407	19.613412	3.518027	
	C	3.299629	18.559221	4.486193	
	H	3.673008	18.916124	3.518741	
	H	2.272312	18.930155	4.593416	
	H	3.255016	17.465397	4.445135	
	C	3.621498	18.506475	7.534214	
	H	3.701388	17.419941	7.614270	
	H	2.554419	18.763985	7.557155	
	H	4.080471	18.948016	8.424290	
	C	4.185792	21.069976	5.988751	
	H	4.801340	21.503535	6.785672	
	H	3.144163	21.362553	6.172837	
	H	4.500388	21.526734	5.042971	
	C	6.608002	16.991790	9.125354	
	H	5.521038	17.114414	9.144243	
	H	6.945955	16.869239	10.162422	
	H	6.840031	16.076000	8.570898	
	C	6.887958	20.083460	9.120997	
	H	7.482213	20.930320	8.757069	
	H	6.993001	20.045998	10.212877	
	H	5.837768	20.297088	8.895803	
	C	9.325054	18.304312	8.794587	
	H	9.757533	17.354003	8.463251	
	H	9.421182	18.337252	9.887447	
	H	9.935552	19.119788	8.390434	
	C	7.812477	21.995193	2.770265	
	H	7.091946	21.574096	2.058729	
	H	8.327293	22.820924	2.263290	
	H	7.246697	22.414922	3.609704	
	C	10.305748	21.544774	4.519245	
	H	10.818163	22.364308	4.000186	
	H	11.076039	20.850689	4.875449	
	H	9.801563	21.964823	5.397113	
	C	10.014652	20.129081	1.824952	
	H	9.379614	19.582814	1.120048	
	H	10.880684	19.504147	2.065468	
	H	10.390885	21.018515	1.302761	
	Si	4.863425	13.745368	5.834177	
	C	4.091050	14.741896	7.213508	
	C	6.166935	12.496727	6.349104	
	C	3.452756	12.685166	5.119228	
	H	3.254077	15.341252	6.841363	
	H	4.817515	15.383568	7.710045	
	H	3.686847	14.030057	7.946378	
	H	6.957742	12.381015	5.609316	
	H	6.638404	12.842590	7.271967	
	H	5.692567	11.519607	6.507421	
	H	3.783746	12.090140	4.259203	
	H	2.590500	13.282243	4.801822	
	H	3.110790	11.981809	5.891127	
	Cl	4.930780	15.015661	3.974302	

Table S9. Frequencies of the stationary points optimized for **5**+Me_3SiCl, obtained with B3PW91-PCM/6-31G(d)/ECP80MWB method.

Species	Frequencies (cm^{-1})
**5**	16 21 24 31 34 36 41 45 52 53 54 58 61 63 66 67 73 80<br>81 83 87 95 99 101 109 110 119 124 126 127 132 134 138 140 141 145 146<br>148 151 153 154 156 159 164 166 167 169 170 174 176 176 179 179 183 187 187<br>195 198 199 202 204 207 209 211 212 217 219 219 223 224 226 228 234 237 250<br>264 264 265 272 275 283 286 293 295 299 352 361 367 376 380 386 406 426 433<br>436 488 500 508 552 564 623 623 623 624 636 638 644 648 656 661 675 680 683<br>685 686 687 688 689 689 690 690 691 692 694 695 696 699 707 710 729 751 773<br>775 777 778 778 779 779 780 781 781 783 790 795 834 841 845 850 862 863 864<br>866 867 868 869 871 871 872 873 873 874 876 877 879 885 888 894 899 949 952<br>980 989 1006 1008 1016 1025 1090 1100 1121 1131 1148<br>1148 1151 1182 1190 1215 1247 1250 1267 1289 1289<br>1289 1296 1301 1302 1303 1304 1305 1306 1306 1308<br>1309 1310 1311 1312 1312 1315 1317 1319 1320 1321<br>1323 1372 1386 1393 1401 1434 1465 1473 1474 1474<br>1475 1476 1476 1477 1478 1478 1478 1480 1480 1481<br>1481 1482 1483 1483 1485 1485 1486 1487 1489 1489<br>1490 1491 1491 1492 1493 1493 1494 1496 1497 1498<br>1499 1500 1501 1503 1505 1506 1508 1515 1518 1533<br>1547 1589 1594 1687 3039 3042 3047 3047 3047 3048<br>3048 3049 3050 3050 3051 3051 3052 3052 3052 3054<br>3054 3054 3056 3056 3103 3107 3121 3122 3125 3126<br>3126 3127 3128 3128 3129 3129 3129 3130 3131 3131<br>3131 3132 3132 3133 3136 3136 3138 3139 3139 3141<br>3141 3146 3146 3146 3147 3148 3148 3150 3152 3152<br>3155 3157 3179 3189 3216 3218 3227 3236 3239 3241<br>3258 3260
**Me_3SiCl**	161 167 167 173 173 217 228 228 468 629 709 709 714 787 787 884 889 889<br>1318 1318 1323 1470 1475 1475 1481 1481 1490 3056<br>3056 3059 3139 3139 3141 3152 3153 3153
**TS8a**	-99 9 18 20 22 25 36 38 45 48 50 52 54 57 61 64 66 67<br>70 75 78 78 84 88 89 91 94 98 101 106 108 109 117 119 121 124 125<br>126 131 136 136 137 140 142 144 146 147 149 153 157 160 162 163 164 166 167<br>172 175 178 180 183 184 185 186 188 192 194 194 197 197 205 208 208 210 214<br>214 219 221 224 226 233 234 236 238 240 251 253 261 263 267 269 270 272 273<br>281 287 291 295 302 318 352 357 363 370 375 376 410 423 426 429 489 505 507<br>548 570 591 619 621 623 625 638 639 646 648 651 658 673 680 681 683 685 686<br>686 687 688 690 692 693 694 695 696 698 698 699 703 703 714 736 737 751 773<br>774 776 777 778 778 780 781 783 783 784 786 786 806 808 832 841 843 856 860<br>861 864 866 867 868 869 870 871 873 874 874 876 876 878 879 881 884 886 887<br>890 892 895 942 949 974 980 1000 1002 1009 1032 1091 1103<br>1116 1120 1146 1148 1157 1179 1195 1216 1241 1252<br>1272 1277 1286 1288 1289 1294 1295 1300 1300 1305<br>1306 1307 1308 1309 1309 1310 1311 1312 1313 1314<br>1314 1318 1319 1320 1322 1325 1325 1372 1388 1391<br>1412 1440 1465 1469 1471 1471 1472 1473 1474 1474<br>1475 1475 1476 1477 1477 1478 1479 1479 1479 1480<br>1481 1482 1483 1484 1485 1487 1488 1488 1488 1489<br>1490 1491 1492 1493 1493 1493 1494 1495 1496 1497<br>1499 1500 1501 1502 1504 1505 1506 1507 1510 1511<br>1516 1532 1547 1589 1600 1691 3044 3048 3049 3050<br>3051 3052 3053 3053 3053 3054 3056 3056 3057 3057<br>3057 3059 3060 3060 3061 3063 3063 3066 3068 3106<br>3110 3124 3127 3127 3127 3128 3129 3129 3130 3130

	3132 3132 3133 3133 3134 3136 3136 3136 3137 3138<br>3139 3140 3140 3141 3145 3150 3151 3151 3155 3156<br>3156 3159 3160 3161 3162 3163 3166 3166 3169 3172<br>3182 3191 3200 3209 3210 3221 3225 3233 3237 3238<br>3242 3260 3262
**TS8b**	-98 14 21 23 28 33 38 40 46 48 51 53 55 57 58 62 64 66<br>69 74 77 79 84 84 86 90 93 100 103 105 107 112 118 118 121 124 127<br>128 133 135 137 138 138 143 143 147 149 151 152 155 156 159 162 162 166 169<br>170 173 174 176 177 181 181 185 186 187 193 195 195 202 204 208 213 214 219<br>220 221 225 229 231 234 236 239 242 248 259 261 266 267 269 272 275 278 281<br>287 289 299 303 312 353 358 366 369 370 375 406 410 428 429 433 490 505 510<br>553 570 601 620 623 623 625 637 639 648 648 651 654 656 673 676 681 683 683<br>684 687 687 689 691 691 692 693 694 695 696 697 698 700 702 704 734 751 765<br>772 775 776 779 779 779 780 783 784 785 787 787 789 812 832 844 846 853 861<br>861 863 864 866 867 868 870 871 871 873 874 874 874 876 878 879 880 882 886<br>889 894 898 945 950 981 982 1000 1004 1020 1032 1092 1106<br>1120 1122 1147 1149 1160 1178 1196 1217 1236 1252<br>1278 1280 1288 1289 1290 1292 1295 1301 1301 1304<br>1305 1306 1307 1308 1309 1309 1311 1313 1313 1314<br>1317 1317 1319 1320 1323 1325 1328 1375 1388 1398<br>1410 1441 1466 1471 1472 1473 1474 1474 1474 1476<br>1476 1476 1477 1478 1478 1479 1480 1480 1481 1482<br>1483 1483 1484 1485 1487 1487 1487 1488 1489 1489<br>1490 1490 1491 1492 1492 1492 1493 1495 1495 1496<br>1497 1499 1499 1502 1503 1503 1506 1507 1508 1510<br>1515 1533 1548 1592 1599 1691 3022 3043 3047 3048<br>3048 3048 3050 3051 3052 3052 3053 3054 3055 3055<br>3056 3056 3057 3057 3058 3058 3060 3060 3060 3099<br>3105 3108 3120 3125 3127 3127 3128 3128 3128 3128<br>3129 3130 3130 3130 3130 3133 3133 3134 3134 3135<br>3136 3136 3137 3137 3139 3143 3146 3147 3149 3149<br>3152 3153 3157 3157 3158 3159 3160 3161 3166 3170<br>3180 3183 3191 3204 3211 3212 3229 3240 3241 3244<br>3245 3260 3261
**INT8**	16 24 26 34 37 42 43 48 52 57 58 60 63 64 66 68 72 73<br>78 82 84 87 91 92 98 101 108 110 112 115 116 118 121 124 125 126 131<br>135 138 141 143 146 147 149 151 152 153 154 156 161 163 166 168 171 173 174<br>175 177 179 181 182 186 189 190 195 196 198 201 203 204 206 208 209 211 214<br>217 218 220 222 223 225 226 234 236 238 241 250 264 266 268 270 275 285 285<br>291 292 298 305 308 353 355 364 367 370 374 379 400 429 429 438 489 505 507<br>554 561 620 621 621 623 623 637 638 642 651 653 655 675 678 682 684 685 687<br>687 688 688 690 690 690 691 692 693 693 693 694 697 699 699 704 751 772 774<br>776 776 779 779 779 780 781 782 783 783 784 785 792 831 840 850 857 860 863<br>865 866 867 868 869 869 870 872 872 873 874 875 876 877 879 879 882 884 887<br>891 895 920 945 947 982 991 999 1000 1020 1023 1090 1103 1126<br>1134 1147 1148 1154 1192 1194 1215 1233 1245 1275<br>1285 1290 1293 1295 1299 1302 1302 1304 1304 1305<br>1305 1306 1307 1308 1308 1310 1310 1311 1312 1313<br>1314 1317 1319 1320 1322 1323 1373 1391 1399 1403<br>1436 1464 1471 1473 1474 1474 1475 1475 1476 1477<br>1477 1478 1478 1479 1479 1481 1481 1482 1482 1482<br>1483 1484 1484 1485 1486 1487 1487 1488 1489 1490<br>1490 1490 1491 1492 1492 1494 1495 1496 1497 1498<br>1500 1500 1502 1502 1504 1505 1506 1508 1508 1514<br>1533 1549 1591 1600 1689 3040 3046 3047 3047 3047<br>3048 3050 3050 3050 3052 3053 3053 3054 3054 3056

	3056 3057 3057 3059 3059 3060 3061 3064 3100 3102<br>3118 3125 3126 3126 3127 3127 3128 3129 3129 3131<br>3132 3132 3132 3132 3133 3134 3135 3135 3136 3137<br>3138 3141 3143 3143 3146 3148 3148 3149 3150 3150<br>3151 3151 3153 3155 3157 3160 3163 3163 3166 3172<br>3175 3177 3180 3187 3239 3244 3249 3257 3259 3260<br>3260 3261
**8**	22 26 33 38 38 40 46 50 57 58 59 64 68 68 71 74 77 80<br>82 87 92 102 107 109 112 119 122 125 128 131 133 138 139 142 144 147 148<br>149 152 155 155 159 161 163 164 165 170 171 173 176 179 179 181 184 186 189<br>190 192 198 202 203 205 207 209 210 212 214 220 222 223 226 227 229 232 236<br>239 255 263 265 272 275 281 292 294 299 301 310 352 355 364 371 373 380 386<br>429 430 504 508 621 622 623 624 625 634 636 641 652 655 660 677 683 686 687<br>688 690 691 691 692 693 693 694 695 695 696 697 698 699 701 703 706 773 775<br>776 778 779 779 780 781 781 783 785 786 788 789 840 845 861 863 863 866 867<br>868 868 870 871 872 873 874 875 877 877 878 879 880 883 884 885 890 891 923<br>945 949 1002 1004 1118 1125 1189 1190 1237 1249 1284<br>1288 1295 1296 1305 1306 1307 1307 1308 1309 1310<br>1310 1311 1312 1312 1314 1314 1316 1318 1319 1320<br>1321 1324 1325 1327 1403 1404 1469 1471 1472 1473<br>1473 1475 1475 1475 1476 1477 1477 1478 1478 1478<br>1479 1480 1481 1482 1483 1484 1484 1484 1486 1487<br>1487 1488 1489 1489 1490 1491 1491 1492 1492 1493<br>1495 1496 1497 1498 1499 1501 1502 1504 1506 1508<br>3049 3050 3050 3050 3052 3052 3053 3053 3054 3054<br>3055 3055 3055 3056 3056 3058 3058 3059 3059 3062<br>3064 3126 3127 3128 3129 3129 3129 3130 3131 3131<br>3132 3132 3133 3133 3134 3134 3135 3136 3137 3138<br>3138 3140 3141 3142 3144 3144 3145 3145 3147 3148<br>3148 3149 3150 3152 3153 3153 3155 3157 3160 3162<br>3164 3172 3177 3219 3242 3254 3255
**DMAP**	43 81 143 197 250 274 386 409 482 545 552 679 754 771 821 837 966 984 991 1006<br>1091 1102 1143 1147 1154 1213 1267 1291 1364 1391<br>1424 1462 1484 1503 1504 1511 1535 1550 1576 1610<br>1672 3025 3033 3083 3084 3167 3170 3173 3176 3241<br>3242
**INT8a**	13 27 31 38 40 45 47 51 54 56 60 66 69 72 80 83 94 104 108 116<br>121 123 125 129 132 136 140 141 144 146 150 150 150 159 161 162 163 164 166<br>168 170 171 172 177 180 182 184 190 192 195 197 201 204 205 207 210 212 214<br>217 221 223 224 227 228 233 259 266 273 276 280 289 294 310 352 357 365 375<br>378 394 429 430 500 504 621 622 623 624 633 636 641 648 653 662 687 687 688<br>688 689 691 691 691 692 693 693 695 695 696 696 697 701 703 747 774 775 777<br>779 780 780 782 783 783 784 786 788 846 853 861 862 866 867 868 868 870 871<br>872 872 873 874 874 875 879 881 881 885 889 897 948 953 1004 1007 1120<br>1122 1177 1184 1243 1249 1285 1288 1295 1296 1301<br>1301 1305 1305 1309 1310 1311 1311 1313 1313 1313<br>1314 1314 1315 1321 1323 1324 1324 1391 1400 1470<br>1471 1472 1474 1475 1476 1477 1477 1478 1478 1478<br>1478 1480 1481 1482 1482 1484 1484 1485 1486 1487<br>1487 1488 1489 1489 1490 1491 1492 1493 1494 1495<br>1496 1497 1499 1500 1501 1506 1511 3046 3048 3049<br>3049 3050 3051 3051 3052 3052 3052 3053 3053 3053<br>3054 3054 3055 3055 3056 3125 3125 3126 3128 3128<br>3130 3130 3130 3131 3131 3131 3131 3132 3133 3133<br>3133 3134 3135 3136 3137 3137 3138 3138 3139 3141<br>3142 3143 3144 3145 3148 3149 3150 3152 3153 3153

	3158 3215 3224 3225 3228
**TS8c**	-87 15 24 28 35 42 44 49 49 51 54 59 61 64 69 70 74 78 87 89<br>94 95 99 108 112 118 127 130 131 133 135 139 141 141 144 145 149 150 154<br>155 158 160 164 164 166 168 170 170 173 175 175 176 180 181 188 189 190 194<br>197 199 205 207 209 210 212 216 220 221 225 228 230 232 233 244 248 252 257<br>264 265 268 272 278 293 302 303 318 334 350 362 366 368 377 387 429 433 462<br>502 504 586 623 624 625 626 637 639 643 645 647 668 685 689 690 692 692 693<br>693 694 694 695 695 696 697 698 699 699 701 704 712 720 729 736 775 776 778<br>780 781 782 783 783 784 786 787 806 812 813 847 862 863 864 869 869 870 871<br>871 872 873 875 877 877 878 880 881 883 884 886 887 892 896 899 909 941 945<br>997 1011 1124 1125 1191 1198 1242 1256 1279 1282<br>1289 1294 1297 1297 1304 1308 1308 1309 1309 1311<br>1312 1313 1313 1315 1315 1316 1320 1321 1321 1322<br>1324 1328 1336 1399 1409 1471 1473 1473 1473 1474<br>1475 1475 1476 1477 1477 1477 1478 1479 1479 1480<br>1481 1481 1481 1482 1483 1483 1485 1486 1487 1487<br>1488 1488 1489 1490 1491 1491 1492 1493 1495 1495<br>1496 1497 1498 1499 1501 1501 1502 1508 1589 3050<br>3050 3051 3052 3053 3053 3053 3054 3055 3055 3055<br>3055 3056 3056 3056 3057 3062 3063 3066 3067 3068<br>3119 3127 3128 3129 3131 3131 3131 3132 3132 3133<br>3133 3133 3134 3134 3135 3136 3136 3139 3140 3140<br>3140 3140 3140 3140 3141 3142 3143 3144 3144 3148<br>3148 3151 3151 3154 3155 3155 3160 3168 3169 3188<br>3189 3191 3223 3234 3243 3268
**TS8d**	-166 17 26 31 38 40 47 50 53 57 58 59 63 64 67 70 75 79 84<br>86 90 99 102 106 114 117 125 126 130 132 133 135 136 141 143 144 147 147<br>150 154 155 161 162 165 166 168 169 174 175 177 179 181 183 185 189 194 195<br>201 204 207 207 209 212 212 213 216 220 221 225 226 226 231 235 239 244 254<br>262 264 266 271 272 283 284 286 290 295 303 336 351 359 367 374 382 389 432<br>434 504 506 621 621 623 624 626 637 638 643 652 655 657 675 681 684 686 687<br>690 690 690 691 692 692 694 694 696 696 697 699 702 705 706 710 723 774 775<br>777 777 779 781 781 782 783 784 787 789 790 804 857 859 860 861 865 866 867<br>868 869 870 871 872 873 873 875 876 878 879 881 883 885 888 892 896 899 945<br>948 1001 1007 1122 1131 1172 1194 1228 1255 1282<br>1293 1295 1299 1302 1304 1305 1306 1307 1307 1308<br>1310 1311 1311 1313 1313 1314 1315 1318 1318 1319<br>1320 1321 1323 1326 1382 1399 1460 1465 1471 1474<br>1474 1475 1476 1477 1477 1477 1478 1479 1479 1479<br>1480 1480 1482 1482 1483 1483 1484 1486 1487 1487<br>1488 1488 1489 1489 1490 1491 1491 1493 1493 1493<br>1495 1497 1498 1499 1499 1501 1502 1504 1507 1508<br>3043 3048 3050 3051 3051 3051 3051 3052 3052 3053<br>3054 3054 3055 3055 3056 3056 3056 3057 3057 3062<br>3064 3121 3126 3126 3127 3128 3128 3129 3130 3130<br>3131 3131 3132 3132 3133 3133 3134 3135 3136 3137<br>3137 3139 3139 3139 3140 3141 3141 3144 3145 3146<br>3147 3147 3151 3153 3155 3157 3160 3161 3163 3167<br>3180 3215 3216 3217 3223 3243 3275

Table S10. The energies, enthalpies and free energies (in au at 298 K) and corresponding relative values with ZPE correction (in kcal/mol) for **5**+**Me_3SiCl**, obtained with B3PW91-PCM/6-31G(d)/ECP80MWB method

species	E	ZPE	G	H	S (sol)[a]
**5**	-3333.33593	0.94845	-3332.48329	-3332.31732	349.3
**Me_3SiCl**	-869.37504	0.11358	-869.28331	-869.25192	66.1
**5+Me_3SiCl**	-4202.71097 (0.0)	1.06203	-4201.76660 (0.0)	-4201.56925 (0.0)	415.4
**TS8a**	-4202.69342 (12.2)	1.06385	-4201.73742 (18.3)	-4201.54964 (12.3)	395.2
**TS8b**	-4202.67913 (21.0)	1.06362	-4201.72192 (28.0)	-4201.53582 (21.0)	391.7
**INT8**	-4202.76288 (-30.7)	1.06494	-4201.80235 (-22.4)	-4201.61809 (-30.6)	387.8
**8**	-3820.65981	0.89900	-3819.85431	-3819.69079	344.1
**DMAP**	-382.11054	0.16303	-381.97098	-381.93790	69.6
**8+DMAP**	-4202.77035 (-37.3)	1.06203	-4201.82529 (-36.8)	-4201.62869 (-37.3)	413.7
**INT8a**	-2951.19137	0.78261	-2950.49257	-2950.34856	303.1
**INT8a+DMAP**[b]	-3333.30191 (19.6)	0.94564	-3332.46355 (12.4)	-3332.28646 (19.4)	372.7
**TS8c**[c]	-3820.556589 (28.1)	0.90000	-3819.74946 (29.0)	-3819.587615 (27.4)	340.6
**TS8d**[c]	-3820.543422 (35.6)	0.898711	-3819.74675 (36.7)	-3819.575554 (35.0)	339.9

a. Values are in $cal\cdot mol^{-1}\cdot K^{-1}$.

b. The relative values are with respect to **5**.

c. The relative values are with respect to **5+Me_3SiCl** by including the corresponding energies of **DMAP.**

Table S11. The optimized Cartesian Coordinates (in Å) and structure (the hydrogen atoms omitted for clarity) of **10** and **10'**, obtained with B3PW91-PCM/6-31G(d)/ECP60MWB method.

Species	Cartesian coordinates				
**10**	U	10.349549	5.691226	4.920044	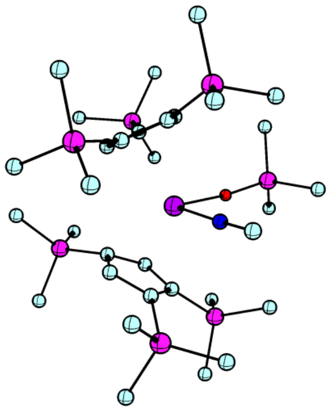 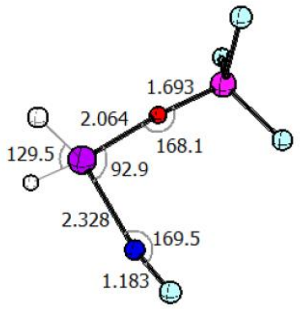
	Si	11.195026	9.761656	5.492591	
	Si	11.817827	8.350778	1.851899	
	Si	6.463594	7.404226	3.496966	
	Si	12.123153	1.780518	4.753128	
	Si	9.647816	2.732928	1.802175	
	Si	7.264326	3.894106	6.975269	
	Si	11.695825	5.906925	8.399174	
	O	10.982970	5.967331	6.864869	
	N	12.523522	5.441616	4.124362	
	C	10.287812	8.433495	4.490210	
	C	10.520923	7.905366	3.165278	
	C	9.328134	7.221135	2.788525	
	H	9.179635	6.736269	1.826731	
	C	8.327745	7.325771	3.800712	
	C	8.961464	8.050481	4.851924	
	H	8.475804	8.338017	5.779988	
	C	10.091204	10.225386	6.959502	
	H	9.157764	10.688833	6.618195	
	H	10.607607	10.957940	7.592512	
	H	9.833369	9.369619	7.591545	
	C	12.893493	9.244264	6.131067	
	H	12.798501	8.438668	6.864149	
	H	13.388805	10.091041	6.622749	
	H	13.549714	8.884722	5.333181	
	C	11.361640	11.338951	4.456581	
	H	12.043488	11.234964	3.607164	
	H	11.744872	12.150003	5.089331	
	H	10.386630	11.654308	4.066528	
	C	13.542476	8.765896	2.494517	
	H	13.571232	9.646259	3.143214	
	H	14.187536	8.976454	1.631433	
	H	13.979155	7.919738	3.034962	
	C	11.125006	9.841566	0.904509	
	H	10.163057	9.592168	0.440513	
	H	11.815024	10.137801	0.104095	
	H	10.965461	10.712076	1.549222	
	C	11.995898	6.944150	0.607594	
	H	12.425318	6.052089	1.072904	
	H	12.667959	7.256361	-0.201630	
	H	11.038509	6.672404	0.148116	
	C	6.215640	8.940938	2.417005	
	H	6.615869	9.836603	2.906145	
	H	5.150477	9.112560	2.216898	
	H	6.725716	8.835529	1.452191	
	C	5.708392	5.948336	2.559018	
	H	6.218493	5.767328	1.607004	
	H	4.663089	6.189112	2.325868	
	H	5.706789	5.010361	3.123088	
	C	5.522513	7.679303	5.112543	
	H	5.550492	6.813496	5.780664	
	H	4.468889	7.884801	4.885243	

	H	5.910546	8.544381	5.663061	
	C	10.572266	2.875224	4.682485	
	C	9.666051	3.262999	3.622609	
	C	8.497213	3.783927	4.252282	
	H	7.617341	4.127444	3.721424	
	C	8.622227	3.736245	5.672842	
	C	9.912294	3.175598	5.904027	
	H	10.326456	2.980891	6.887313	
	C	11.512577	0.031787	5.162339	
	H	10.966409	0.021842	6.113293	
	H	12.358426	-0.661239	5.255018	
	H	10.839596	-0.361236	4.392321	
	C	13.184534	1.710332	3.195460	
	H	12.662654	1.303089	2.324248	
	H	14.041738	1.054936	3.398327	
	H	13.580754	2.697849	2.937565	
	C	13.240851	2.345018	6.167697	
	H	13.667158	3.330649	5.955189	
	H	14.072002	1.637859	6.283360	
	H	12.717538	2.392195	7.129052	
	C	9.565617	0.840840	1.757941	
	H	10.480687	0.370108	2.131655	
	H	9.413469	0.495999	0.727183	
	H	8.729375	0.470753	2.362735	
	C	8.067286	3.375706	0.986733	
	H	7.159475	3.018197	1.485057	
	H	8.031903	3.027691	-0.053348	
	H	8.033430	4.470529	0.965772	
	C	11.094920	3.328050	0.745590	
	H	10.926131	4.352784	0.402004	
	H	11.185380	2.693871	-0.145671	
	H	12.054141	3.308815	1.268506	
	C	7.317795	5.528071	7.929435	
	H	8.243403	5.617649	8.507993	
	H	6.478384	5.586371	8.633820	
	H	7.256636	6.400197	7.269289	
	C	5.584592	3.694615	6.133197	
	H	5.382955	4.480169	5.397624	
	H	4.780273	3.728093	6.878514	
	H	5.521160	2.729342	5.617208	
	C	7.486701	2.473287	8.202686	
	H	7.423620	1.504254	7.694029	
	H	6.703936	2.501283	8.971012	
	H	8.454262	2.517286	8.715274	
	C	11.170000	7.420238	9.389117	
	H	11.545456	8.353051	8.956726	
	H	11.565053	7.346379	10.410411	
	H	10.078681	7.496360	9.462436	
	C	13.567057	5.842844	8.227970	
	H	13.886390	4.919605	7.733282	
	H	14.031902	5.867681	9.221974	
	H	13.966626	6.682333	7.650017	
	C	11.119879	4.373453	9.332625	
	H	10.030307	4.329967	9.434984	
	H	11.543782	4.392900	10.345014	
	H	11.456737	3.445012	8.859469	

	C	13.686760	5.392811	3.912412
10'	U	10.305466	5.708520	4.952047
	Si	11.202306	9.762487	5.488446
	Si	11.826753	8.334767	1.857556
	Si	6.456208	7.420828	3.488006
	Si	12.133869	1.807301	4.749814
	Si	9.642196	2.741731	1.799938
	Si	7.257767	3.860054	6.978983
	Si	11.690054	5.915001	8.419867
	O	10.980004	5.976820	6.881455
	C	12.594347	5.427827	4.052276
	C	10.284375	8.438821	4.488452
	C	10.524964	7.894094	3.170606
	C	9.326964	7.219444	2.790572
	H	9.182340	6.725794	1.832816
	C	8.320368	7.342381	3.793307
	C	8.951903	8.072890	4.842991
	H	8.461029	8.373248	5.764387
	C	10.099608	10.241754	6.951297
	H	9.172470	10.716301	6.608219
	H	10.623784	10.968436	7.584732
	H	9.830414	9.389648	7.583776
	C	12.892242	9.227334	6.135090
	H	12.780116	8.473496	6.919042
	H	13.423208	10.084891	6.567203
	H	13.524645	8.795569	5.353902
	C	11.388498	11.333729	4.447455
	H	12.074870	11.220539	3.602995
	H	11.774983	12.143214	5.080230
	H	10.418834	11.656545	4.050292
	C	13.552343	8.734551	2.504726
	H	13.588083	9.607506	3.163009
	H	14.198959	8.949303	1.643790
	H	13.977845	7.876022	3.034002
	C	11.137854	9.833004	0.917970
	H	10.175796	9.587964	0.451759
	H	11.829457	10.129965	0.119136
	H	10.980238	10.701929	1.565161
	C	11.993912	6.935230	0.604883
	H	12.427007	6.042487	1.064951
	H	12.662016	7.252510	-0.205750
	H	11.034036	6.667006	0.148464
	C	6.208027	8.956483	2.407155
	H	6.608315	9.852699	2.895210
	H	5.142858	9.127856	2.206972
	H	6.717950	8.849892	1.442392
	C	5.704034	5.961428	2.554017
	H	6.217110	5.776111	1.604511
	H	4.659241	6.200336	2.316903
	H	5.701646	5.026465	3.123106
	C	5.519346	7.694616	5.106171
	H	5.558260	6.830974	5.776809
	H	4.462829	7.890090	4.883804
	H	5.902591	8.564733	5.652083
	C	10.577179	2.896917	4.676502
	C	9.661871	3.274420	3.620356

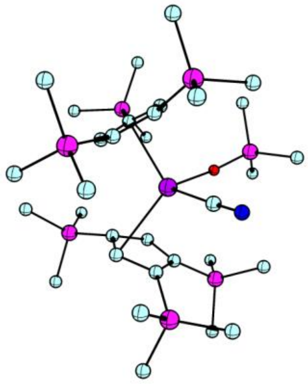

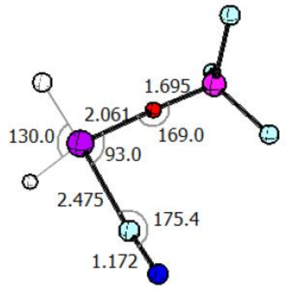

	C 8.488890 3.778621 4.253776<br>H 7.602661 4.111752 3.726574<br>C 8.617106 3.726361 5.674825<br>C 9.915202 3.183608 5.901186<br>H 10.333103 2.989301 6.883130<br>C 11.521575 0.067169 5.194230<br>H 10.976573 0.074659 6.145868<br>H 12.366705 -0.625173 5.297894<br>H 10.847223 -0.339037 4.432069<br>C 13.183246 1.706829 3.187359<br>H 12.652596 1.294030 2.324083<br>H 14.034145 1.044480 3.394628<br>H 13.586403 2.688668 2.919752<br>C 13.256218 2.401984 6.147631<br>H 13.661173 3.392956 5.918513<br>H 14.101284 1.711162 6.261098<br>H 12.741782 2.448680 7.113828<br>C 9.548698 0.849818 1.768966<br>H 10.456693 0.377300 2.157416<br>H 9.406346 0.497225 0.739452<br>H 8.703518 0.489497 2.367259<br>C 8.064322 3.388344 0.982109<br>H 7.154502 3.038165 1.481975<br>H 8.027234 3.034526 -0.055962<br>H 8.036058 4.483176 0.954500<br>C 11.092883 3.324560 0.743488<br>H 10.930187 4.349161 0.396887<br>H 11.180368 2.686785 -0.145518<br>H 12.051143 3.302324 1.267798<br>C 7.312524 5.478820 7.957869<br>H 8.264812 5.590554 8.487124<br>H 6.511631 5.496990 8.707920<br>H 7.184061 6.359094 7.318567<br>C 5.578561 3.678354 6.132806<br>H 5.379956 4.479733 5.413432<br>H 4.773561 3.698774 6.877789<br>H 5.513104 2.724036 5.597163<br>C 7.484656 2.421501 8.184056<br>H 7.427440 1.459805 7.661105<br>H 6.700738 2.434486 8.951553<br>H 8.451398 2.463154 8.698514<br>C 11.144415 7.419428 9.412177<br>H 11.508873 8.358032 8.982879<br>H 11.538511 7.348117 10.433992<br>H 10.052099 7.481994 9.483873<br>C 13.561078 5.867028 8.249182<br>H 13.885223 4.958869 7.730243<br>H 14.025142 5.868010 9.243842<br>H 13.956346 6.723563 7.693835<br>C 11.123646 4.370551 9.340260<br>H 10.034054 4.314424 9.435966<br>H 11.541280 4.388492 10.355269<br>H 11.473834 3.448882 8.863538<br>N 13.709826 5.318667 3.710445	

Table S12. Frequencies of the stationary points optimized for **10 and 10'**, obtained with B3PW91-PCM/6-31G(d)/ECP60MWB method.

Species	Frequencies (cm^{-1})
**10**	28 28 33 35 40 43 48 50 55 59 59 63 66 67 69 72 75 77<br>82 88 90 94 102 106 109 115 118 120 125 128 132 134 136 138 143 143 146<br>146 147 149 151 154 156 159 161 167 168 170 170 175 176 180 181 181 183 185<br>188 188 189 201 202 203 205 208 210 210 212 213 214 222 225 226 228 231 233<br>235 244 247 264 267 272 275 287 294 295 297 301 308 336 352 357 366 370 374<br>377 383 428 430 505 511 618 621 625 625 627 630 637 641 649 654 658 685 685<br>687 689 690 690 691 692 693 693 693 694 695 696 699 699 700 700 701 706 711<br>774 776 778 779 779 780 781 782 783 784 784 788 788 790 840 848 856 861 864<br>865 867 868 869 870 871 872 873 874 875 876 877 879 880 881 883 883 886 887<br>895 902 940 943 998 999 1119 1127 1189 1190 1236 1247 1291<br>1292 1301 1302 1305 1307 1307 1307 1308 1309 1309<br>1310 1311 1312 1314 1315 1316 1317 1318 1320 1321<br>1323 1325 1325 1329 1403 1406 1468 1469 1472 1473<br>1474 1474 1475 1475 1476 1476 1477 1478 1478 1479<br>1479 1480 1481 1482 1483 1484 1484 1485 1485 1487<br>1487 1488 1488 1490 1491 1491 1492 1492 1493 1494<br>1495 1496 1497 1499 1499 1500 1501 1503 1507 1511<br>2128 3049 3050 3051 3052 3053 3054 3054 3055 3055<br>3055 3056 3056 3056 3057 3057 3058 3058 3059 3059<br>3065 3065 3128 3128 3128 3130 3130 3130 3131 3131<br>3132 3132 3133 3133 3133 3134 3134 3135 3136 3136<br>3138 3140 3141 3142 3145 3146 3147 3148 3148 3148<br>3149 3150 3151 3154 3155 3156 3156 3157 3158 3161<br>3162 3164 3172 3178 3212 3231 3248 3256
**10′**	28 32 36 39 42 45 48 55 57 58 61 65 67 67 70 71 74 76<br>81 87 89 92 100 103 110 113 118 123 125 128 129 134 134 138 142 143 144<br>147 150 151 153 155 156 158 160 166 168 168 170 176 178 179 181 182 186 186<br>190 200 201 201 203 204 208 211 211 213 214 215 220 224 225 227 232 232 236<br>246 248 253 264 267 273 276 286 295 298 300 303 311 313 352 357 365 369 376<br>377 389 428 431 507 513 618 621 625 625 627 631 636 641 648 654 662 685 686<br>687 689 690 691 691 692 693 694 694 694 695 697 699 699 700 701 701 706 712<br>774 776 778 779 779 780 781 782 783 784 785 787 787 790 840 846 859 861 864<br>865 867 868 870 871 871 872 873 874 875 877 878 880 881 881 884 884 887 889<br>897 902 940 944 997 998 1121 1127 1190 1191 1236 1248 1291<br>1292 1298 1300 1304 1306 1307 1307 1308 1309 1310<br>1310 1311 1313 1313 1315 1316 1317 1318 1320 1321<br>1323 1325 1325 1328 1402 1407 1468 1469 1471 1473<br>1473 1474 1475 1475 1476 1476 1477 1478 1478 1479<br>1480 1480 1481 1482 1483 1484 1484 1485 1485 1486<br>1487 1488 1488 1490 1491 1491 1491 1493 1493 1494<br>1495 1496 1497 1498 1499 1501 1501 1505 1507 1511<br>2229 3049 3049 3051 3052 3053 3054 3054 3055 3055<br>3055 3055 3056 3056 3057 3057 3058 3059 3059 3059<br>3065 3066 3127 3128 3129 3129 3130 3130 3131 3131<br>3132 3132 3133 3133 3133 3134 3134 3135 3136 3138<br>3138 3140 3140 3141 3145 3147 3147 3148 3148 3148<br>3149 3149 3151 3154 3156 3157 3157 3157 3158 3164<br>3165 3168 3172 3180 3214 3231 3246 3254

Table S13. The energies, enthalpies and free energies (in au at 298 K) and corresponding relative values with ZPE correction (in kcal/mol) for **10** and **10'**, obtained with B3PW91-PCM/6-31G(d)/ECP60MWB method

**species**	**E**	**ZPE**	**G**	**H**	**S (sol)**[a]
**10**	-3892.817584 (0.0)	0.904991	-3892.00806 (0.0)	-3891.841438 (0.0)	350.7
**10′**	-3892.817434 (0.4)	0.905531	-3892.00676 (0.8)	-3891.840912 (0.3)	349.1

a. Values are in cal·mol^{-1}·K^{-1}.

Table S14. The optimized Cartesian Coordinates (in Å) and structures (the hydrogen atoms omitted for clarity) of stationary points for **5**+PhNCS, obtained with B3PW91-PCM/6-31G(d)/ECP80MWB method.

Species	Cartesian coordinates				
**5**	U	6.650204	16.809966	4.699887	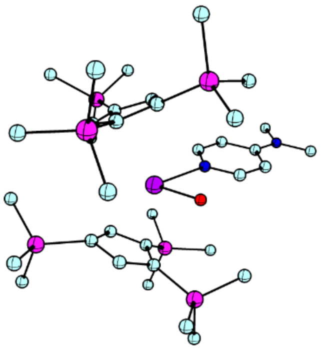 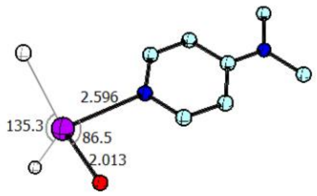
	Si	10.800925	16.720879	4.253365	
	Si	8.828948	13.278501	4.513175	
	Si	6.562856	16.533691	0.280039	
	Si	2.665749	18.688062	5.108848	
	Si	5.155349	18.391011	8.228124	
	Si	7.646715	21.138690	3.849132	
	O	7.078392	15.880492	6.433610	
	N	4.647183	15.171072	4.498137	
	N	1.540715	12.345574	4.443685	
	C	9.157773	16.150298	3.508713	
	C	8.459894	14.883959	3.588647	
	C	7.418171	14.937698	2.621940	
	H	6.731837	14.120744	2.420766	
	C	7.446830	16.168476	1.894257	
	C	8.517112	16.901945	2.469144	
	H	8.843693	17.881298	2.136313	
	C	11.432302	18.171594	3.208722	
	H	11.604582	17.863246	2.170589	
	H	12.387642	18.531090	3.611325	
	H	10.744703	19.024189	3.193040	
	C	10.687084	17.283282	6.057422	
	H	10.059549	18.176194	6.168893	
	H	11.686229	17.536435	6.435166	
	H	10.264962	16.506661	6.703841	
	C	12.127287	15.375650	4.128379	
	H	11.925330	14.514099	4.771890	
	H	13.096692	15.792775	4.430602	
	H	12.225823	15.012978	3.098653	
	C	10.107191	12.271292	3.532956	
	H	9.745847	12.078583	2.515144	
	H	10.275836	11.299825	4.015979	
	H	11.075278	12.775168	3.450838	
	C	7.257580	12.218621	4.534686	
	H	6.437461	12.713067	5.064577	
	H	7.463033	11.270402	5.047921	
	H	6.911388	11.973103	3.523578	
	C	9.389380	13.534241	6.294025	
	H	10.378732	13.997592	6.374737	
	H	9.430827	12.572405	6.821283	
	H	8.660683	14.188570	6.788724	
	C	5.761843	14.936767	-0.349120	
	H	6.513166	14.151296	-0.491555	
	H	5.270953	15.108871	-1.315032	
	H	5.003516	14.546440	0.340369	
	C	5.225079	17.870224	0.408529	
	H	4.417193	17.597947	1.097764	
	H	4.771562	18.048793	-0.574878	
	H	5.647543	18.820621	0.754003	
	C	7.841859	17.121070	-0.984555	
	H	8.329911	18.047253	-0.658201	
	H	7.374353	17.317589	-1.957519	
	H	8.624727	16.368003	-1.131188	

	C	4.543080	18.798681	5.241798	
	C	5.420598	18.787866	6.398241	
	C	6.644307	19.374271	5.975773	
	H	7.500707	19.536162	6.623374	
	C	6.572451	19.796367	4.607822	
	C	5.272705	19.416831	4.179430	
	H	4.868370	19.611116	3.189566	
	C	2.159081	18.341690	3.315261	
	H	2.594709	19.060178	2.611757	
	H	1.068026	18.414527	3.221315	
	H	2.448573	17.334676	2.996417	
	C	1.839753	17.377846	6.194240	
	H	2.199169	16.367950	5.968798	
	H	0.757009	17.395150	6.013804	
	H	1.995057	17.559865	7.262384	
	C	1.948133	20.383044	5.564462	
	H	2.204871	20.673523	6.588845	
	H	0.853948	20.382739	5.477830	
	H	2.338289	21.159002	4.895120	
	C	4.668926	16.607709	8.613921	
	H	3.742297	16.293998	8.122404	
	H	4.533890	16.482011	9.696107	
	H	5.482678	15.955573	8.277153	
	C	3.847927	19.564532	8.950567	
	H	4.109632	20.612618	8.761810	
	H	3.780775	19.427380	10.037703	
	H	2.848474	19.391368	8.536402	
	C	6.779772	18.731630	9.137116	
	H	7.582645	18.089732	8.757594	
	H	6.657254	18.513511	10.205592	
	H	7.098582	19.776761	9.047614	
	C	6.691803	22.768566	4.009019	
	H	5.754149	22.736603	3.441286	
	H	7.283698	23.612662	3.632637	
	H	6.437401	22.974442	5.055379	
	C	9.257477	21.309767	4.824570	
	H	9.884763	22.097878	4.390109	
	H	9.847081	20.387127	4.837706	
	H	9.053750	21.589372	5.865114	
	C	8.009273	20.888600	2.008191	
	H	7.085689	20.728756	1.439015	
	H	8.672918	20.039461	1.815440	
	H	8.493177	21.784480	1.599059	
	C	3.894967	14.959934	3.400130	
	H	4.143459	15.554222	2.525401	
	C	2.864305	14.047261	3.328758	
	H	2.318914	13.950826	2.398049	
	C	2.544669	13.256685	4.462512	
	C	3.345277	13.477131	5.610844	
	H	3.190936	12.916103	6.524280	
	C	4.357501	14.414525	5.579589	
	H	4.993263	14.585038	6.442893	
	C	0.756752	12.150140	3.235969	
	H	0.003599	11.383435	3.418913	
	H	1.383554	11.817383	2.398705	
	H	0.238637	13.071282	2.940369	

	C	1.251964	11.555157	5.628998	
	H	2.110176	10.935386	5.918263	
	H	0.411156	10.893238	5.420393	
	H	0.979670	12.193367	6.478935	
**PhNCS**	C	-0.424426	-0.000001	-4.663272	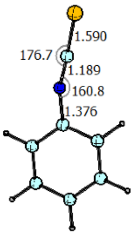
	C	0.726345	-0.000000	-5.462188	
	C	-1.698936	-0.000001	-5.250131	
	C	0.596496	0.000000	-6.847418	
	C	-1.810049	0.000000	-6.636382	
	C	-0.667480	0.000001	-7.438539	
	H	1.703045	-0.000001	-4.988628	
	H	-2.580270	-0.000001	-4.616168	
	H	1.488823	0.000001	-7.467182	
	H	-2.796679	0.000000	-7.091544	
	H	-0.762674	0.000001	-8.520686	
	N	-0.293451	-0.000002	-3.293776	
	C	-0.576197	-0.000002	-2.138918	
	S	-0.865014	0.000004	-0.575497	
**TS13**	U	7.425854	17.234510	4.673562	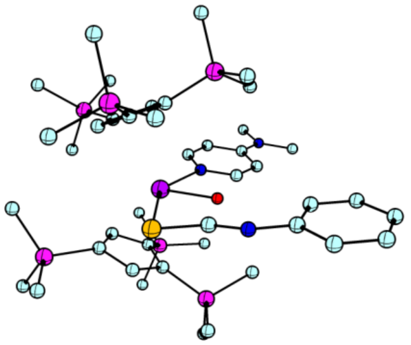
	Si	11.550593	16.858781	3.149052	
	Si	9.627664	13.546333	4.342656	
	Si	6.682162	16.263462	0.296456	
	Si	2.975121	18.292451	4.867572	
	Si	5.424961	19.146158	7.870888	
	Si	7.395009	21.302310	2.934691	
	O	7.979665	16.532158	6.530607	
	N	5.573681	15.393673	4.941709	
	N	2.857839	12.258368	5.614060	
	C	9.728431	16.343816	3.088084	
	C	9.053609	15.126478	3.485282	
	C	7.837828	15.081200	2.742787	
	H	7.133654	14.256606	2.783334	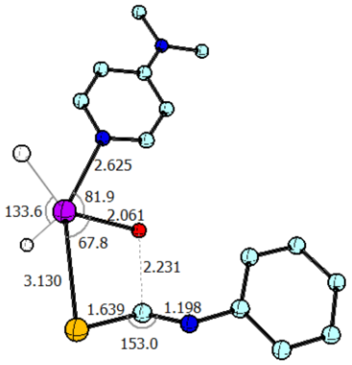
	C	7.723905	16.196897	1.860375	
	C	8.887388	16.973913	2.117598	
	H	9.144996	17.886600	1.591372	
	C	11.703856	18.622904	2.473426	
	H	11.373856	18.695192	1.430650	
	H	12.755971	18.933158	2.504712	
	H	11.131914	19.346557	3.064790	
	C	12.469412	16.769228	4.809053	
	H	12.856031	17.759656	5.074120	
	H	13.324616	16.086003	4.737533	
	H	11.841051	16.435180	5.636769	
	C	12.474671	15.732734	1.930600	
	H	12.501019	14.692947	2.276645	
	H	13.513766	16.067907	1.814936	
	H	12.004307	15.742757	0.940432	
	C	10.270423	12.374054	2.993385	
	H	9.488718	12.169295	2.251626	
	H	10.582504	11.414237	3.425167	
	H	11.128481	12.793946	2.456601	
	C	8.161573	12.679689	5.166643	
	H	7.802790	13.248075	6.030243	
	H	8.473384	11.688366	5.520027	
	H	7.315830	12.535732	4.484632	
	C	10.974700	13.743735	5.648347	

	H 11.930504 14.068338 5.226289				
	H 11.141544 12.772436 6.131923				
	H 10.682632 14.456479 6.426401				
	C 5.970170 14.535470 -0.016613				
	H 6.768831 13.788882 -0.096104				
	H 5.404364 14.522534 -0.956482				
	H 5.290106 14.208344 0.779113				
	C 5.245768 17.496315 0.278150				
	H 4.505628 17.289549 1.058211				
	H 4.729734 17.443517 -0.689252				
	H 5.592783 18.527234 0.409268				
	C 7.833936 16.714764 -1.136443				
	H 8.261712 17.716264 -1.007904				
	H 7.293725 16.705398 -2.091472				
	H 8.666554 16.005926 -1.211156				
	C 4.812954 18.719683 4.881990				
	C 5.643610 19.157527 5.992498				
	C 6.703281 19.911390 5.421901				
	H 7.477239 20.406544 5.995845				
	C 6.588705 19.977315 3.998302				
	C 5.418452 19.225745 3.694383				
	H 5.006954 19.102655 2.697487				
	C 2.424815 17.609260 3.186345				
	H 2.711741 18.272828 2.362748				
	H 1.329173 17.540522 3.177055				
	H 2.814908 16.609605 2.972838				
	C 2.400168 17.086587 6.206319				
	H 2.915662 16.122925 6.143135				
	H 1.325038 16.901062 6.084545				
	H 2.550140 17.481618 7.216234				
	C 2.023271 19.921271 5.071177				
	H 2.295042 20.450630 5.989566				
	H 0.940845 19.739578 5.091728				
	H 2.234393 20.591829 4.229425				
	C 5.367027 17.425915 8.646935				
	H 4.537446 16.816798 8.273213				
	H 5.264173 17.505472 9.736806				
	H 6.305184 16.907925 8.419759				
	C 3.871825 20.108209 8.387773				
	H 3.869814 21.117649 7.959431				
	H 3.853852 20.210765 9.480779				
	H 2.939901 19.616361 8.089974				
	C 6.895056 20.061930 8.635439				
	H 7.849969 19.588155 8.383504				
	H 6.800529 20.055246 9.728735				
	H 6.941543 21.109462 8.315151				
	C 6.043806 22.557982 2.498406				
	H 5.255637 22.096719 1.890966				
	H 6.453027 23.402839 1.930084				
	H 5.571179 22.956054 3.404020				
	C 8.742894 22.177031 3.931497				
	H 9.212625 22.962629 3.326655				
	H 9.533581 21.488823 4.251673				
	H 8.333149 22.652026 4.830404				
	C 8.144341 20.684371 1.310399				
	H 7.456960 20.033825 0.757931				

	H 9.074107 20.131070 1.478854<br>H 8.382266 21.538369 0.663460<br>C 4.759365 14.936552 3.972919<br>H 4.852222 15.420102 3.005123<br>C 3.852801 13.910068 4.137735<br>H 3.244489 13.612522 3.292370<br>C 3.736245 13.270981 5.398574<br>C 4.596491 13.756542 6.413083<br>H 4.592245 13.330411 7.408892<br>C 5.476127 14.786150 6.140307<br>H 6.162879 15.160238 6.893980<br>C 2.000968 11.795521 4.536531<br>H 1.364178 10.991438 4.906379<br>H 2.586627 11.404360 3.694463<br>H 1.351349 12.598977 4.166346<br>C 2.779267 11.629975 6.921545<br>H 3.731949 11.162389 7.202016<br>H 2.014680 10.852897 6.899105<br>H 2.504641 12.354209 7.699012<br>C 10.025856 16.377177 9.170667<br>C 10.891147 16.498698 10.265821<br>C 9.079799 15.342933 9.115305<br>C 10.806477 15.582439 11.309844<br>C 9.012328 14.435778 10.168006<br>C 9.869209 14.549361 11.265040<br>H 11.616090 17.306826 10.284464<br>H 8.432401 15.304869 8.242839<br>H 11.476450 15.675939 12.160395<br>H 8.282157 13.630891 10.131893<br>H 9.807641 13.834925 12.081775<br>N 10.130946 17.292116 8.142559<br>C 9.757441 17.753403 7.102498<br>S 9.854402 18.814209 5.857227	
**13**	U 4.386650 6.877430 6.468283<br>S 6.345344 6.008144 8.337728<br>Si 6.180419 2.918073 6.170191<br>Si 7.235840 5.737627 3.535873<br>Si 1.559718 4.986858 3.574345<br>Si 4.290058 10.081168 9.289880<br>Si 1.273865 10.066658 6.691875<br>Si 1.689147 4.999254 9.320124<br>O 6.377000 8.058633 6.752560<br>N 8.150126 8.112576 8.154865<br>N 4.508537 8.870261 4.712028<br>N 4.711551 11.946249 1.848519<br>C 5.214214 4.348583 5.379260<br>C 5.587036 5.328874 4.380048<br>C 4.368021 5.814779 3.821665<br>H 4.320528 6.525780 3.004943<br>C 3.233176 5.198457 4.423979<br>C 3.788232 4.315276 5.404468<br>H 3.202641 3.636217 6.015332<br>C 5.399030 2.413703 7.815103<br>H 4.336079 2.166703 7.716454<br>H 5.506357 3.198924 8.570558<br>H 5.905525 1.517081 8.194273	

	C	5.965095	1.459016	4.974227	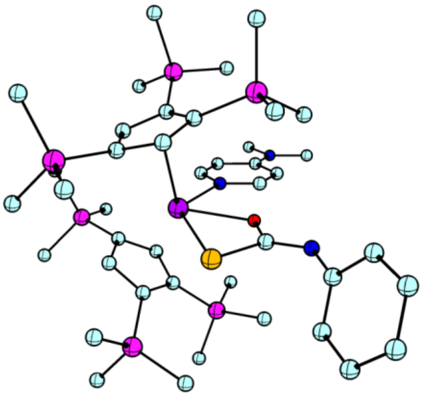 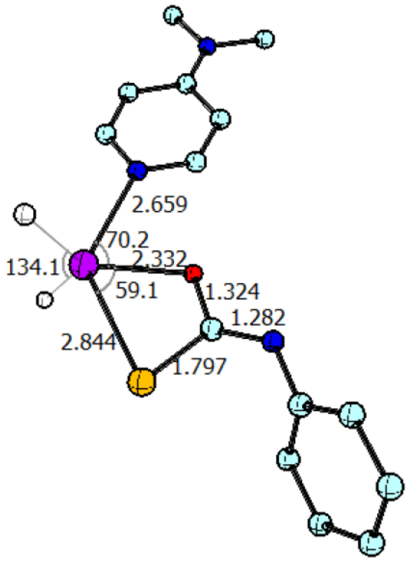
	H	6.476059	0.566888	5.358394	
	H	6.377035	1.679306	3.982916	
	H	4.905577	1.208012	4.843571	
	C	8.024330	3.186755	6.453628	
	H	8.438571	2.276251	6.906153	
	H	8.192242	4.014848	7.148406	
	H	8.584103	3.377156	5.532954	
	C	8.577457	6.407859	4.674665	
	H	8.256591	7.336026	5.158163	
	H	9.484739	6.617858	4.093608	
	H	8.844210	5.703095	5.466978	
	C	6.893707	7.044827	2.207113	
	H	6.200742	6.683835	1.437848	
	H	7.835084	7.301260	1.704764	
	H	6.484657	7.969445	2.629178	
	C	7.860622	4.197103	2.623981	
	H	8.118003	3.373635	3.298075	
	H	8.760697	4.444203	2.046506	
	H	7.103965	3.827872	1.921200	
	C	1.531689	6.000962	1.976258	
	H	1.513685	7.082225	2.156195	
	H	0.626888	5.755514	1.406155	
	H	2.394351	5.778333	1.338001	
	C	0.032961	5.449239	4.586759	
	H	0.008008	4.948507	5.560296	
	H	-0.868166	5.144564	4.039289	
	H	-0.035403	6.528674	4.755073	
	C	1.429468	3.152772	3.127495	
	H	2.275476	2.836998	2.506133	
	H	0.505489	2.947640	2.572631	
	H	1.426114	2.523820	4.025562	
	C	3.316674	8.677230	8.455582	
	C	2.282341	8.647123	7.434094	
	C	1.746851	7.324419	7.430916	
	H	0.919565	6.997682	6.809737	
	C	2.375993	6.516862	8.429834	
	C	3.340009	7.379016	9.036554	
	H	3.999254	7.082193	9.842481	
	C	5.272253	9.318200	10.711566	
	H	5.970475	8.550138	10.360844	
	H	4.620475	8.868443	11.469839	
	H	5.861054	10.100633	11.206957	
	C	3.086884	11.328982	10.061461	
	H	3.651452	12.028091	10.692104	
	H	2.348490	10.829485	10.699715	
	H	2.544457	11.924646	9.320019	
	C	5.495925	11.011014	8.173761	
	H	6.351402	10.373699	7.926020	
	H	5.871771	11.894834	8.705741	
	H	5.039887	11.353101	7.239288	
	C	0.006700	10.592063	8.000590	
	H	-0.647107	9.750394	8.258957	
	H	-0.626764	11.399862	7.611943	
	H	0.472036	10.945446	8.925590	
	C	2.273638	11.576663	6.158139	

	H	2.798252	12.044362	6.997015	
	H	1.591513	12.327524	5.738851	
	H	3.015630	11.333499	5.391060	
	C	0.234442	9.469805	5.223748	
	H	0.825207	9.115668	4.374086	
	H	-0.383347	10.304109	4.867353	
	H	-0.452509	8.669246	5.521318	
	C	2.909886	4.472267	10.659633	
	H	3.895607	4.230432	10.246911	
	H	2.538622	3.581439	11.181243	
	H	3.047423	5.259895	11.409140	
	C	1.290897	3.514030	8.219410	
	H	0.711211	3.789833	7.331967	
	H	0.691790	2.793465	8.790524	
	H	2.196200	2.994948	7.889515	
	C	0.070883	5.556029	10.132198	
	H	0.237393	6.414678	10.793088	
	H	-0.365150	4.747503	10.732395	
	H	-0.671829	5.853503	9.382041	
	C	7.091673	7.526264	7.731842	
	C	8.991325	7.582127	9.130889	
	C	9.678808	6.367648	8.957276	
	H	9.508696	5.793422	8.051551	
	C	10.580420	5.914642	9.917087	
	H	11.099609	4.971692	9.758231	
	C	10.822921	6.658983	11.071967	
	H	11.527682	6.303273	11.819428	
	C	10.155810	7.872514	11.248790	
	H	10.338573	8.469247	12.140104	
	C	9.259148	8.333495	10.288800	
	H	8.748843	9.284247	10.417800	
	C	5.503828	9.783030	4.714705	
	H	6.246584	9.653493	5.494087	
	C	5.609407	10.809408	3.797046	
	H	6.448918	11.488087	3.885197	
	C	4.644904	10.954327	2.770862	
	C	3.604557	9.990109	2.772014	
	H	2.815692	10.004504	2.029927	
	C	3.588222	9.004608	3.736422	
	H	2.795347	8.263628	3.726571	
	C	3.692779	12.055855	0.818559	
	H	3.659103	11.157444	0.189142	
	H	3.923183	12.907388	0.177871	
	H	2.697174	12.216075	1.252168	
	C	5.795079	12.913736	1.900071	
	H	5.791047	13.471281	2.845208	
	H	5.675075	13.628134	1.085284	
	H	6.772245	12.428124	1.785682	
**TS6**	U	-0.073723	-0.238845	0.236355	
	S	1.569815	-1.079549	2.043898	
	Si	1.498794	-4.201454	-0.088497	
	Si	2.884083	-1.509532	-2.678846	
	Si	-2.770391	-1.847887	-3.013902	
	Si	-0.431352	2.685569	3.293302	
	Si	-3.204188	3.169566	0.498911	
	Si	-3.280155	-2.167153	2.397954	

O	2.222600	1.178633	0.017271
N	4.138854	1.170292	1.408413
N	-0.094528	1.915774	-1.540379
N	-0.444636	4.718797	-4.670710
C	0.679180	-2.723720	-0.945245
C	1.178732	-1.757795	-1.897389
C	0.034042	-1.180898	-2.529075
H	0.088789	-0.452089	-3.329625
C	-1.171632	-1.727332	-2.019584
C	-0.743726	-2.649711	-1.004555
H	-1.409848	-3.299411	-0.449339
C	0.567365	-4.669981	1.483675
H	-0.501119	-4.838836	1.306624
H	0.677853	-3.875232	2.228131
H	0.980944	-5.598094	1.898756
C	1.314436	-5.655395	-1.301113
H	1.752458	-6.568241	-0.876865
H	1.807980	-5.462547	-2.260182
H	0.256973	-5.859552	-1.509584
C	3.324630	-4.001079	0.335127
H	3.671648	-4.910727	0.842613
H	3.457037	-3.155321	1.017545
H	3.961702	-3.851617	-0.542515
C	4.293884	-1.043855	-1.504797
H	4.407075	0.044553	-1.455438
H	5.242922	-1.455910	-1.869922
H	4.135794	-1.413583	-0.488194
C	2.741851	-0.129376	-3.971402
H	2.050974	-0.388783	-4.782001
H	3.726138	0.048052	-4.423417
H	2.407035	0.816023	-3.528352
C	3.356441	-3.091445	-3.611813
H	3.559411	-3.930613	-2.937830
H	4.261518	-2.921267	-4.209262
H	2.555607	-3.398723	-4.294766
C	-2.433979	-1.137822	-4.737721
H	-2.155071	-0.077827	-4.708350
H	-3.330342	-1.225643	-5.364260
H	-1.622278	-1.679734	-5.236417
C	-4.314702	-0.986015	-2.338734
H	-4.617992	-1.373568	-1.360124
H	-5.147372	-1.165052	-3.031470
H	-4.191145	0.098343	-2.249688
C	-3.175492	-3.688418	-3.182070
H	-2.339403	-4.234998	-3.633236
H	-4.059356	-3.837523	-3.814895
H	-3.383392	-4.146304	-2.207790
C	-1.403469	1.457972	2.227748
C	-2.302852	1.618943	1.092951
C	-2.855710	0.340100	0.828032
H	-3.600127	0.138861	0.066034
C	-2.380536	-0.631292	1.773608
C	-1.498984	0.098847	2.628227
H	-0.955144	-0.335652	3.456487
C	0.396818	1.726176	4.691893
H	1.016730	0.911692	4.298895

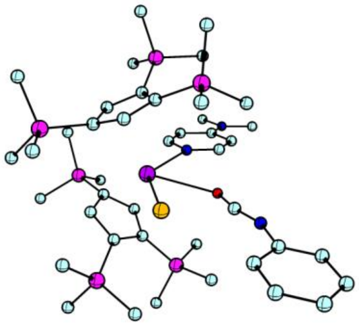

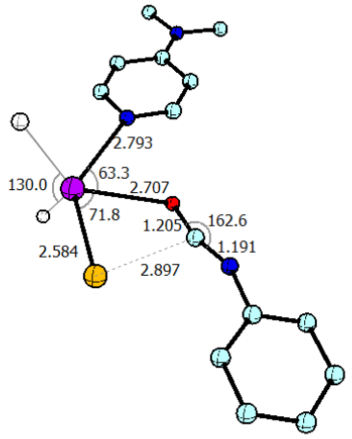

	H	-0.339544	1.294470	5.380337	
	H	1.040995	2.397617	5.273772	
	C	-1.567761	3.973881	4.098700	
	H	-0.986610	4.554147	4.827412	
	H	-2.392806	3.495227	4.638996	
	H	-1.997109	4.682762	3.383304	
	C	0.911452	3.626456	2.341583	
	H	1.777737	2.983759	2.154851	
	H	1.261281	4.480287	2.936070	
	H	0.560919	4.012007	1.378527	
	C	-4.480895	3.615957	1.830256	
	H	-5.202364	2.798404	1.948632	
	H	-5.039542	4.514191	1.536303	
	H	-4.032111	3.803861	2.809541	
	C	-2.115023	4.678102	0.161157	
	H	-1.562243	5.001435	1.049090	
	H	-2.748980	5.517690	-0.152152	
	H	-1.389609	4.491251	-0.637775	
	C	-4.243993	2.826111	-1.047470	
	H	-3.648087	2.627901	-1.942283	
	H	-4.860426	3.710869	-1.253369	
	H	-4.929797	1.985201	-0.894133	
	C	-2.286695	-2.930814	3.809519	
	H	-1.255692	-3.144661	3.507354	
	H	-2.746599	-3.871797	4.135960	
	H	-2.248220	-2.262056	4.677095	
	C	-3.667544	-3.501115	1.110193	
	H	-4.006823	-3.082359	0.156569	
	H	-4.472027	-4.142484	1.491975	
	H	-2.806047	-4.147057	0.911784	
	C	-4.945172	-1.556145	3.070912	
	H	-4.801043	-0.773207	3.824583	
	H	-5.505174	-2.376466	3.537886	
	H	-5.570165	-1.136560	2.273154	
	C	3.120830	1.031788	0.806623	
	C	5.092378	0.793557	2.344384	
	C	4.901051	-0.322504	3.167007	
	H	3.981292	-0.893986	3.072451	
	C	5.892347	-0.660287	4.084133	
	H	5.747262	-1.526177	4.724788	
	C	7.059751	0.098063	4.185928	
	H	7.827171	-0.175761	4.905035	
	C	7.238149	1.209546	3.361874	
	H	8.143924	1.805571	3.435230	
	C	6.257332	1.563039	2.439591	
	H	6.381810	2.424695	1.790585	
	C	0.769391	2.940169	-1.688659	
	H	1.570164	2.995637	-0.962705	
	C	0.695994	3.888346	-2.691878	
	H	1.446086	4.669623	-2.717742	
	C	-0.338557	3.823676	-3.655463	
	C	-1.258512	2.760645	-3.485167	
	H	-2.099829	2.625701	-4.154430	
	C	-1.091640	1.869657	-2.443361	
	H	-1.800235	1.055784	-2.321574	
	C	-1.533230	4.610306	-5.625879	

	H	-1.506786	3.650847	-6.158213	
	H	-1.441943	5.407569	-6.364080	
	H	-2.510907	4.710694	-5.136913	
	C	0.524698	5.793030	-4.797103	
	H	0.513495	6.452656	-3.919694	
	H	0.280317	6.393425	-5.673821	
	H	1.541972	5.402066	-4.926281	
**PhNCO**	C	-0.358707	0.000004	-4.496943	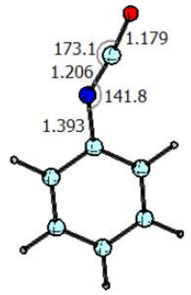
	C	0.752516	0.000003	-5.346227	
	C	-1.653105	0.000002	-5.033874	
	C	0.567095	0.000000	-6.726163	
	C	-1.824757	-0.000001	-6.414903	
	C	-0.718917	-0.000002	-7.266284	
	H	1.748373	0.000004	-4.913906	
	H	-2.512553	0.000004	-4.368847	
	H	1.434133	-0.000001	-7.381282	
	H	-2.830768	-0.000002	-6.826122	
	H	-0.859595	-0.000004	-8.343541	
	N	-0.136085	0.000007	-3.121371	
	C	-0.720479	0.000008	-2.066309	
	O	-1.163571	-0.000016	-0.974251	
**6**	U	6.670518	16.960065	4.586220	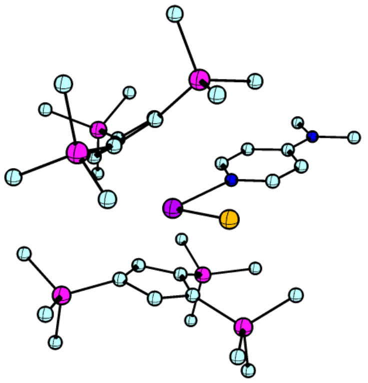 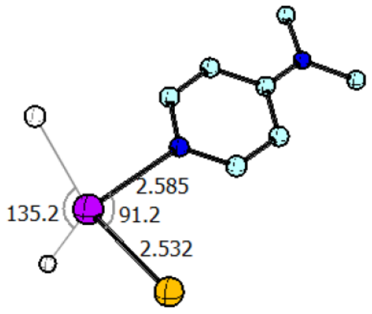
	Si	10.882996	16.808998	3.837489	
	Si	8.894736	13.385750	4.223330	
	Si	6.455360	16.663972	0.161466	
	Si	2.553870	18.752633	4.663554	
	Si	4.741113	18.611072	7.993877	
	Si	7.634973	21.162859	3.793045	
	S	7.439603	16.001953	6.800135	
	N	4.680916	15.311827	4.504234	
	N	1.536102	12.529093	4.308823	
	C	9.137679	16.304674	3.299070	
	C	8.442485	15.039955	3.418371	
	C	7.346422	15.096252	2.510283	
	H	6.653721	14.277101	2.340647	
	C	7.326798	16.332612	1.794466	
	C	8.426742	17.068633	2.320030	
	H	8.737774	18.045597	1.967620	
	C	11.350224	18.387955	2.897793	
	H	11.347510	18.222331	1.813805	
	H	12.365028	18.694178	3.181802	
	H	10.685785	19.231692	3.111167	
	C	11.090307	17.117586	5.688772	
	H	10.633337	18.067998	5.989286	
	H	12.155569	17.165754	5.949282	
	H	10.618254	16.333439	6.288219	
	C	12.118970	15.495568	3.253657	
	H	12.001275	14.531757	3.757565	
	H	13.143579	15.843067	3.439001	
	H	12.014764	15.323355	2.175679	
	C	9.762182	12.350793	2.886082	
	H	9.111332	12.213792	2.013739	
	H	10.019334	11.355478	3.270977	
	H	10.686929	12.823005	2.536069	
	C	7.311189	12.466519	4.700400	
	H	6.802895	12.988409	5.517732	

	H	7.558280	11.454254	5.045324	
	H	6.607576	12.366286	3.865624	
	C	9.989899	13.462722	5.756429	
	H	10.976932	13.899236	5.576492	
	H	10.144687	12.440875	6.127633	
	H	9.491657	14.043780	6.539633	
	C	5.584075	15.079515	-0.399497	
	H	6.298128	14.253798	-0.499576	
	H	5.112453	15.232644	-1.378064	
	H	4.800580	14.755896	0.295741	
	C	5.194806	18.074240	0.210115	
	H	4.362446	17.870923	0.892623	
	H	4.770720	18.237024	-0.788946	
	H	5.663770	19.013149	0.525921	
	C	7.778674	17.117828	-1.112254	
	H	8.309854	18.033297	-0.825723	
	H	7.332601	17.287965	-2.100109	
	H	8.522992	16.319076	-1.209129	
	C	4.420896	18.847463	4.940814	
	C	5.191027	18.879797	6.170913	
	C	6.446430	19.459946	5.841910	
	H	7.240129	19.644917	6.558227	
	C	6.508713	19.807439	4.453515	
	C	5.246091	19.408884	3.922465	
	H	4.935929	19.560179	2.892710	
	C	2.159351	18.390854	2.846105	
	H	2.650321	19.093269	2.163608	
	H	1.077515	18.490763	2.690278	
	H	2.438820	17.374080	2.552235	
	C	1.632587	17.476229	5.709537	
	H	2.024419	16.463500	5.566221	
	H	0.575085	17.470635	5.414839	
	H	1.672219	17.705420	6.778960	
	C	1.836630	20.470175	5.023881	
	H	2.026152	20.792966	6.052530	
	H	0.750878	20.477055	4.862929	
	H	2.278632	21.219554	4.356338	
	C	4.132921	16.884265	8.463796	
	H	3.316089	16.521441	7.832298	
	H	3.774885	16.894081	9.501754	
	H	4.967395	16.178724	8.392992	
	C	3.398951	19.862602	8.488486	
	H	3.675628	20.882349	8.195257	
	H	3.272148	19.852261	9.578956	
	H	2.422170	19.639769	8.045031	
	C	6.271848	18.987005	9.034067	
	H	7.089963	18.309527	8.763753	
	H	6.043174	18.834274	10.096508	
	H	6.613949	20.021869	8.914692	
	C	6.694555	22.794721	4.004414	
	H	5.782549	22.809951	3.395724	
	H	7.314249	23.648703	3.702618	
	H	6.397717	22.945717	5.048857	
	C	9.206111	21.249801	4.839909	
	H	9.898825	21.998310	4.436250	
	H	9.737809	20.293482	4.884034	

	H	8.969257	21.543496	5.869439	
	C	8.050248	21.001306	1.954550	
	H	7.145094	20.854683	1.353248	
	H	8.737280	20.177870	1.736732	
	H	8.526561	21.926028	1.604931	
	C	3.950248	15.132084	3.384730	
	H	4.223362	15.745070	2.530973	
	C	2.909067	14.237252	3.266696	
	H	2.386682	14.170789	2.320289	
	C	2.548260	13.427954	4.374318	
	C	3.317077	13.620752	5.548578	
	H	3.126511	13.050832	6.449465	
	C	4.343708	14.542482	5.563899	
	H	4.946559	14.686082	6.454355	
	C	0.775073	12.377727	3.080075	
	H	0.010084	11.614688	3.225077	
	H	1.415976	12.061864	2.247143	
	H	0.273477	13.312949	2.800224	
	C	1.201401	11.722448	5.471190	
	H	2.049252	11.100700	5.784630	
	H	0.371857	11.061282	5.219615	
	H	0.894066	12.347863	6.318851	

Table S15. Frequencies of the stationary points optimized for **5**+PhNCS, obtained with B3PW91-PCM/6-31G(d)/ECP80MWB method.

Species	Frequencies (cm^{-1})
**5**	16 21 24 31 34 36 41 45 52 53 54 58 61 63 66 67 73 80 81<br>83 87 95 99 101 109 110 119 124 126 127 132 134 138 140 141 145 146 148<br>151 153 154 156 159 164 166 167 169 170 174 176 176 179 179 183 187 187 195<br>198 199 202 204 207 209 211 212 217 219 219 223 224 226 228 234 237 250 264<br>264 265 272 275 283 286 293 295 299 352 361 367 376 380 386 406 426 433 436<br>488 500 508 552 564 623 623 623 624 636 638 644 648 656 661 675 680 683 685<br>686 687 688 689 689 690 690 691 692 694 695 696 699 707 710 729 751 773 775<br>777 778 778 779 779 780 781 781 783 790 795 834 841 845 850 862 863 864 866<br>867 868 869 871 871 872 873 873 874 876 877 879 885 888 894 899 949 952 980<br>989 1006 1008 1016 1025 1090 1100 1121 1131 1148<br>1148 1151 1182 1190 1215 1247 1250 1267 1289 1289<br>1289 1296 1301 1302 1303 1304 1305 1306 1306 1308<br>1309 1310 1311 1312 1312 1315 1317 1319 1320 1321<br>1323 1372 1386 1393 1401 1434 1465 1473 1474 1474<br>1475 1476 1476 1477 1478 1478 1478 1480 1480 1481<br>1481 1482 1483 1483 1485 1485 1486 1487 1489 1489<br>1490 1491 1491 1492 1493 1493 1494 1496 1497 1498<br>1499 1500 1501 1503 1505 1506 1508 1515 1518 1533<br>1547 1589 1594 1687 3039 3042 3047 3047 3047 3048<br>3048 3049 3050 3050 3051 3051 3052 3052 3052 3054<br>3054 3054 3056 3056 3103 3107 3121 3122 3125 3126<br>3126 3127 3128 3128 3129 3129 3129 3130 3131 3131<br>3131 3132 3132 3133 3136 3136 3138 3139 3139 3141<br>3141 3146 3146 3146 3147 3148 3148 3150 3152 3152<br>3155 3157 3179 3189 3216 3218 3227 3236 3239 3241<br>3258 3260
**PhNCS**	36 60 248 362 394 414 436 479 506 628 698 699 770 846 926 949 975 1003<br>1016 1059 1112 1191 1203 1306 1342 1377 1499 1541<br>1645 1665 2226 3207 3216 3226 3234 3238
**TS13**	-300 11 15 19 23 24 29 31 36 42 47 48 49 52 56 58 60 61<br>64 66 68 75 80 82 82 86 87 103 104 109 112 115 117 119 122 123 127<br>129 131 135 136 137 141 146 146 149 152 155 156 161 163 166 167 170 173 174<br>176 178 180 181 183 185 187 188 192 192 195 199 200 205 208 209 210 211 215<br>218 222 227 229 231 237 243 253 262 263 264 269 270 277 284 289 297 299 304<br>316 353 360 363 371 377 380 396 402 421 425 432 436 468 487 490 503 506 522<br>552 562 622 623 624 625 626 636 639 647 649 654 654 660 676 681 682 683 684<br>688 689 689 690 691 692 693 694 695 695 697 701 702 706 717 723 751 774 775<br>776 777 777 778 778 779 781 783 783 785 786 791 830 835 840 845 861 863 866<br>866 866 867 868 869 870 870 872 873 874 875 876 880 882 885 887 891 894 910<br>944 945 948 981 989 989 1002 1004 1014 1020 1022 1024 1058<br>1091 1102 1105 1122 1130 1147 1148 1150 1183 1189<br>1197 1197 1213 1242 1252 1262 1279 1281 1289 1295<br>1297 1300 1300 1302 1303 1304 1306 1307 1309 1310<br>1311 1312 1313 1314 1316 1319 1320 1320 1321 1327<br>1344 1373 1377 1383 1392 1404 1432 1465 1473 1474<br>1475 1475 1475 1476 1477 1479 1479 1479 1480 1480<br>1481 1481 1482 1483 1484 1485 1486 1487 1487 1488<br>1489 1489 1489 1491 1491 1492 1493 1495 1495 1496<br>1497 1497 1498 1498 1500 1501 1502 1506 1511 1511<br>1515 1533 1536 1546 1589 1595 1642 1664 1687 2107<br>3039 3046 3047 3047 3048 3049 3049 3050 3051 3052<br>3052 3053 3053 3054 3055 3055 3055 3057 3058 3063<br>3100 3102 3124 3125 3126 3127 3128 3128 3129 3129

	3129 3129 3130 3132 3132 3133 3134 3135 3135 3136<br>3137 3137 3138 3139 3140 3141 3142 3145 3149 3149<br>3152 3152 3156 3156 3158 3160 3162 3176 3182 3186<br>3197 3207 3212 3221 3222 3226 3230 3232 3232 3245<br>3254 3256 3263
**13**	18 22 24 26 30 31 34 39 47 50 53 56 58 60 62 65 66 68 72<br>73 80 82 84 86 90 95 101 107 108 110 118 121 122 126 127 130 131 136<br>141 142 142 147 148 151 154 159 161 165 166 166 169 172 174 174 177 180 183<br>184 186 186 188 190 192 195 196 199 201 204 207 208 210 213 215 216 219 221<br>226 228 229 235 240 241 260 265 266 272 274 275 282 297 301 305 315 361 362<br>364 371 377 381 403 411 421 427 429 439 470 493 503 506 507 544 554 565 598<br>621 623 624 624 627 636 638 641 646 647 658 660 679 683 685 689 689 690 691<br>691 692 693 694 695 696 696 697 698 700 705 707 711 712 751 771 775 776 778<br>779 779 781 782 784 784 786 790 790 791 832 843 846 847 857 863 865 867 868<br>870 870 871 872 872 875 875 877 877 878 882 886 888 891 895 900 907 910 943<br>946 959 980 984 992 999 1002 1011 1026 1037 1058 1092 1102<br>1105 1120 1121 1149 1149 1163 1175 1182 1190 1195<br>1200 1216 1240 1242 1280 1283 1290 1292 1294 1297<br>1304 1305 1306 1306 1308 1310 1310 1311 1312 1313<br>1313 1315 1317 1318 1321 1321 1323 1324 1327 1331<br>1372 1373 1400 1401 1401 1440 1467 1472 1474 1474<br>1474 1476 1476 1476 1477 1477 1478 1479 1479 1480<br>1480 1481 1482 1485 1485 1485 1486 1487 1488 1488<br>1489 1489 1490 1490 1492 1493 1493 1494 1495 1497<br>1497 1498 1500 1502 1503 1504 1505 1506 1509 1515<br>1533 1539 1549 1591 1598 1637 1659 1684 1691 3042<br>3048 3049 3049 3051 3051 3051 3052 3052 3052 3053<br>3053 3056 3057 3057 3058 3059 3060 3060 3060 3102<br>3107 3127 3127 3127 3128 3128 3129 3129 3130 3130<br>3132 3132 3135 3135 3135 3137 3137 3138 3139 3139<br>3140 3141 3143 3145 3145 3147 3147 3148 3154 3156<br>3158 3158 3158 3160 3163 3169 3171 3180 3184 3189<br>3191 3208 3216 3225 3236 3239 3243 3247 3256 3257<br>3260 3271
**TS6**	-50 15 19 24 29 31 33 35 37 42 46 50 54 58 60 63 64 69<br>71 73 76 78 83 85 86 88 94 96 99 102 106 109 111 118 119 123 127<br>132 133 135 140 143 143 147 150 153 153 157 160 160 165 167 168 169 170 178<br>179 182 183 185 188 192 193 195 198 200 201 202 207 208 210 212 214 215 217<br>221 224 231 232 234 235 240 243 264 265 267 269 270 273 284 288 291 297 312<br>341 350 362 367 374 375 380 398 417 419 429 429 432 436 479 493 505 508 509<br>534 550 563 620 621 623 623 628 634 635 643 646 659 659 681 681 683 686 687<br>687 688 691 693 696 696 698 698 698 699 702 703 705 705 709 752 755 774 774<br>775 776 777 779 781 782 783 783 784 788 788 789 830 839 847 859 860 862 862<br>866 867 868 869 870 871 872 873 875 875 878 878 879 880 884 887 890 893 939<br>940 944 977 983 987 995 1000 1003 1008 1016 1019 1057 1096<br>1103 1112 1125 1129 1141 1149 1150 1163 1189 1194<br>1204 1208 1219 1237 1247 1274 1278 1287 1292 1294<br>1297 1301 1301 1302 1304 1305 1306 1308 1310 1310<br>1310 1311 1312 1314 1315 1318 1321 1322 1324 1346<br>1370 1379 1394 1402 1411 1439 1468 1471 1473 1475<br>1476 1476 1477 1478 1479 1479 1479 1480 1481 1481<br>1481 1483 1483 1483 1484 1486 1487 1488 1489 1489<br>1490 1490 1491 1492 1492 1494 1494 1496 1496 1497<br>1498 1498 1499 1500 1501 1503 1506 1506 1508 1512<br>1515 1534 1550 1566 1592 1600 1648 1668 1687 2364<br>3040 3043 3046 3046 3046 3049 3050 3050 3050 3050

	3052 3052 3053 3055 3055 3056 3057 3057 3058 3060<br>3100 3102 3124 3126 3126 3126 3127 3128 3128 3129<br>3130 3130 3130 3132 3133 3133 3133 3134 3134 3136<br>3136 3136 3137 3138 3139 3140 3145 3147 3150 3151<br>3152 3154 3156 3158 3160 3164 3165 3169 3182 3189<br>3200 3208 3214 3223 3223 3231 3248 3251 3252 3259<br>3264 3274 3281
**PHNCO**	62 86 245 387 419 464 508 559 624 634 703 771 774 846 921 972 999 1016<br>1057 1111 1159 1190 1205 1342 1377 1498 1502 1583<br>1649 1671 2391 3202 3209 3218 3227 3234
**6**	15 21 22 29 35 38 41 46 51 56 57 58 61 64 68 71 74 82 84<br>85 87 94 101 104 105 111 115 122 124 128 131 132 136 138 140 142 144 146<br>149 150 151 153 158 161 162 165 168 169 172 174 174 177 178 180 182 185 186<br>189 195 196 199 204 206 208 209 212 214 217 220 223 225 227 233 234 244 263<br>267 270 271 271 281 288 295 296 303 351 361 362 372 375 380 382 410 427 429<br>433 492 502 509 550 568 621 622 622 624 633 638 641 648 655 660 676 681 684<br>685 686 687 690 690 692 693 693 693 693 695 697 697 698 702 712 751 772 774<br>777 777 778 779 781 782 783 784 785 790 791 823 830 842 846 862 863 864 867<br>867 868 870 871 871 872 873 874 874 875 878 879 882 885 889 895 943 946 981<br>983 1001 1003 1012 1029 1091 1102 1119 1121 1147<br>1149 1158 1185 1191 1217 1242 1242 1280 1286 1289<br>1289 1291 1297 1302 1302 1303 1304 1306 1306 1308<br>1309 1309 1311 1311 1312 1312 1315 1319 1320 1321<br>1325 1373 1397 1398 1402 1441 1467 1470 1473 1474<br>1475 1475 1475 1476 1477 1477 1478 1478 1479 1480<br>1480 1482 1482 1484 1485 1485 1486 1487 1487 1489<br>1489 1490 1491 1491 1491 1492 1493 1495 1496 1497<br>1499 1501 1503 1503 1506 1506 1507 1513 1516 1533<br>1548 1589 1597 1688 3043 3046 3047 3048 3048 3049<br>3050 3050 3051 3051 3052 3052 3053 3053 3054 3055<br>3055 3056 3057 3057 3104 3108 3123 3125 3126 3126<br>3127 3127 3128 3128 3130 3130 3130 3131 3131 3132<br>3133 3133 3134 3135 3137 3138 3139 3139 3139 3144<br>3145 3145 3148 3151 3152 3154 3154 3156 3156 3161<br>3161 3162 3181 3190 3218 3226 3228 3239 3244 3251<br>3258 3262

Table S16. The energies, enthalpies and free energies (in au at 298 K) and corresponding relative values with ZPE correction (in kcal/mol) for **5**+PhNCS, obtained with B3PW91-PCM //B3PW91-PCM/6-31G(d)/ECP80MWB method.

species	E	ZPE	G	H	S (sol)
**5**	-3332.38749	0.94845	-3332.48329	-3332.31732	349.3
**PhNCS**	-722.40740	0.10191	-722.43077	-722.39890	67.1
**5 + PhNCS**	-4054.79489 (0.0)	1.05036	-4054.91406 (0.0)	-4054.71623 (0.0)	416.4
**TS13**	-4054.76990 (15.7)	1.05116	-4054.87789 (22.7)	-4054.69118 (15.7)	393.0
**13**	-4054.84499 (-31.4)	1.05495	-4054.94940 (-22.2)	-4054.76711 (-31.9)	383.7
**TS6**	-4054.79619 (-0.8)	1.05361	-4054.90177 (7.7)	-4054.71782 (-1.0)	387.2
**PhNCO**	-399.46800	0.10437	-399.48958	-399.45994	62.4
**6**	-3655.35336	0.94755	-3655.44964	-3655.28278	351.2
**6 + PhNCO**	-4054.82136 (-16.6)	1.05192	-4054.93922 (-15.8)	-4054.74272 (-16.6)	413.6

Table S17. The optimized Cartesian Coordinates (in Å) and structure (the hydrogen atoms omitted for clarity) of **5**-**7**, obtained with B3PW91-PCM/6-31G(d)/ECP60MWB method.

Species	Cartesian coordinates				
**5**	U	6.641385	16.815247	4.680999	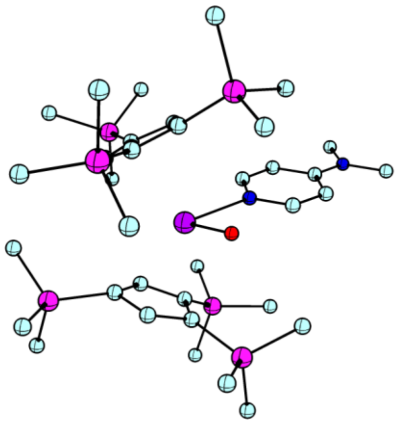 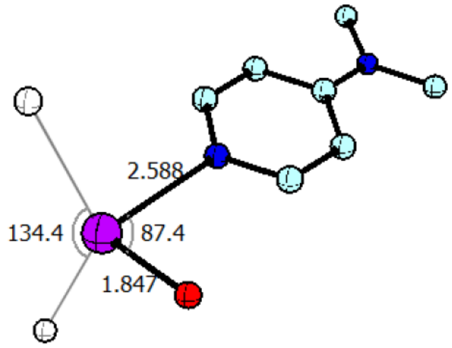
	Si	10.745655	16.687375	4.360159	
	Si	8.747923	13.263901	4.466245	
	Si	6.578759	16.664503	0.315731	
	Si	2.684409	18.653005	5.137609	
	Si	5.255866	18.412610	8.187090	
	Si	7.630787	21.066974	3.721518	
	O	7.084126	16.011777	6.284075	
	N	4.661953	15.155166	4.530635	
	N	1.547144	12.331212	4.471237	
	C	9.121253	16.155519	3.559212	
	C	8.399632	14.900236	3.594653	
	C	7.374823	14.996127	2.616659	
	H	6.676569	14.198446	2.382145	
	C	7.423572	16.251075	1.940805	
	C	8.504203	16.950102	2.547708	
	H	8.849148	17.932169	2.245644	
	C	11.409551	18.181602	3.401491	
	H	11.605394	17.925140	2.353411	
	H	12.356030	18.516196	3.844485	
	H	10.724265	19.035805	3.413476	
	C	10.578421	17.172862	6.181953	
	H	9.957654	18.069069	6.302375	
	H	11.564650	17.394750	6.609933	
	H	10.117588	16.376278	6.774593	
	C	12.078781	15.349494	4.205521	
	H	11.860105	14.449492	4.787956	
	H	13.037168	15.748486	4.562504	
	H	12.211741	15.049640	3.159344	
	C	10.010277	12.272153	3.451803	
	H	9.629761	12.090840	2.438941	
	H	10.196470	11.295769	3.917910	
	H	10.971927	12.786168	3.355828	
	C	7.166518	12.219453	4.493188	
	H	6.386017	12.676981	5.109548	
	H	7.388884	11.230832	4.915191	
	H	6.755618	12.061142	3.489105	
	C	9.338928	13.445362	6.250737	
	H	10.334941	13.892573	6.333881	
	H	9.379650	12.460563	6.733802	
	H	8.637346	14.080035	6.802704	
	C	5.929519	15.056303	-0.446820	
	H	6.740060	14.331759	-0.586810	
	H	5.478956	15.249994	-1.428235	
	H	5.164088	14.579500	0.177370	
	C	5.124321	17.874262	0.449583	
	H	4.327689	17.509201	1.108292	
	H	4.681269	18.038346	-0.541255	
	H	5.446555	18.849765	0.830776	
	C	7.855527	17.430958	-0.850848	
	H	8.250633	18.371834	-0.450192	

	H	7.411708	17.648054	-1.830520	
	H	8.703460	16.753819	-1.006053	
	C	4.563595	18.758338	5.217873	
	C	5.473078	18.763860	6.343682	
	C	6.691375	19.328109	5.873240	
	H	7.567993	19.491434	6.493230	
	C	6.588436	19.712094	4.501492	
	C	5.272841	19.329169	4.119240	
	H	4.841689	19.503560	3.136973	
	C	2.123262	18.263190	3.369385	
	H	2.550383	18.953731	2.633506	
	H	1.031173	18.350057	3.302875	
	H	2.389601	17.242602	3.073957	
	C	1.877494	17.375827	6.276877	
	H	2.222898	16.358205	6.064361	
	H	0.790489	17.398070	6.124248	
	H	2.061135	17.580845	7.336355	
	C	1.984916	20.361813	5.572453	
	H	2.272553	20.675718	6.581703	
	H	0.888637	20.363726	5.517987	
	H	2.357687	21.120634	4.874109	
	C	4.758173	16.644394	8.637455	
	H	3.829759	16.319763	8.157104	
	H	4.619753	16.562542	9.723366	
	H	5.561208	15.963821	8.336492	
	C	3.978459	19.608100	8.925325	
	H	4.244553	20.650407	8.712620	
	H	3.937548	19.489213	10.015903	
	H	2.967828	19.437537	8.538250	
	C	6.905607	18.752463	9.051764	
	H	7.696937	18.097124	8.670354	
	H	6.804317	18.559210	10.127251	
	H	7.232272	19.792139	8.932473	
	C	6.569607	22.638721	3.722015	
	H	5.682475	22.520818	3.087928	
	H	7.136827	23.500442	3.347446	
	H	6.222035	22.876170	4.734292	
	C	9.154080	21.392111	4.796297	
	H	9.772967	22.180697	4.350540	
	H	9.785858	20.505389	4.917724	
	H	8.860650	21.729828	5.797338	
	C	8.164511	20.770977	1.928515	
	H	7.330139	20.425549	1.307154	
	H	8.974563	20.039524	1.842091	
	H	8.526982	21.712267	1.495769	
	C	3.926264	14.924024	3.426142	
	H	4.189221	15.503072	2.544960	
	C	2.893843	14.012466	3.351242	
	H	2.364101	13.901481	2.413010	
	C	2.552957	13.241768	4.491836	
	C	3.332606	13.482384	5.649884	
	H	3.159306	12.940395	6.571534	
	C	4.346114	14.419999	5.617206	
	H	4.953778	14.601977	6.496990	
	C	0.784001	12.116951	3.253802	
	H	0.021588	11.359736	3.438395	

	H	1.422843	11.763187	2.434216	
	H	0.278188	13.035631	2.930512	
	C	1.242236	11.556190	5.662050	
	H	2.098011	10.943866	5.974109	
	H	0.407599	10.887781	5.449031	
	H	0.953461	12.204402	6.499087	
**6**	U	6.614473	16.932394	4.534852	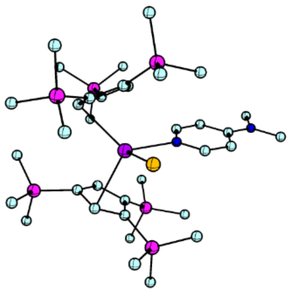 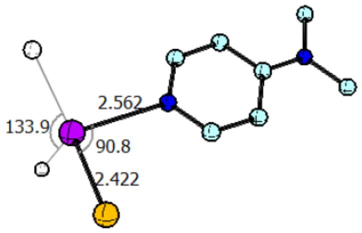
	Si	10.802865	16.846421	3.946286	
	Si	8.836146	13.405571	4.203033	
	Si	6.440544	16.755210	0.193329	
	Si	2.607710	18.702828	4.709205	
	Si	4.889281	18.569100	7.974569	
	Si	7.635062	21.105983	3.689414	
	S	7.379483	16.009787	6.639214	
	N	4.668963	15.267345	4.469300	
	N	1.488849	12.520666	4.294304	
	C	9.076397	16.346677	3.352261	
	C	8.389775	15.073916	3.424662	
	C	7.306182	15.148422	2.507412	
	H	6.619675	14.331532	2.305927	
	C	7.274536	16.412154	1.846103	
	C	8.371252	17.136963	2.399130	
	H	8.667543	18.131864	2.089189	
	C	11.305494	18.427972	3.030116	
	H	11.305101	18.276605	1.943957	
	H	12.324308	18.711227	3.323414	
	H	10.655438	19.280069	3.252074	
	C	10.957770	17.152382	5.802334	
	H	10.375763	18.030084	6.106733	
	H	12.006481	17.332109	6.072646	
	H	10.585217	16.306737	6.387822	
	C	12.062798	15.540621	3.396491	
	H	11.924295	14.568119	3.877262	
	H	13.077862	15.885037	3.633194	
	H	12.007388	15.387532	2.311949	
	C	9.753106	12.405192	2.872306	
	H	9.129866	12.280516	1.978240	
	H	10.006118	11.404324	3.245382	
	H	10.684141	12.890664	2.559148	
	C	7.249705	12.457125	4.611118	
	H	6.708431	12.946548	5.427413	
	H	7.499649	11.437883	4.932894	
	H	6.572316	12.373548	3.753171	
	C	9.886010	13.447827	5.770907	
	H	10.886475	13.865030	5.622860	
	H	10.009653	12.419244	6.135255	
	H	9.382597	14.030422	6.549265	
	C	5.642898	15.152519	-0.425719	
	H	6.386072	14.350685	-0.505486	
	H	5.210614	15.306863	-1.422203	
	H	4.838650	14.793983	0.227628	
	C	5.124232	18.114935	0.201015	
	H	4.278063	17.879084	0.855287	
	H	4.728169	18.251976	-0.813466	
	H	5.539053	19.076958	0.522469	
	C	7.792149	17.268110	-1.026914	

	H	8.287744	18.193111	-0.709172	
	H	7.374587	17.440293	-2.026807	
	H	8.561221	16.491448	-1.109336	
	C	4.481355	18.776228	4.938136	
	C	5.290233	18.806037	6.136761	
	C	6.544455	19.372389	5.763540	
	H	7.364355	19.545849	6.452610	
	C	6.561757	19.722779	4.380900	
	C	5.287202	19.312463	3.890610	
	H	4.949905	19.449055	2.867002	
	C	2.160004	18.301961	2.912513	
	H	2.631069	18.990711	2.202633	
	H	1.074101	18.399951	2.785968	
	H	2.430919	17.279007	2.632029	
	C	1.684894	17.474018	5.810039	
	H	2.042323	16.447492	5.675102	
	H	0.619336	17.494229	5.546408	
	H	1.762106	17.722308	6.873207	
	C	1.927506	20.441662	5.041784	
	H	2.146217	20.787132	6.057347	
	H	0.838522	20.463294	4.905787	
	H	2.366186	21.166740	4.345711	
	C	4.243546	16.870205	8.496817	
	H	3.425740	16.504614	7.868440	
	H	3.874586	16.922579	9.529663	
	H	5.059753	16.141731	8.458693	
	C	3.594760	19.865472	8.480613	
	H	3.893725	20.871493	8.162762	
	H	3.491585	19.877738	9.573492	
	H	2.602888	19.663338	8.061475	
	C	6.453624	18.927387	8.971860	
	H	7.259035	18.242697	8.683304	
	H	6.251854	18.780907	10.040530	
	H	6.803198	19.958098	8.839375	
	C	6.574462	22.675782	3.762049	
	H	5.704737	22.600081	3.098308	
	H	7.153157	23.557081	3.457284	
	H	6.201407	22.850943	4.777877	
	C	9.134098	21.365165	4.813162	
	H	9.793974	22.137071	4.398340	
	H	9.730012	20.456315	4.948344	
	H	8.818077	21.702844	5.807344	
	C	8.179418	20.906389	1.886882	
	H	7.352209	20.579928	1.245914	
	H	9.005600	20.200083	1.759997	
	H	8.521559	21.877521	1.506909	
	C	3.959196	15.061444	3.341714	
	H	4.260525	15.642345	2.474473	
	C	2.907880	14.176803	3.228027	
	H	2.404655	14.086320	2.273209	
	C	2.513299	13.407445	4.352350	
	C	3.260416	13.625644	5.535907	
	H	3.043164	13.086289	6.449630	
	C	4.298313	14.535847	5.543121	
	H	4.883443	14.699659	6.441258	
	C	0.751040	12.340311	3.055735	

	H	-0.031469	11.597141	3.210805	
	H	1.402624	11.983911	2.247633	
	H	0.273154	13.274144	2.732983	
	C	1.122212	11.753443	5.473174	
	H	1.956480	11.132289	5.822581	
	H	0.290373	11.093751	5.225086	
	H	0.805340	12.407240	6.295697	
**7**	U	6.604862	16.958717	4.514874	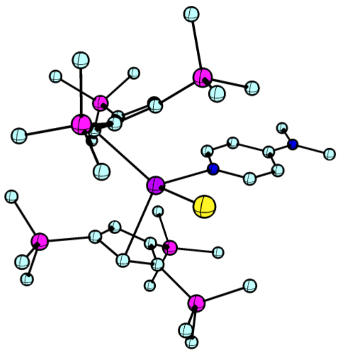 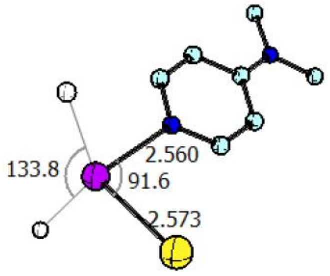
	Si	10.814819	16.861053	3.872656	
	Si	8.833054	13.429192	4.168127	
	Si	6.426285	16.779074	0.177883	
	Si	2.592433	18.713881	4.632945	
	Si	4.815498	18.604669	7.934453	
	Si	7.644704	21.114643	3.693160	
	Se	7.451298	16.007507	6.751002	
	N	4.665279	15.288527	4.454605	
	N	1.490574	12.536067	4.280985	
	C	9.066215	16.376904	3.326430	
	C	8.380002	15.103823	3.404286	
	C	7.287226	15.179425	2.497608	
	H	6.600667	14.361479	2.300453	
	C	7.246700	16.444833	1.840260	
	C	8.349450	17.169217	2.384875	
	H	8.642887	18.164367	2.073267	
	C	11.293312	18.457541	2.969889	
	H	11.253946	18.328106	1.881643	
	H	12.323880	18.728240	3.232490	
	H	10.657858	19.309085	3.232377	
	C	11.056797	17.124442	5.725894	
	H	10.537161	18.025667	6.071536	
	H	12.124479	17.244435	5.952014	
	H	10.666839	16.287820	6.312697	
	C	12.037251	15.556223	3.239909	
	H	11.918357	14.577484	3.713537	
	H	13.065376	15.891769	3.427910	
	H	11.924086	15.418997	2.157758	
	C	9.674953	12.420162	2.795261	
	H	9.012420	12.309168	1.928077	
	H	9.928629	11.414030	3.153276	
	H	10.599163	12.893831	2.445709	
	C	7.253724	12.505439	4.650780	
	H	6.767710	13.004914	5.495292	
	H	7.501144	11.480679	4.956596	
	H	6.530851	12.438753	3.829301	
	C	9.955853	13.453285	5.684548	
	H	10.950328	13.867967	5.495387	
	H	10.089590	12.421246	6.035149	
	H	9.488627	14.032122	6.488191	
	C	5.611059	15.180669	-0.428516	
	H	6.341815	14.365792	-0.488089	
	H	5.194400	15.328629	-1.432580	
	H	4.792449	14.844495	0.218654	
	C	5.131531	18.158162	0.156554	
	H	4.273155	17.938286	0.800029	
	H	4.752489	18.292303	-0.864767	
	H	5.555161	19.116982	0.476126	

	C	7.796834	17.255897	-1.035741	
	H	8.301042	18.179016	-0.726283	
	H	7.391315	17.418839	-2.042108	
	H	8.556604	16.468391	-1.099351	
	C	4.463092	18.791617	4.888503	
	C	5.250791	18.826336	6.100992	
	C	6.511443	19.391989	5.747422	
	H	7.318158	19.568134	6.451030	
	C	6.553570	19.735123	4.364035	
	C	5.287549	19.321678	3.852408	
	H	4.967128	19.456017	2.823186	
	C	2.169048	18.319057	2.829317	
	H	2.640037	19.018228	2.129713	
	H	1.083578	18.406760	2.692151	
	H	2.453494	17.301225	2.544353	
	C	1.662403	17.476078	5.716979	
	H	2.027440	16.452417	5.580617	
	H	0.599870	17.491722	5.441203	
	H	1.726419	17.718600	6.782250	
	C	1.903157	20.450557	4.956498	
	H	2.105212	20.798348	5.974483	
	H	0.816356	20.468562	4.803514	
	H	2.350060	21.175590	4.265568	
	C	4.190623	16.900104	8.461021	
	H	3.375658	16.524793	7.834428	
	H	3.825550	16.947008	9.495435	
	H	5.018379	16.184623	8.415915	
	C	3.487173	19.882967	8.398257	
	H	3.770814	20.890445	8.071098	
	H	3.367950	19.908457	9.489294	
	H	2.505519	19.655273	7.968613	
	C	6.347913	19.010226	8.963064	
	H	7.173304	18.333926	8.713775	
	H	6.118017	18.883847	10.028673	
	H	6.682554	20.044065	8.816845	
	C	6.602682	22.695526	3.787233	
	H	5.732610	22.638931	3.122068	
	H	7.192033	23.573834	3.494505	
	H	6.231382	22.861589	4.805211	
	C	9.143949	21.335013	4.824517	
	H	9.830269	22.085095	4.412713	
	H	9.709649	20.408090	4.966811	
	H	8.831568	21.684649	5.815712	
	C	8.184599	20.933519	1.887447	
	H	7.353566	20.621324	1.244275	
	H	9.005954	20.223986	1.748576	
	H	8.532055	21.907408	1.519550	
	C	3.952462	15.084708	3.328461	
	H	4.250618	15.668484	2.462111	
	C	2.902971	14.198070	3.215393	
	H	2.397301	14.109114	2.261708	
	C	2.513345	13.424651	4.338813	
	C	3.264023	13.640427	5.520592	
	H	3.051551	13.096939	6.432983	
	C	4.300552	14.551986	5.527480	
	H	4.892204	14.713004	6.422525	

	C	0.750933	12.356396	3.043368	
	H	-0.029119	11.610608	3.198178	
	H	1.402039	12.003720	2.233248	
	H	0.269759	13.289579	2.723659	
	C	1.128720	11.764654	5.458662	
	H	1.964419	11.142454	5.802754	
	H	0.295895	11.105831	5.211599	
	H	0.815031	12.415757	6.284470	
